# Isolation and Identification of a Urinary Biomarker for Lung Cancer: 27-Nor-5β-Cholestane-3α,7α,12α,24*R*,25*S* Pentol Glucuronide and Its Deuterated Analog

**DOI:** 10.3390/molecules29122781

**Published:** 2024-06-11

**Authors:** Burchelle Blackman, Natarajan Raju, Chandrasekhar Mushti, Kelly Lane, Daxeshkumar Patel, Curtis Harris, Rolf E. Swenson

**Affiliations:** 1 Chemistry and Chemical Synthesis Center, National Heart, Lung, and Blood Institute, National Institutes of Health, 9800 Medical Center Drive, Rockville, MD 20850, USA; natarajan.raju@nih.gov (N.R.); chandrasekhar.mushti@nih.gov (C.M.); klane3@umbc.edu (K.L.); 2 AstraZeneca, 1 MedImmune Way, Gaithersburg, MD 20878, USA; 3 Laboratory of Human Carcinogenesis, Center for Cancer Research, National Cancer Institute, Bethesda, MD 20892, USA; daxeshkumar.patel@nih.gov (D.P.);

**Keywords:** lung cancer, chiral synthesis, biomarkers, 27-nor-5β-cholestane-3α,7α,12α,24,25-pentol, bile alcohol

## Abstract

An untargeted metabolomic study identified four potential lung cancer diagnostic biomarkers in human urine. One of the potential biomarkers was an unidentified feature possessing a *m*/*z* value of 561+. “561+” was isolated from human urine and tentatively identified as 27-nor-5β-cholestane-3α,7α,12α,24,25 pentol glucuronide with unknown C24,25 stereochemistry using ^1^H NMR and mass spectrometry. In a prior report, the C24,25 stereochemistry of the aglycone, 27-nor-5β-cholestane-3α,7α,12α,24,25 pentol, was found to be 24*S*,25*R* through GC analysis of the acetonide-TMS derivative. An authentic sample was prepared and found not to have the same stereochemistry as ”561+”. To identify the C24,25 stereochemistry, four C24,C25 diastereoisomeric alcohols of 27-nor-5β-cholestane-3α,7α,12α,24,25 pentol were prepared from chiral amino acids. Using an LCMS method, the C24,C25 stereochemistry of the “561+” aglycone was determined to be 24*R*,25*S*. With the correct aglycone in hand, it was coupled with glucuronic acid to complete the first reported synthesis of 27-nor-5β-cholestane-3α,7α,12α,24*R*,25S pentol glucuronide. Deuterium labeled 27-nor-5β-cholestane-3α,7α,12α,24*R*,25*S* pentol was also synthesized for use as an internal standard for MS quantitation.

## 1. Introduction

Lung cancer, known as lung carcinoma, is the most common cause of cancer deaths in the United States [1]. This is primarily because most patients are diagnosed in advanced disease states. The 5-year survival rate is between 18% and 21% [1]. Early diagnosis and treatment lead to a better prognosis. An untargeted metabolomic study identified four potential urinary metabolite biomarkers for lung cancer diagnosis [2]. The four biomarkers, creatine riboside (CR), N-acetylneuraminic acid (NANA), cortisol sulfate, and the indeterminate feature “561+”, were found to correctly identify a patient’s cancer status (+/−cancer) [2]. When “561+” was isolated from human urine, *m*/*z* 561+ was discovered to be a fragment ion of *m*/*z* 615+ (see Supplementary Materials). By searching the chemical literature and MS databases for compounds with similar fragmentation patterns, “561+” was tentatively identified as 27-nor-5β-cholestane-3α,7α,12α,24,25 pentol glucuronide of unknown C24,25 stereochemistry.

There are few reports of 27-nor-5β-cholestane-3α,7α,12α,24,25 pentol as the aglycone or the glucuronide in the literature. 27-nor-5β-cholestane-3α,7α,12α,24,25 pentol glucuronide is the major bile alcohol found in human urine [3,4,5,6,7]. The presence of 27-nor-5β-cholestane-3α,7α,12α,24,25 pentol in urine has been associated with liver disease [3,4,5,6,7,8] and disorders of lipid [9] storage. Recently, 27-nor-5β-cholestane-3α,7α,12α,24,25 pentol glucuronide, detected by LC/MS, was reported as a potential biomarker in serum for epithelium ovarian cancer by Wu and coworkers [10,11].

The prior reports did not disclose the absolute stereochemistry at C24 and C25. Therefore, in 2000, Une and coworkers [12] synthesized the four C24,C25 isomers of 27-nor-5β-cholestane-3α,7α,12α,24,25 pentol. By comparing the GC retention times of the acetonide-TMS derivatives, (24*S*,25*R*)-27-nor-5β-cholestane-3α,7α,12α,24,25-pentol was found to be the major bile alcohol, followed by a small amount of (24*R*,25*R*) in the urine of healthy individuals. This assignment was supported by the work of Dayal and coworkers [9], who assigned C24 as 24*S* based on lanthanide-induced circular dichroism Cotton effect measurements.

We intended to prepare an authentic standard 24*S*,25*R* pentol to confirm the identification of “561+” in our sample. During the course of our research, we discovered that the reported C24,C25 stereochemistry did not match “561+”. Now, we report the synthesis of the four 24,25 diastereoisomeric alcohols of 27-nor-5β-cholestane-3α,7α,12α,24,25 pentol employing a chiral pool approach and the identity of C24,C25 stereochemistry of “561+”.

## 2. Results and Discussion

**Scheme 1:**
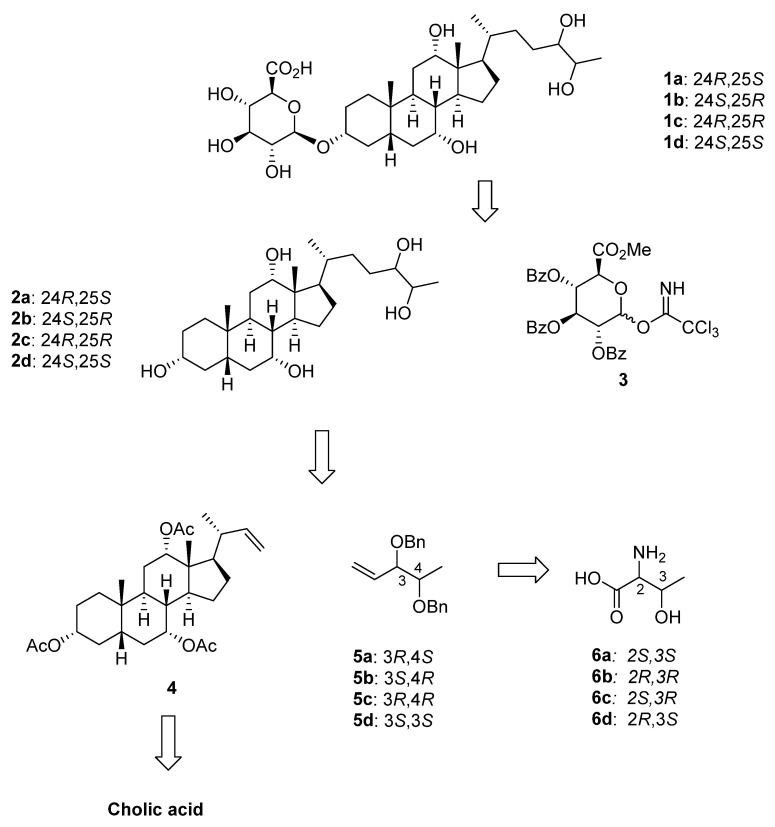
Retrosynthetic analysis for 27-nor-5β-cholestane-3α,7α,12α,24,25 pentol glucuronides **1a**–**d**.

Our approach for the synthesis of the four diastereoisomeric 27-nor-5β-cholestane-3α,7α,12α,24,25 pentols, **2a**–**d**, is shown in Scheme 1. The stereochemistry of the C24 and C25 diols was derived from the chiral 2-amino-3-hydroxy butanoic acid. Coupling with D-glucuronic acid derivative **3**, followed by deprotection would give the final compounds **1a**–**d**. The side chain of **2a**–**d** containing the C24 and C25 alcohols was introduced by a cross metathesis reaction of a suitably protected C22-alkene steroid (**4**) and chiral 3,4 dihydroxybut-1-ene (**5a**–**d**). Homochiral dihydroxybut-1-enes **5a**–**d** were prepared from commercially available chiral 2-amino-3-hydroxy butanoic acids (**6a**–**d**). The C22-alkene steroid **4** was prepared from cholic acid by known methods [13,14].

**Scheme 2:**
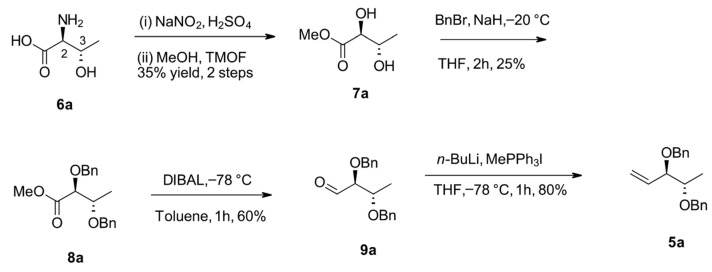
Synthesis of chiral alkene **5a**.

The synthesis of the cross metathesis precursor alkene **5a** began with the diazotization of L-allothreonine (**6a**) in water (Scheme 2), followed by the conversion of the carboxylic acid to a methyl ester with **7a** trimethyl orthoformate [15]. Diol **7a** was protected as dibenzyl ether **8a** before reduction of the methyl ester with DIBAL-H to afford aldehyde **9a** in 60% yield. Treatment of aldehyde **9a** with methyltriphenylphosphonium iodide afforded alkene **5a** in 80% yield. The remaining diastereoisomeric alkenes (**5b**–**d**) were prepared in a similar manner.

**Scheme 3:**
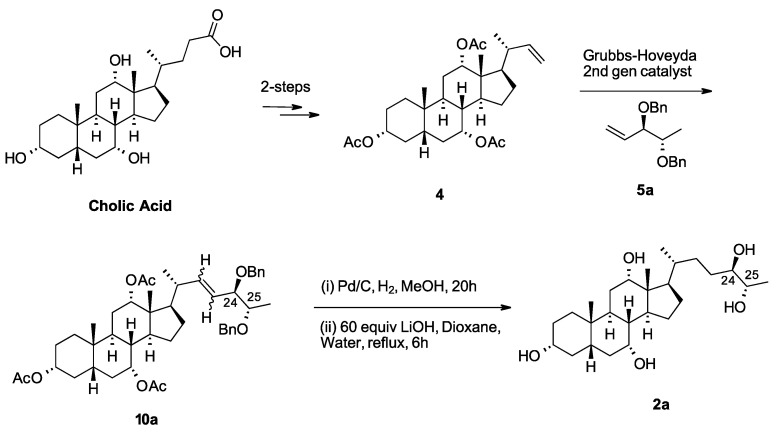
Synthesis of chiral aglycone **2a** from intermediate **4** [13,14] which is derived from cholic acid.

With the chiral alkenes **5a**–**d** synthesized, alkene **4** was prepared using reported methods [13,14]. A cross-metathesis reaction (Scheme 3), using the 2nd generation Grubbs-Hoveyda catalyst [16,17], coupled alkenes **4** and **5a**. The resulting mixtures of alkenes **10a** was hydrogenated with palladium on carbon. Finally, global deprotection afforded aglycone **2a**. The remaining diastereomeric aglycones **2b**–**d** were prepared in a similar fashion.

**Figure 1:**
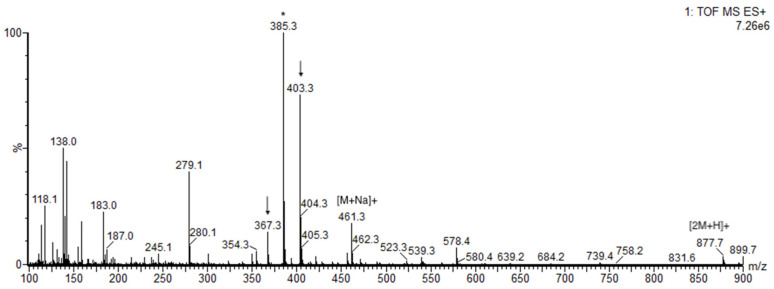
ESI+ mass spectrum of 27-nor-5β-cholestane-3α,7α,12α,24,25 pentol. Pseudomolecular ion *m*/*z* = 385.3+ used for detection and quantitation is marked with an asterisk (*). The peaks marked with an arrow are additional fragment ions derived from the parent molecular ion *m*/*z* = 439.3+ ([M + H]+).

Once the four diastereomeric aglycones **2a**–**d** were prepared, we developed an LCMS method for stereochemical assignment that, unlike prior methods, does not require derivatization. Because the aglycone readily fragments in positive electrospray ionization mass spectrometry mode, the pseudomolecular ion *m*/*z* = 385.3+ was used to extract chromatograms for detection and quantitation (Figure 1).

**Figure 2:**
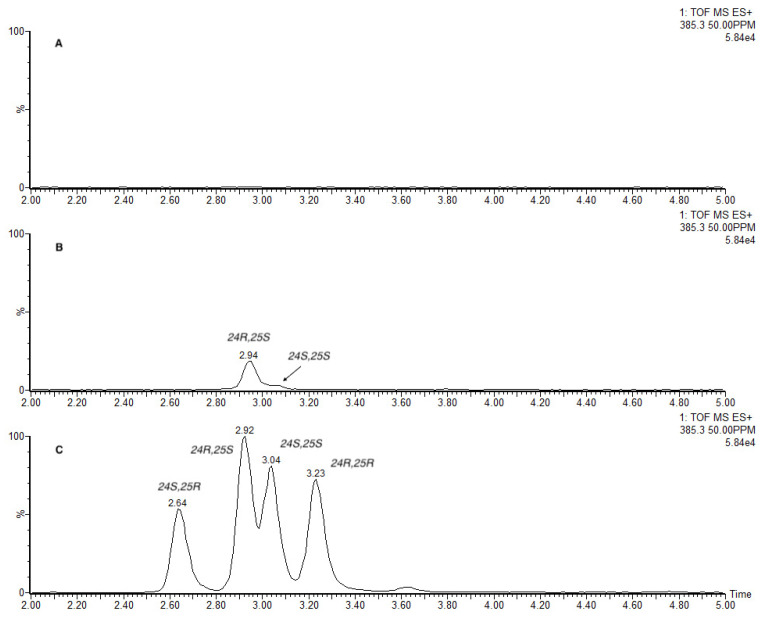
Stereochemistry of 27-nor-5β-cholestane-3α,7α,12α,24,25 pentol released from human urine treated with β-glucuronidase. ESI+ mass extracted chromatograms at *m*/*z* 358.5+. (**A**) Untreated urine. (**B**) Urine treated with β-glucuronidase. (**C**) Mixture of the four diastereoisomeric aglycones **2a**–**d**.

We found that treating human urine from lung cancer patients containing 27-nor-5β-cholestane-3α,7α,12α,24,25 pentol glucuronide with β-glucuronidase released the unexpected 24*R*,25*S* aglycone, **2a**, rather than the expected 24*S*,25*R* aglycone, **2b** (Figure 2). A small amount of 24*S*,25*S* aglycone, **2d**, was also observed. Une and coworkers [12] reported a 9:1 mixture of diastereomeric aglycones in human urine, with 24*S*,25*R*, **2a**, as the major isomer and 24*R*,25*R*, **2c**, as the minor.

**Scheme 4:**
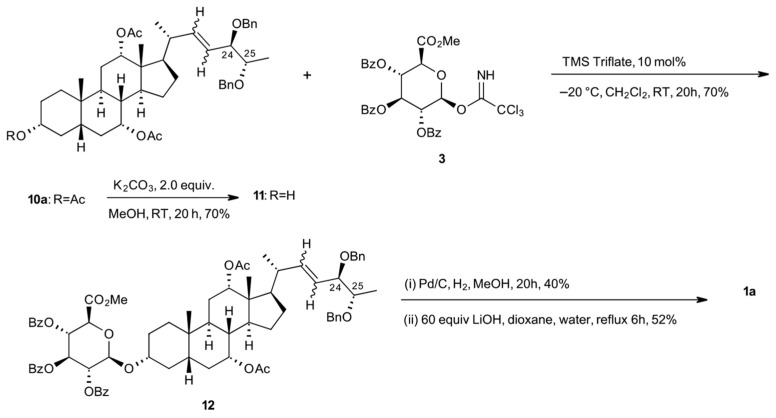
Synthesis of **1a**.

Starting from aglycone **2a**, we prepared the corresponding glucuronide, **1a** (Scheme 4). The fully protected pentol **10a**, from the cross-metathesis reaction, was selectively deprotected at the C2 position to give alcohol **11**. Treatment with trichloroacetimidate (**3)** affords fully protected glucuronide **12**. Reduction of the C22 alkene followed by global deprotection completed the first total synthesis of 27-nor-5β-cholestane-3α,7α,12α,24*R*,25*S* pentol glucuronide (**1a**).

**Figure 3:**
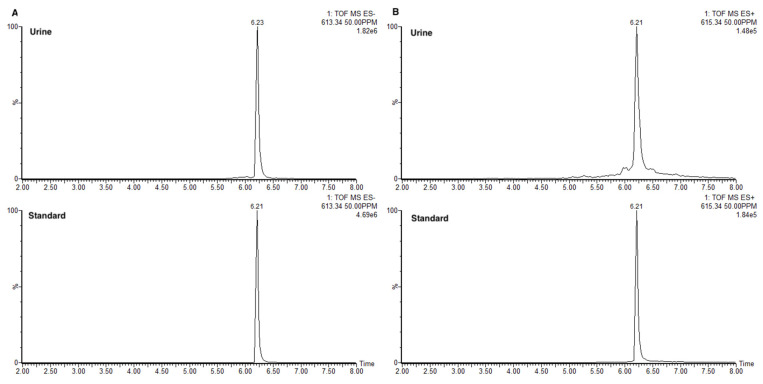
Comparison of LCMS retention times between “561+” in urine vs. standard 1a. (**A**) ESI-extracted mass chromatograms at *m*/*z* = 613.34+ (M − H). (**B**) ESI+ extracted mass chromatograms at *m*/*z* = 615.34 (M + H).

**Figure 4:**
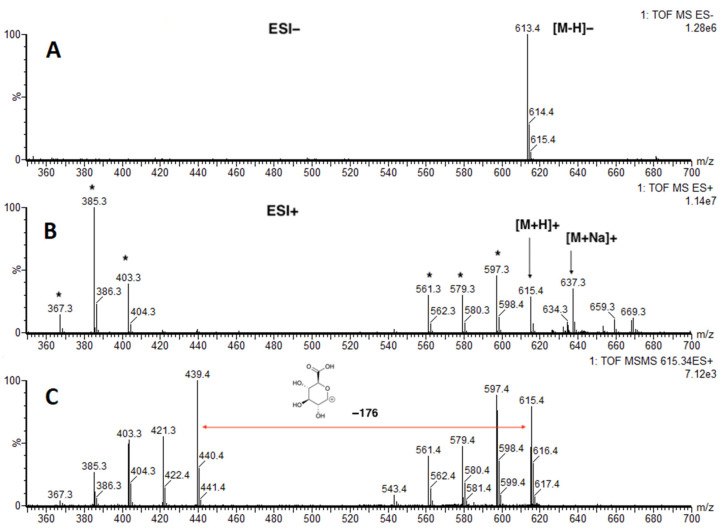
Identification of “561+”. (**A**) ESI− mass spectrum; (**B**) ESI+ mass spectrum. Peaks marked with an asterisk (*) are fragment ions derived from *m*/*z* 615.3+; (**C**) ESI+ MS/MS of *m*/*z* 615.34.

Now that we have authentic standard **1a**, the structure of “561+” was confirmed as 27-nor-5β-cholestane-3α,7α,12α,24*R*,25S pentol glucuronide (**1a**) by LCMS (Figure 3). We also discovered that *m*/*z* 561+ is a fragment ion of the molecular ion *m*/*z* = 615.4+ (Figure 4).

**Scheme 5:**
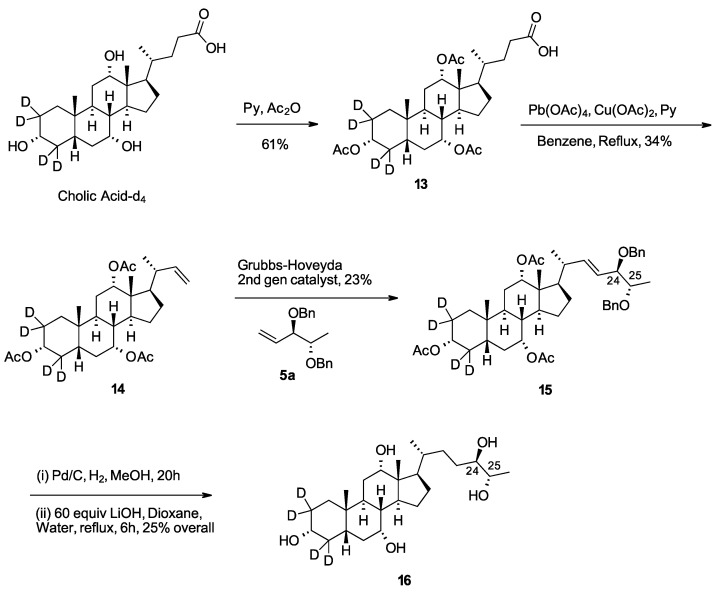
Synthesis of **16.**

To aid quantitation by mass spectrometry, the d_4_-aglycone **16** [18] was prepared from cholic acid-d_4_ following similar chemistry as reported for compound **2a** (Scheme 5). Cholic acid-d_4_ was fully protected as the triacetate **13** using acetic anhydride in pyridine. Oxidative decarboxylation gave the cross-metathesis precursor **14.** The cross metathesis of alkenes **14** and **5a** afforded **15** as a mixture of alkenes. Simultaneous debenzylation and alkene reduction followed by removal of the acetate groups gave the d_4_-labeled 27-nor-5β-cholestane-3α,7α,12α,24*R*,25*S* pentol (**16**) in five linear steps from commercially available starting materials.

## 3. Materials and Methods

### 3.1. Instrumentation

NMR spectra were recorded on a Varian 400 MHz instrument (400 MHz for ^1^H and 100 MHz for ^13^C), and all chemical shift values referred to δ TMS = 0.00 ppm (δ (1H)) and CDCl_3_ (δ (13C), 77.16 ppm). LC/MS data were secured from an Agilent 1200 LC/MS spectrometer. The HRMS analysis was achieved on a Waters Xevo G2-XS using ESI ionization.

### 3.2. Materials

All the chemicals and solvents were procured from Sigma Aldrich unless otherwise stated. Trichloroacetimidate was purchased from Toronto Chemicals Industries.

### 3.3. Isolation of “561+” from Urine from Lung Cancer Patients

561+ was purified from 500 mL of urine from lung cancer patients in three steps. First, 100–200 mL of the urine was centrifuged in 50 mL conical tubes at 3200× *g* for 10 min to pellet any particulate material. A 10 g Sep-Pak C18 was conditioned with 2 × 20 mL methanol followed by 2 × 20 mL water. The urine supernatant was loaded onto the conditioned Sep-Pak and washed sequentially with 2 × 20 mL water + 0.1% formic acid, 2 × 20 mL 50% methanol/50% water + 0.1% formic acid, and 20 mL 75% methanol/25% H_2_O + 0.1% formic acid. The ion of interest was then eluted with 3 × 20 mL methanol + 0.1% formic acid and dried by Speedvac. Next, 561+ was purified by HPLC using a Waters XBridge Prep C18 (10 µm, 10 × 250 mm) run at 4 mL/min at a gradient of 2–20% B (0–10 min), 20–95% B (10–37 min), and 95% B (37–42 min) (A = water + 0.1% formic acid, B = acetonitrile + 0.1% formic acid). 561+ was eluted in the second gradient, and fractions containing this ion were dried by Speedvac. Lastly, 561+ was further purified by analytical HPLC using a Waters XBridge C18 column (5 µm, 4.6 × 150 mm) run at 1 mL/min at a gradient of 50% B (0–3 min), 50–95% B (3–23 min), and 95% B (23–26 min) (A = H_2_O + 0.1% formic acid, B = Methanol + 0.1% formic acid). 561+ eluted late in the second gradient. The final compound was dried by Speedvac.

### 3.4. Synthesis

#### 3.4.1. General Procedure for the Synthesis of Methyl Dihydroxybutyrates (**7a**–**d**)

Methyl (2*S*,3*S*)-2,3-dihydroxybutanoate (**7a**). To an ice-cold solution of L-allothreonine (11.9 g, 0.1 mol) in water (25.0 mL) and 2N sulfuric acid (51.0 mL, 0.102 mol), sodium nitrite in water (13.8 g, 0.101 mol in 20 mL) was added dropwise over a 1 h period at 0 °C with vigorous stirring. After the addition, the solution was allowed to come to room temperature and stirred for 20 h. All the volatiles were removed under reduced pressure at room temperature, then the residue was diluted with 1:1 MeOH*/i*-PrOH (20.0 mL) and filtered. The filtrate was concentrated again to a thick syrup, and the crude product was taken to the next step.

The crude dihydroxy acid (10.0 g) was dissolved in 250 mL of anhydrous methanol and stirred under argon. Trimethyl orthoformate (50 mL) was added, followed by IR-120 acid resin (50.0 g), and stirred at room temperature for 72 h. The resin was filtered and washed with methanol (3 × 50 mL), and the filtrates were combined and evaporated under reduced pressure to yield a brown oil. Chromatography of the crude ester using 220.0 g of flash silica (Teledyne-Isco) and elution with 6:4 ethyl acetate/hexanes yielded **7a** as colorless oil. Yield: 4.8 g (35%). ^1^H NMR (400 MHz, CDCl_3_) δ 4.21 (d, *J* = 8 Hz, 1H), 4.03 (m, 1H), 3.72 (s, 3H), 3.10 (bs, 2H), 1.10 (d, *J* = 12 Hz, 3H). ^13^C NMR (101 MHz, CDCl_3_) δ 173.04, 74.60, 69.06, 52.39, 17.21.

Methyl (2*R*,3*R*)-2,3-dihydroxybutanoate (**7b**) [19,20,21]: ^1^H NMR (400 MHz, CDCl_3_) δ 4.22 (d, *J* = 3.6 Hz, 1H), 4.07–4.03 (m, 1H), 3.79 (s, 3H), 3.12 (s, 2H), 1.17 (d, *J* = 6.5 Hz, 3H); 13C NMR (101 MHz, CDCl_3_) δ 17.21, 52.32, 69.07, 74.54, 173.10. ^13^C NMR (101 MHz, CDCl_3_) δ 173.10, 74.54, 69.07, 52.53, 17.32.

Methyl (2*S*,3*R*)-2,3-dihydroxybutanoat*e* (**7c**) [22]: ^1^H NMR (400 MHz, CDCl_3_) δ 4.09 (qd, *J* = 6.5, 2.7 Hz, 1H), 4.02 (d, *J* = 2.8 Hz, 1H), 3.82 (s, 3H), 2.87 (d, *J* = 4.9 Hz, 3H), 1.30 (d, *J* = 6.5 Hz, 3H). ^13^C NMR (101 MHz, CDCl_3_) δ 173.77, 74.38, 68.68, 52.78, 19.58.

Methyl (2*R*,3*S*)-2,3-dihydroxybutanoate (**7d**) [23]: ^1^H NMR (400 MHz, CDCl_3_) δ 4.12–4.03 (m, 1H), 4.02 (d, *J* = 2.7 Hz, 1H), 3.81 (s, 3H), 2.80 (bs, 2H), 1.30 (d, *J* = 6.5 Hz, 3H). ^13^C NMR (100 MHz, CDCl_3_) δ 19.57, 52.76, 68.47, 74.47, 173.79.

#### 3.4.2. General Procedure for the Synthesis of Methyl-bis-O-benzylbutyrate (**8a**–**d**)

Methyl (2*S*,3*S*)-2,3-bis(benzyloxy)butanoate (**8a**). Sodium hydride (60% in oil, 2.88 g, 72.0 mmol) was suspended in 50.0 mL of anhydrous DMF and stirred under argon at −20 °C. The dihydroxy ester **7a** (4.8 g, 35.8 mmol) in anhydrous DMF (50.0 mL) was added dropwise to the stirred suspension over a 1 h period. The stirring was continued for 1 h more at −20 °C, and benzyl bromide (12.31 g, 72.0 mmol) was added dropwise with stirring over 1 h. The reaction was continued for 2 h more after the addition at −20 °C. Saturated ammonium chloride (50 mL) was carefully added to the reaction mixture and diluted with 500 mL of water. The solution was extracted with ethyl acetate (3 × 150 mL). The combined extracts were washed with water (2 × 200 mL), saturated sodium chloride (1 × 200 mL), and dried over Na_2_SO_4_. The solution was filtered, concentrated under reduced pressure, and the resulting oil was purified using 120.0 g of flash silica. Elution with 10% ethyl acetate in hexanes yielded **8a** as colorless oil. Yield: 2.8 g (25%). ^1^H NMR (400 MHz, CDCl_3_) δ 7.3 (m, 1H), 4.7 (AB q, 2H), 4.6 (m, 2H), 4.1 (d, 1H, *J* = 2 Hz), 3.9 (m, 1H), 3.77 (3H), 1.3 (d, *J* = 12 Hz, 3H). ^13^C NMR (101 MHz, CDCl_3_) δ 16.38, 51.91, 51.93, 71.29, 72.66, 75.83, 127.59, 127.54, 127.59, 127.68, 127.91, 128.06, 128.33, 128.41, 171.65.

Methyl (2*R*,3*R*)-2,3-bis(benzyloxy)butanoate (**8b**): ^1^H NMR (400 MHz, CDCl_3_) δ 7.44–7.22 (m, 1H), 4.73 (d, *J* = 11.9 Hz, 2H), 4.59–4.44 (m, 3H), 4.02 (d, *J* = 5.2 Hz, 1H), 3.88 (qd, *J* = 6.3, 5.2 Hz, 1H), 3.74 (s, 3H), 1.29 (d, *J* = 6.3 Hz, 3H). ^13^C NMR (101 MHz, CDCl_3_) δ 171.64, 138.24, 137.30, 128.37, 128.29, 128.03, 127.88, 127.64, 127.55, 81.03, 75.78, 72.63, 71.26, 51.90, 16.33.

Methyl (2*S*,3*R*)-2,3-bis(benzyloxy)butanoate (**8c**): ^1^H NMR (400 MHz, CDCl_3_) δ 7.41–7.17 (m, 1H), 4.81 (d, *J* = 11.9 Hz, 1H), 4.62 (d, *J* = 11.9 Hz, 1H), 4.57–4.41 (m, 2H), 3.96 (dd, *J* = 4.4, 1.1 Hz, 1H), 3.89 (td, *J* = 6.5, 4.7 Hz, 1H), 3.71 (s, 3H), 1.22 (d, *J* = 6.4 Hz, 3H). ^13^C NMR (101 MHz, CDCl_3_) δ 171.29, 138.33, 137.31, 128.35, 128.25, 128.18, 127.89, 127.83, 127.54, 81.34, 75.03, 72.84, 71.44, 51.80, 15.86.

Methyl (2*R*,3*S*)-2,3-bis(benzyloxy)butanoate (**8d**): ^1^H NMR (400 MHz, CDCl_3_) δ 7.37–7.23 (m, 1H), 4.81 (d, *J* = 12.0 Hz, 1H), 4.62 (d, *J* = 12.0 Hz, 1H), 4.53 (d, *J* = 12.0 Hz, 1H), 4.46 (d, *J* = 12.0 Hz, 1H), 3.95 (d, *J* = 4.5 Hz, 1H), 3.89 (td, *J* = 6.3, 4.4 Hz, 1H), 1.22 (d, *J* = 6.3 Hz, 3H). ^13^C NMR (101 MHz, CDCl_3_) δ 171.30, 138.33, 137.32, 128.36, 128.26, 128.19, 127.90, 127.85, 127.56, 81.33, 75.03, 72.85, 71.44, 51.82, 15.87.

#### 3.4.3. General Procedure for the Synthesis of bis-O-Benzyl-1-butanal (**9a**–**d**)

(2*S*,3*S*)-2,3-bis(benzyloxy)butanal (**9a**). A solution of 2*S*, 3*S*-bis-O-benzyl ester **8a** (6.0 g, 219.1 mmol) in anhydrous toluene (20.0 mL) was cooled to −70 °C under argon, and DIBAL (1M in toluene, 30.6 mL, 30.6 mmol) was added dropwise over a period of 1 h with vigorous stirring. After the addition, the reaction was continued for 1 h more at −70 °C and then carefully quenched with the dropwise addition of methanol (10.0 mL) at −70 °C. The reaction mixture was diluted with toluene (50.0 mL), and the organic layer was washed with 2M HCl until the toluene layer was clear of the aluminum salts. The organic layer was washed with water (3 × 50 mL) and dried (Na_2_SO_4_). The organic layer was filtered, concentrated under reduced pressure at RT, and the resulting oil was purified by a silica gel column (pre-packed 120.0 g, Teledyne-Isco). Elution with 10% ether in hexanes yielded aldehyde **9a** as colorless oil. Yield: 3.2 g (59%). ^1^H NMR (400 MHz, CDCl_3_) δ 9.76 (d, *J* = 1.5 Hz), 7.39–7.22 (m, 10H), 5.95–5.81 (m, 1H), 5.39–5.26 (m, 2H), 4.67 (d, *J* = 12.1 Hz, 1H), 4.61 (s, 2H), 4.43 (d, *J* = 12.1 Hz, 1H), 3.79 (ddt, *J* = 7.5, 4.4, 0.9 Hz, 1H), 3.65 (qd, *J* = 6.3, 4.4 Hz, 1H), 1.22 (d, *J* = 6.4 Hz, 3H). ^13^C NMR (101 MHz, CDCl_3_) δ 16.24, 70.44, 70.46, 71.36, 77.18, 83.40, 118.83, 127.38, 127.40, 127.64, 127.66, 128.27, 128.30, 135.84, 138.73, 138.95.

(2*R*,3*R*)-2,3-bis(benzyloxy)butanal (**9b**): ^1^H NMR (400 MHz, CDCl_3_) δ 9.71 (d, *J* = 2.0 Hz, 1H), 7.40–7.26 (m, 10H), 4.72 (d, *J* = 11.8 Hz, 1H), 4.67–4.60 (m, 2H), 4.54 (d, *J* = 11.9 Hz, 1H), 3.92 (qd, *J* = 6.4, 4.3 Hz, 1H), 3.85 (dd, *J* = 4.3, 2.0 Hz, 1H), 1.30 (d, *J* = 6.4 Hz, 3H). ^13^C NMR (101 MHz, CDCl_3_) δ 202.61, 138.07, 137.32, 128.52, 128.41, 128.06, 128.04, 127.70, 127.63, 85.73, 75.32, 72.95, 71.17, 16.18.

(2*S*,3*R*)-2,3-bis(benzyloxy)butanal (**9c**): ^1^H NMR (400 MHz, CDCl_3_) δ 9.74 (d, *J* = 1.5 Hz, 1H), 7.41–7.20 (m, 10H), 4.79 (d, *J* = 12.0 Hz, 1H), 4.67–4.39 (m, 4H), 3.93 (qd, *J* = 6.4, 4.3 Hz, 1H), 3.78 (dd, *J* = 4.2, 1.5 Hz, 1H), 1.26 (d, *J* = 6.4 Hz, 4H). ^13^C NMR (101 MHz, CDCl_3_) δ 203.38, 137.89, 137.14, 128.51, 128.37, 128.18, 128.13, 127.80, 127.74, 85.43, 74.80, 73.21, 71.36, 15.70.

(2*R*,3*S*)-2,3-bis(benzyloxy)butanal (**9d**): ^1^H NMR (400 MHz, CDCl_3_) δ 9.76 (d, *J* = 1.5 Hz, 1H), 7.39–7.23 (m, 10H), 4.80 (d, *J* = 12.0 Hz, 1H), 4.67–4.45 (m, 3H), 3.94 (qd, *J* = 6.4, 4.3 Hz, 1H), 3.80 (dd, *J* = 4.2, 1.5 Hz, 1H), 1.28 (d, *J* = 6.4 Hz, 3H). ^13^C NMR (101 MHz, CDCl_3_) δ 202.65, 138.06, 137.31, 128.52, 128.41, 128.06, 128.02, 127.70, 127.64, 85.53, 75.30, 72.91, 71.21, 16.18.

#### 3.4.4. General Procedure for the Synthesis of bis-O-Benzylbut-1-ene (**5a**–**d**)

((((2*S*,3*R*)-pent-4-ene-2,3-diyl)bis(oxy))bis(methylene))dibenzene (**5a**). Methyl triphenylphosphonium iodide (1.55 g, 3.84 mmol) in anhydrous THF (20 mL) was cooled to −78 °C under argon and stirred. *n*-BuLi (1.6 M in hexanes, 2.2 mL, 3.51 mmol) was added dropwise and stirred at −78 °C for 1 h, allowed to come to 0 °C, and stirred for an additional 1 h. The orange solution of the phosphorane was cooled back to −78 °C, and aldehyde **9a** (0.9 g, 3.19 mmol) in anhydrous THF (5.0 mL) was added dropwise over 15 min and the reaction was stirred at −78 °C for 2 h, allowed to come to 0 °C, and the reaction was continued for 16 h more. Saturated ammonium chloride (5.0 mL) was added to quench the reaction and diluted with 50 mL of ethyl acetate. The organic layer was washed with water (3 × 25 mL), saturated sodium chloride (1 × 50 mL), and dried over Na_2_SO_4_. The solution was filtered, concentrated under reduced pressure at room temperature, and the residue chromatographed using 40 g of flash silica. Elution with 5% ethyl acetate in hexanes yielded olefin **5a** as a colorless oil. Yield: 0.715 g (80%). ^1^H NMR (400 MHz, CDCl_3_) δ 7.39–7.18 (m, 10H), 5.87 (ddd, *J* = 17.3, 10.5, 7.5 Hz, 1H), 5.38–5.20 (m, 2H), 4.65 (dq, *J* = 12.1, 0.6 Hz, 1H), 4.59 (d, *J* = 0.5 Hz, 2H), 4.42 (dd, *J* = 12.1, 0.5 Hz, 1H), 3.78 (ddt, *J* = 7.5, 4.4, 1.0 Hz, 1H), 3.63 (qd, *J* = 6.4, 4.4 Hz, 1H), 1.21 (d, *J* = 6.4 Hz, 3H). ^13^C NMR (101 MHz, CDCl_3_) δ 138.97, 138.74, 135.86, 128.45, 127.79, 127.38, 118.81, 83.42, 77.19, 71.40, 70.44, 16.25.

((((2*R*,3*S*)-pent-4-ene-2,3-diyl)bis(oxy))bis(methylene))dibenzene (**5b**) [24] ^1^H NMR (400 MHz, CDCl3) δ 7.41–7.24 (m, 10H), 5.90 (ddd, *J* = 17.2, 10.5, 7.5 Hz, 1H), 5.41–5.25 (m, 2H), 4.68 (dd, *J* = 12.1, 0.6 Hz, 1H), 4.63 (d, *J* = 0.5 Hz, 2H), 4.45 (dd, *J* = 12.2, 0.5 Hz, 1H), 3.81 (ddt, *J* = 7.5, 4.3, 0.9 Hz, 1H), 3.66 (qd, *J* = 6.4, 4.4 Hz, 1H), 1.24 (d, *J* = 6.4 Hz, 3H). ^13^C NMR (101 MHz, CDCl_3_) δ 138.93, 138.71, 135.82, 128.27, 128.24, 127.63, 127.61, 127.37, 127.35, 118.79, 83.39, 77.15, 71.33, 70.44, 16.21.

((((2*R*,3*R*)-pent-4-ene-2,3-diyl)bis(oxy))bis(methylene))dibenzene (**5c**) ^1^H NMR (400 MHz, CDCl_3_) δ 7.39–7.25 (m, 10H), 5.94–5.78 (m, 1H), 5.38–5.26 (m, 1H), 4.65 (d, *J* = 11.9 Hz, 3H), 4.43 (d, *J* = 12.0 Hz, 1H), 3.86 (ddt, *J* = 7.5, 5.8, 1.0 Hz, 1H), 3.71–3.56 (m, 1H), 1.16 (d, *J* = 6.4 Hz, 3H). ^13^C NMR (101 MHz, CDCl_3_) δ 138.97, 138.67, 135.27, 128.27, 128.25, 127.67, 127.65, 127.40, 127.38, 118.64, 83.44, 76.84, 71.73, 70.58, 16.12.

((((2*S*,3*S*)-pent-4-ene-2,3-diyl)bis(oxy))bis(methylene))dibenzene (**5d**) ^1^H NMR (400 MHz, CDCl_3_) δ 7.34–7.08 (m, 10H), 5.75 (ddd, *J* = 16.7, 10.9, 7.5 Hz, 1H), 5.30–5.17 (m, 2H), 4.57 (d, *J* = 12.2 Hz, 3H), 4.35 (d, *J* = 12.0 Hz, 1H), 3.77 (ddt, *J* = 7.5, 5.8, 1.0 Hz, 1H), 3.66–3.48 (m, 1H), 1.08 (d, *J* = 6.4 Hz, 3H). ^13^C NMR (101 MHz, CDCl_3_) δ 138.97, 138.68, 135.28, 128.28, 127.64, 127.39, 118.66, 83.45, 76.84, 71.74, 70.59, 16.13.

#### 3.4.5. General Procedure for the Synthesis of Bisbenzyl Aglycones (**10a**–**d**)

(3*R*,5*S*,7*R*,8*R*,9*S*,10*S*,12*S*,13*R*,14*S*,17*R*)-17-((2*R*,5*R*,6*S*)-5,6-bis(benzyloxy)hept-3-en-2-yl)-10,13-dimethylhexadecahydro-1H-cyclopenta[a]phenanthrene-3,7,12-triyl triacetate (**10a**). Olefins **4** [25,26,27,28] (0.15 g, 0.31 mmol) and **5a** (0.434 g, 1.55 mmol) were dissolved in 5.0 mL of benzene and degassed with argon for 5.0 min. Hoveyda-Grubb’s 2nd generation catalyst (97.0 mg, 10 mol%) was added to the mixture and refluxed under argon for 20 h. The crude reaction mixture was adsorbed onto 5.0 g of silica gel and dried under vacuum. The dry powder was loaded onto a loading cartridge and purified on a 24.0 g flash silica column. Elution with 25% ethyl acetate in hexanes yielded **10a** as a pale brown gum that was contaminated with other olefinic side products. It was taken to the next step without further purification. Yield: 69.0 mg (30%, partially purified).

#### 3.4.6. General Procedure for the Synthesis of 27-Nor-5β-Cholestane-3α,7α,12α,24,25 Pentol (**2a**–**d**)

(3*R*,5*S*,7*R*,8*R*,9*S*,10*S*,12*S*,13*R*,14*S*,17*R*)-17-((2*R*,5*R*,6*S*)-5,6-dihydroxyheptan-2-yl)-10,13-dimethylhexadecahydro-1H-cyclopenta[a]phenanthrene-3,7,12-triol (**2a**) [12]. Compound **10a** (69.0 mg, 0.093 mmol) was hydrogenated using a balloon for 20 h in the presence of 10%Pd/C (14.0 mg) in methanol for 20 h at room temperature. The catalyst was filtered off, and the filtrate was concentrated into a paste. The crude hydrogenated product was refluxed with 1M KOH in methanol (6.0 mL, 60.0 equiv.) for 6 h. The pH of the deprotected product was adjusted to about 5.0 with IR-120 acid resin. The resin was filtered off, and the filtrate was concentrated into a paste and chromatographed using 12.0 g of flash silica. Elution with 10% methanol in chloroform yielded **2a** as a colorless solid. Yield: 20.3 mg (50%). ^1^H NMR (400 MHz, CD_3_OD) δ 3.96 (t, *J* = 2.9 Hz, 1H), 3.79 (d, *J* = 3.1 Hz, 1H), 3.53 (td, *J* = 6.4, 5.5 Hz, 1H), 3.42–3.33 (m, 1H), 2.34–2.20 (m, 2H), 2.04–1.21 (m, 21H), 1.15 (d, *J* = 6.4 Hz, 3H), 1.12–1.05 (m, 1H), 1.02 (d, *J* = 6.5 Hz, 3H), 0.96 (dd, *J* = 14.1, 3.5 Hz, 1H), 0.91 (s, 3H), 0.72 (s, 3H). ^13^C NMR (101 MHz, CD_3_OD) δ 77.11, 74.17, 72.95, 71.96, 69.14, 47.51, 43.27, 43.05, 41.10, 40.54, 37.14, 36.55, 35.96, 33.36, 31.24, 30.46, 29.64, 28.86, 27.94, 24.30, 23.21, 18.63, 18.05, 13.06. HRMS (ESI) calcd for C_26_H_46_O_4_Na [M + Na]^+^ 461.3243; found 461.3228.

(3*R*,5*S*,7*R*,8*R*,9*S*,10*S*,12*S*,13*R*,14*S*,17*R*)-17-((2*R*,5*S*,6*R*)-5,6-dihydroxyheptan-2-yl)-10,13-dimethylhexadecahydro-1H-cyclopenta[a]phenanthrene-3,7,12-triol (**2b**) [12]: ^1^H NMR (400 MHz, CD_3_OD) δ 3.96 (t, *J* = 3.0 Hz, 1H), 3.79 (q, *J* = 3.0 Hz, 1H), 3.68 (s, 1H), 3.56 (dd, *J* = 6.4, 5.1 Hz, 1H), 3.41–3.23 (m, 3H), 2.34–2.16 (m, 2H), 2.04–1.17 (m, 18H), 1.14 (d, *J* = 6.4 Hz, 3H), 1.12–1.05 (m, 1H), 1.03 (d, *J* = 6.6 Hz, 3H), 0.96 (dd, *J* = 14.1, 3.4 Hz, 1H), 0.91 (s, 3H), 0.71 (s, 3H). ^13^C NMR (101 MHz, CD_3_OD) δ 76.09, 72.68, 71.46, 70.27, 67.66, 46.87, 46.01, 41.78, 41.56, 39.61, 39.05, 36.09, 35.08, 32.14, 29.76, 29.21, 28.17, 27.37, 26.46, 22.83, 21.74, 16.81, 16.71, 11.59. HRMS (ESI) calcd for C_26_H_46_O_4_Na [M + Na]^+^ 461.3243; found 461.3227.

(3*R*,5*S*,7*R*,8*R*,9*S*,10*S*,12*S*,13*R*,14*S*,17*R*)-17-((2*R*,5*R*,6*R*)-5,6-dihydroxyheptan-2-yl)-10,13-dimethylhexadecahydro-1H-cyclopenta[a]phenanthrene-3,7,12-triol (**2c**) [12]: ^1^H NMR (400 MHz, CD_3_OD) δ 3.96 (t, *J* = 3.0 Hz, 1H), 3.79 (q, *J* = 3.0 Hz, 1H), 3.63–3.52 (m, 1H), 3.41–3.33 (m, 1H), 3.32–3.23 (m, 4H), 2.34–2.19 (m, 2H), 2.03–1.22 (m, 15H), 1.13 (d, *J* = 6.4 Hz, 3H), 1.07 (dd, *J* = 12.0, 6.0 Hz, 3H), 0.98 (d, *J* = 3.4 Hz, 3H), 0.94 (d, *J* = 3.4 Hz, 3H), 0.91 (s, 3H), 0.72 (s, 3H). ^13^C NMR (101 MHz, CD_3_OD) δ 75.56, 72.69, 71.47, 70.20, 67.66, 46.04, 41.79, 41.57, 39.61, 39.05, 35.67, 35.07, 34.49, 34.43, 31.72, 29.76, 28.69, 28.16, 27.37, 26.46, 22.82, 21.74, 17.61, 16.54, 11.59. HRMS (ESI) calcd for C_26_H_46_O_4_Na [M + Na]^+^ 461.3243; found 461.3232.

(3*R*,5*S*,7*R*,8*R*,9*S*,10*S*,12*S*,13*R*,14*S*,17*R*)-17-((2*R*,5*S*,6*S*)-5,6-dihydroxyheptan-2-yl)-10,13-dimethylhexadecahydro-1H-cyclopenta[a]phenanthrene-3,7,12-triol (**2d**) [12]: ^1^H NMR (400 MHz, CD_3_OD) δ 4.01–3.92 (m, 1H), 3.81 (q, *J* = 3.0 Hz, 1H), 3.61 (qd, *J* = 6.4, 4.9 Hz, 1H), 3.45–3.30 (m, 3H), 3.26 (ddd, *J* = 8.2, 5.0, 3.1 Hz, 1H), 3.01 (s, 1H), 2.87 (d, *J* = 0.7 Hz, 1H), 2.36–2.20 (m, 2H), 2.06–1.19 (m, 17H), 1.15 (d, *J* = 6.4 Hz, 3H), 1.13–1.06 (m, 1H), 1.04 (d, *J* = 6.6 Hz, 3H), 0.98 (dd, *J* = 14.2, 3.5 Hz, 1H), 0.93 (s, 3H). ^13^C NMR (101 MHz, CD_3_OD) δ 76.62, 75.40, 73.91, 71.60, 50.79, 49.94, 45.72, 45.50, 43.55, 42.99, 40.02, 39.01, 38.42, 38.37, 35.98, 33.70, 32.81, 32.11, 31.31, 30.40, 26.76, 25.68. HRMS (ESI) calcd for C_26_H_46_O_4_Na [M + Na]^+^ 461.3243; found 461.3230.

#### 3.4.7. Synthesis of 27-Nor-5β-Cholestane-3α,7α,12α,24,25 Pentol Glucuronides (**1a**)

(3*R*,5*S*,7*R*,8*R*,9*S*,10*S*,12*S*,13*R*,14*S*,17*R*)-17-((2*R*,5*R*,6*S*)-5,6-bis(benzyloxy)hept-3-en-2-yl)-3-hydroxy-10,13-dimethylhexadecahydro-1H-cyclopenta[a]phenanthrene-7,12-diyl diacetate (**11**). A solution of **10a** (0.15 g, 0.2 mmol) in methanol was stirred with potassium carbonate (70.0 mg, 0.5 mmol) for 6 h at RT. Analysis by LC/MS indicated the complete consumption of the starting material. The solution was concentrated and chromatographed over 12.0 g of flash silica. Elution with 2% methanol in dichloromethane yielded **11** as a colorless gum. Yield: 0.12 g (85%). It was taken to the next step without further purification. ^1^H NMR (400 MHz, CDCl_3_) δ 7.37–7.27 (m, 10H), 7.27–7.21 (m, 4H), 5.52–5.35 (m, 2H), 5.08 (d, *J* = 3.0 Hz, 1H), 4.89 (d, *J* = 3.2 Hz, 1H), 4.64 (s, 1H), 4.60 (d, *J* = 4.3 Hz, 3H), 4.38 (d, *J* = 12.2 Hz, 1H), 3.70–3.56 (m, 2H), 3.55–3.45 (m, 1H), 2.14 (s, 5H), 2.07 (s, 4H), 2.04–1.88 (m, 3H), 1.85 (dt, *J* = 11.9, 6.0 Hz, 1H), 1.80–1.70 (m, 3H), 1.64 (d, *J* = 7.7 Hz, 3H), 1.62 (d, *J* = 5.0 Hz, 3H), 1.54–1.39 (m, 3H), 1.38–0.93 (m, 13H), 0.91 (s, 4H), 0.74 (s, 3H). ^13^C NMR (101 MHz, CDCl_3_) δ 170.59, 141.72, 128.21, 127.59, 127.31, 125.00, 83.09, 75.26, 71.73, 71.38, 70.81, 69.87, 47.17, 45.02, 43.46, 41.03, 39.30, 38.67, 37.75, 34.86, 34.30, 31.35, 30.46, 29.03, 27.73, 25.61, 22.83, 22.55, 21.62, 20.23, 16.42, 12.50.

(2*R*,3*R*,4*S*,5*S*,6*S*)-2-(((3*R*,5*S*,7*R*,8*R*,9*S*,10*S*,12*S*,13*R*,14*S*,17*R*)-7,12-diacetoxy-17-((2*R*,5*R*,6*S*)-5,6-bis(benzyloxy)hept-3-en-2-yl)-10,13-dimethylhexadecahydro-1H-cyclopenta[a]phenanthren-3-yl)oxy)-6-(methoxycarbonyl)tetrahydro-2H-pyran-3,4,5-triyl tribenzoate (**12**). Alcohol **11** (0.12 g, 0.17 mmol) and trichloroacetimidate of 3,4,5-tribenzoylglucuronic acid methyl ester, 3 (0.319 g, 0.48 mmol) were dissolved in 5.0 mL of anhydrous dichloromethane and stirred with 2.0 g of 4Å mol sieves for 1 h. The solution was cooled to −40 °C, and TMS triflate (11.0 mg, 0.048 mmol, 10 mol%) was added and stirred under argon for 2 h, allowed to come to RT, and continued for 16 more hours. The reaction was quenched with 1.0 mL of triethylamine, adsorbed onto 5.0 g of silica gel, and dried to a powder. The crude product with silica was loaded onto an empty cartridge, connected to another cartridge of 24.0 g of flash silica gel, and chromatographed. Elution with 10% ethyl acetate in toluene yielded the product contaminated with sugar impurities that were found to be difficult to separate using column chromatography. The partially purified material was taken to the next step without further purification. Yield: 30.0 mg (14.6%, impure).

(2*S*,3*S*,4*S*,5*R*,6*S*)-6-(((3*R*,5*R*,7*R*,8*R*,9*S*,10*S*,12*S*,13*R*,14*S*,17*R*)-17-((2*R*,5*R*,6*S*)-5,6-dihydroxyheptan-2-yl)-7,12-dihydroxy-10,13-dimethylhexadecahydro-1H-cyclopenta[a]phenanthren-3-yl)oxy)-3,4,5-trihydroxytetrahydro-2H-pyran-2-carboxylic acid (**1a**). A solution of the partially purified glycosylated product **12** (30.0 mg, 0.025 mmol) in methanol (5.0 mL) was hydrogenated at RT using a balloon filled with hydrogen in the presence of 10%Pd/C (10.0 mg) for 20 h. The crude product was filtered off the catalyst and concentrated into a paste. It was redissolved in dioxane (5.0 mL) and refluxed with 1M LiOH (3.0 mL) for 6 h. The pH was adjusted to about 8.0 with AG^®^ 1-X8 acid resin and filtered. The filtrate was purified by preparative HPLC [conditions: column—10 × 150 Waters C18 OBD; 10.0 micron; 130 Å solvent A—water; solvent B—methanol with 0.05% ammonium hydroxide; detection @ 210 nm; initial gradient—20% B for 5 min; 20–100% B over 50 min; elution rate—10.0 mL/min]. 3.0 mL fractions were collected and analyzed by LC/MS [conditions: Agilent Zorbax C8; 300 Å; 3.5 microns; 4.6 × 50 mm; solvent A—water; solvent B—5 mM ammonium acetate in methanol; gradient—50–100%B over 4.0 min and 3 more min at 100%B]. Fractions with the product were pooled and freeze died to yield the required product **1a** as colorless fluffy solid. Yield: 8.0 mg, 52% for two steps). ^1^H NMR (400 MHz, CD_3_OD) δ 4.40 (d, J = 7.7 Hz, 1H), 3.96 (t, J = 2.9 Hz, 1H), 3.79 (m, 1H), 3.64 (m, 1H), 3.55 (m, 2H), 3.48–3.34 (m, 4H), 3.17 (m, 1H), 2.30 (m, 2H), 1.86 (m, 8H), 1.47 (m, 10H), 1.35–1.24 (m, 2H), 1.15 (d, J = 6.4 Hz, 3H), 1.10 (dt, J = 11.9, 6.0 Hz, 1H), 1.02 (d, J = 6.5 Hz, 3H), 0.91 (s, 3H), 0.71 (s, 3H). ^13^C NMR (101 MHz, CD_3_OD) δ 11.56, 16.58, 17.14, 21.67, 22.84, 26.11, 26.44, 27.39, 28.13, 28.99, 31.88, 34.39, 34.62, 34.81, 35.68, 37.00, 39.56, 41.59, 41.72, 46.04, 67.68, 70.48, 72.31, 72.72, 73.58, 74.83, 75.64, 76.41, 78.21, 100.45, 175.02. HRMS (ESI) calcd for C_32_H_55_O_11_ [M + H]^+^ 615.3745; found 615.3748

#### 3.4.8. Synthesis of d_4_-Labeled Aglycone

3,7,12-Triacetyl cholic acid-d_4_ (**13**). Cholic acid-d_4_ (2.00 g, 1 Eq, 4.85 mmol) in pyridine (4.83 g, 4.94 mL, 12.6 Eq, 61.1 mmol) was cooled to 0 °C, and acetic anhydride (10.7 g, 9.92 mL, 21.7 Eq, 105 mmol) was added and stirred. DMAP (195 mg, 0.33 Eq, 1.60 mmol) was added to the reaction mixture and stirred at room temperature overnight. The reaction mixture was concentrated at room temperature under vacuum, diluted with ethyl acetate, washed three times with 1M HCL and water, and dried over MgSO_4_. The solution was filtered and concentrated, and the residue was chromatographed over flash silica gel. Elution with 60:40 hexanes:ethyl acetate yielded **15** (1.6 g, 3.0 mmol, 61%) as colorless gum. ^1^H NMR (400 MHz, CDCl_3_) δ 5.09 (s, 1H), 4.91 (d, *J* = 3.2 Hz, 1H), 4.56 (s, 1H), 2.39 (ddd, *J* = 15.3, 9.9, 5.0 Hz, 1H), 2.25 (ddd, *J* = 16.0, 9.6, 6.8 Hz, 1H), 2.14 (s, 3H), 2.11–2.02 (m, 8H), 2.02–1.91 (m, 2H), 1.88 (dd, *J* = 11.9, 7.4 Hz, 2H), 1.85–1.53 (m, 8H), 1.53–1.22 (m, 8H), 1.18–0.99 (m, 2H), 0.91 (s, 3H), 0.83 (d, *J* = 6.5 Hz, 3H), 0.73 (s, 3H).

(3*R*,5*S*,7*R*,8*R*,9*S*,10*S*,12*S*,13*R*,14*S*,17*R*)-17-((*R*)-but-3-en-2-yl)-10,13-dimethylhexadecahydro-1H-cyclopenta[a]phenanthrene-3,7,12-triyl-2,2,4,4-d_4_ triacetate (**14**). Triacetyl cholic acid-d4, **13** (1.6 g, 1 Eq, 3.0 mmol) in anhydrous benzene (63 mL) was refluxed with pyridine (0.61 g, 0.62 mL, 2.6 Eq, 7.7 mmol), copper (II) acetate (0.47 g, 0.88 Eq, 2.6 mmol) and lead tetraacetate (11 g, 8.4 Eq, 25 mmol) under argon for 2 h. The reaction mixture was cooled, and 1N HCl was added to precipitate the excess lead. The salts were filtered and washed with benzene, and the combined filtrates were washed with water and 5% NaOH (aq) to remove unreacted starting material. The organic layer was dried over MagSO4, filtered, and evaporated into a paste. The residue was chromatographed over flash silica gel. Elution with 25% ethyl acetate in hexanes yielded **14** (0.5 g, 1 mmol, 30%) as colorless foam. ^1^H NMR (400 MHz, CDCl_3_) δ 5.70–5.56 (m, 1H), 5.09 (s, 1H), 4.95–4.86 (m, 2H), 4.83 (dd, *J* = 10.1, 1.9 Hz, 1H), 4.56 (s, 1H), 2.15 (s, 3H), 2.06 (d, *J* = 11.7 Hz, 7H), 2.00–1.82 (m, 2H), 1.81–1.59 (m, 6H), 1.58 (d, *J* = 10.2 Hz, 1H), 1.58–1.47 (m, 1H), 1.48 (d, *J* = 4.7 Hz, 1H), 1.40 (dd, *J* = 12.2, 8.0 Hz, 1H), 1.32–1.22 (m, 1H), 1.16–1.01 (m, 2H), 0.95–0.89 (m, 6H), 0.75 (s, 3H). ^13^C NMR (101 MHz, CDCl_3_) δ 170.50, 170.31, 144.39, 112.17, 77.19, 75.27, 75.25, 73.89, 70.72, 47.19, 45.03, 43.42, 40.79, 40.30, 37.76, 34.46, 34.31, 31.21, 29.01, 27.32, 25.62, 22.81, 22.55, 21.56, 21.46, 21.41, 19.55, 12.46.

24*R*,25*S*-bisbenzyl aglycone-2,2,4,4-d_4_ (**15**). Starting from alkene **14**, aglycone **15** was synthesized in a similar manner as **10a**–**d**.

2,2,4,4-d_4_-27-nor-5β-cholestane-3α,7α,12α,24*R*,25*S* pentol (**16**). Starting from crude protected aglycone **15**, aglycone **16** was synthesized in a similar manner as **2a**. ^1^H NMR (400 MHz, CD_3_OD) δ 3.96 (t, *J* = 2.9 Hz, 1H), 3.79 (q, *J* = 3.0 Hz, 1H), 3.54 (qd, *J* = 6.3, 5.4 Hz, 1H), 3.34 (d, *J* = 1.5 Hz, 1H), 2.24 (td, *J* = 11.5, 6.5 Hz, 1H), 2.02–1.68 (m, 6H), 1.60–1.25 (m, 11H), 1.15 (d, *J* = 6.4 Hz, 3H), 1.14–1.05 (m, 1H), 1.02 (d, *J* = 6.4 Hz, 3H), 0.96 (d, *J* = 14.3 Hz, 1H), 0.91 (s, 3H), 0.72 (s, 3H). ^13^C NMR (101 MHz, CD_3_OD) δ 75.64, 72.69, 71.24, 70.48, 67.66, 46.04, 41.62, 41.57, 39.62, 35.66, 34.89, 34.44, 34.39, 31.88, 28.98, 28.17, 27.38, 26.48, 22.82, 21.73, 17.16, 16.58, 11.59. HRMS (ESI+, *m*/*z*): [M + Na]+ Calcd for C_26_H_42_D_4_O_5_Na [M + Na]^+^ 465.3594, found 465.3493

### 3.5. LCMS Methods

#### 3.5.1. Detection of 27-Nor-5β-Cholestane-3α,7α,12α,24,25 Pentol Glucuronide in Urine Samples

Liquid chromatography/mass spectrometry analysis was performed on a Waters Acquity UPLC^®^ coupled to a Waters Xevo Q-ToF quadruple time of flight mass spectrometer operating in electrospray ionization (ESI) in positive or negative mode. The capillary and sampling cone voltages were set to 3000 and 20 V, respectively. Source and desolvation temperatures were set to 120 °C and 450 °C, respectively, and the cone and desolvation gas flows were set to 50 and 800 L/hour, respectively. To maintain mass accuracy, leucine enkephalin was injected at a concentration of 200 pg/μL in 50% acetonitrile/water containing 0.1% formic acid at a rate of 10 μL/min. Data were acquired in continuum mode from 50 to 1000 *m*/*z*. The analytes were separated by reverse phase chromatography on an Acquity UPLC^®^ BEH C18 (1.7 μm, 2.1 × 50 mm) column. Chromatographic separation was achieved with water (A) and acetonitrile (B) each containing 0.1% formic acid. Gradient elution, with a flow rate of 0.500 mL/min, began with an initial hold of 2% B for 0.5 min, then increased to 20% B from 0.5 to 4.0 min, 20–95% B from 4.0 to 8.0 min, 95–100% B from 8.0–8.10 min, hold for 1 min at 100%, then return to initial conditions (2% B) in 0.10 min. The column temperature was maintained at 40 °C in a column oven. Identification was performed in either positive (pseudomolecular ions) or negative mode (Table 1). Because the glucuronides readily fragment in ESI+ mode (Figure 4), it is recommended to use ESI− mode for identification and quantitation.

#### 3.5.2. Resolution of C24,C25 Diastereomers of 27-Nor-5β-Cholestane-3α,7α,12α,24,25 Pentol in Urine Samples

##### Preparation of Urine Samples for Detection of 27-Nor-5β-Cholestane-3α,7α,12α,24,25 Pentol

50 μL of urine, 200 μL of 200 mM sodium acetate buffer (pH 3.8), and 5000 units of β-glucuronidase were incubated together for 4 h at 37 °C. The reaction was terminated by adding an equal volume of acetonitrile and centrifuged at 18,000× *g* for 20 min at 4 °C. A 20 μL sample was transferred to an LC/MS vial for analysis.

##### LC/MS Method for Resolution of C24,C25 Diastereomers 27-Nor-5β-Cholestane-3α,7α,12α,24,25 Pentol

Liquid chromatography/mass spectrometry analysis was performed on a Waters Acquity UPLC^®^ coupled to a Waters Xevo Q-ToF quadruple time of flight mass spectrometer operating in electrospray ionization (ESI) in positive mode. The capillary and sampling cone voltages were set to 3000 and 20 V, respectively. Source and desolvation temperatures were set to 120 °C and 450 °C, respectively, and the cone and desolvation gas flows were set to 50 and 800 L/hour, respectively. To maintain mass accuracy, leucine enkephalin was injected at a concentration of 200 pg/μL in 50% acetonitrile/water containing 0.1% formic acid at a rate of 10 μL/min. Data were acquired in profile mode from 50 to 1200 *m*/*z*. The analytes were separated by reverse phase chromatography on a Phenomenex Synergi-Hydro (2.5 μm, 2.0 × 100 mm) column. Chromatographic separation was achieved with water (A) and acetonitrile (B) each containing 0.1% formic acid. Gradient elution, with a flow rate of 0.400 mL/min, began with an initial hold of 40% B for 0.3 min, increased to 50%B over 6 min, held for 0.65 min at 95%, then returned to initial conditions (40%B) in 0.10 min. The column temperature was maintained at 40 °C in a column oven. Due to significant amounts of insource fragmentation, analytes were identified using pseudomolecular ions *m*/*z* 385.3+, 403.3+, and 461.3+ (M + Na) and retention time.

## 4. Conclusions

An untargeted metabolomic study identified four features, including “561+”, as potential biomarkers for the early detection of lung cancer in urine. We isolated “561+” from human urine and tentatively identified it as 27-nor-5β-cholestane-3α,7α,12α,24,25 pentol glucuronide of unknown C24,25 stereochemistry using ^1^H NMR and mass spectrometry. The four diastereomeric aglycones **2a**–**d** were synthesized and resolved using LC/MS to determine the C24,25 stereochemistry. The treatment of human urine from lung cancer patients containing 27-nor-5β-cholestane-3α,7α,12α,24,25 pentol glucuronide with β-glucuronidase released an aglycone with the 24*R*,25*S* stereochemistry and a small amount of 24*S*,25*S*, which is opposite to the reported C24,C25 stereochemistry in normal patients. We coupled 24*R*,25*S* aglycone to the glucuronide to complete the first reported synthesis of 27-nor-5β-cholestane-3α,7α,12α,24*R*,25*S* pentol glucuronide (**1a**). The structure of “561+” was confirmed as **1a** by LCMS. The d_4_-labeled aglycone **16** was also prepared to assist with mass spectrometry quantitation.

Our C24,25 assignments are opposite to those reported by Une and coworkers [12]. However, the identification of the C24 position as *R* agrees with the reported C24 stereochemistry of the structurally related bile alcohol, 5*β*-Ranol, the major bile constituent of the bullfrog *Rana catesbeina* [29]. The biochemical origin of 27-nor-5β-cholestane-3α,7α,12α,24,25 pentol is unknown but mostly likely involves the alternative or aberrant pathway for the biosynthesis of bile acids [30]. In this pathway, Dayal [31] has shown that 3α,7α,12α,25-cholestane-5β-tetrol is converted to a mixture of 24*R* and 24*S* 3α,7α,12α, 24,25-cholestane-5β-tetrols, with the 24*R* isomer being predominant. Only the 24*R* tetrol is converted to cholic acid, resulting in the detection of 3α,7α,12α, 24*S*,25-cholestane-5β-tetrol in the urine and bile of patients with cerebrotendinous xanthomatosis (CTX) disease [31]. It is possible that metabolic changes that occur with lung cancer prevent the conversion of 27-nor-5β-cholestane-3α,7α,12α,24*R*,25*S* pentol to cholic acid, causing it to be excreted as glucuronide. Additional studies are needed to understand the biochemical pathways involved.

## Figures and Tables

**Table 1:** ESI+ and ESI− ions used for identification and quantitation of 27-nor-5β-Cholestane-3α,7α,12α,24,25 pentol glucuronide.

Compound	Molecular Formula	Exact Mass	Positive Quantitation Ion(s) (Pseudomolecular Ions)	Negative Quantitation Ion, M-H
27-nor-5β-Cholestane-3α,7α,12α,24,25 pentol glucuronide	C_32_H_54_O_11_	614.3666	385.3, 403.3, 561.3, 579.3, 597.3	613.3
27-nor-5β-Cholestane-3α,7α,12α,24,25 pentol glucuronide-2,2,4,4-d_4_	C_32_H_50_D_4_O_11_	618.3917	389.3, 407.3, 565.3, 583.3, 601.3	617.3

## Data Availability

The original contributions presented in the study are included in the article/supplementary material, further inquiries can be directed to the corresponding authors. In addition, they are openly available in Figshare (https://figshare.com): DOI: 10.25444/nhlbi.25733652.

## Supplementary Materials

The following supporting information can be downloaded at: https://www.mdpi.com/article/10.3390/molecules29122781/s1, ^1^H and ^13^C spectra for prepared compounds.
