# Supplementary material for: Isolation and Identification of a Urinary Biomarker for Lung Cancer: 27-Nor-5β-Cholestane-3α,7α,12α,24R,25S Pentol Glucuronide and Its Deuterated Analog
Source: Molecules. 2024 Jun 11;29(12):2781. doi: 10.3390/molecules29122781 (PMC11206657; doi:10.3390/molecules29122781)

Isolation and Identification of a Urinary Biomarker for Lung Cancer: 27-Nor-5β-cholestane-3α,7α,12α,24R,25S pentol glucuronide and its Deuterated Analog

Burchelle Blackman, Natarajan Raju, Chandrasekhar Mushti, Kelly Lane, Daxeshkumar Patel, Curtis Harris, and Rolf E. Swenson

Supplemental Information

MS Spectrum of "561+" isolated from human urine

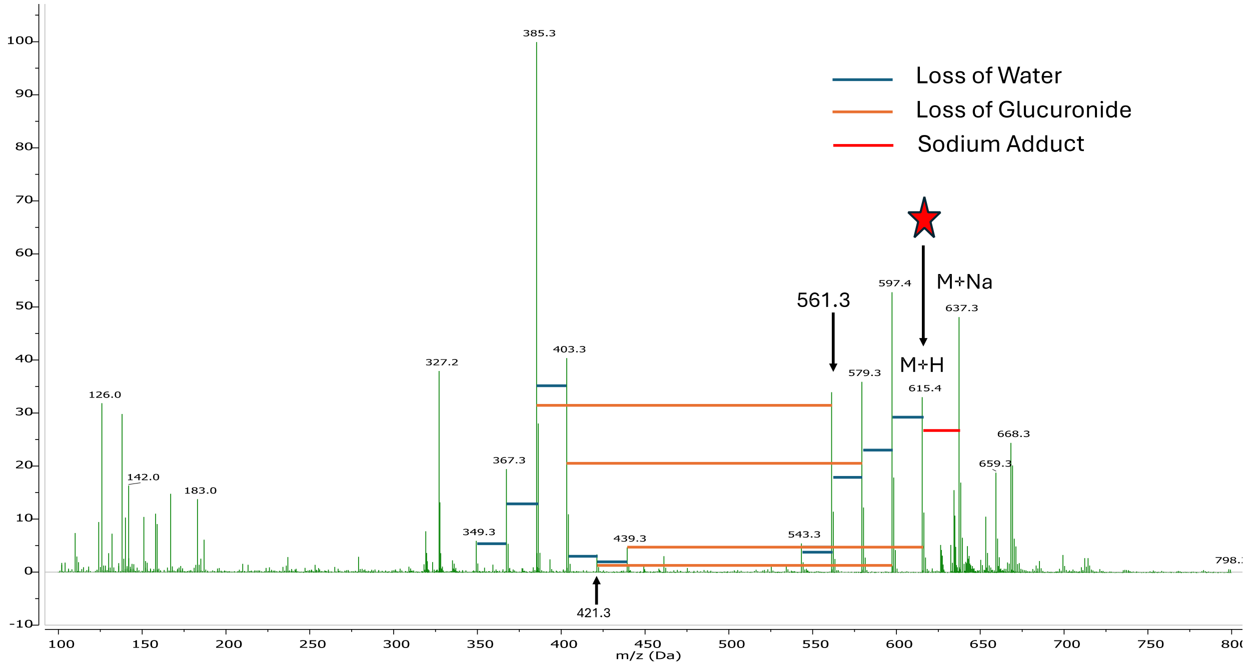

Proton and Carbon NMR Spectra

Compound 1a

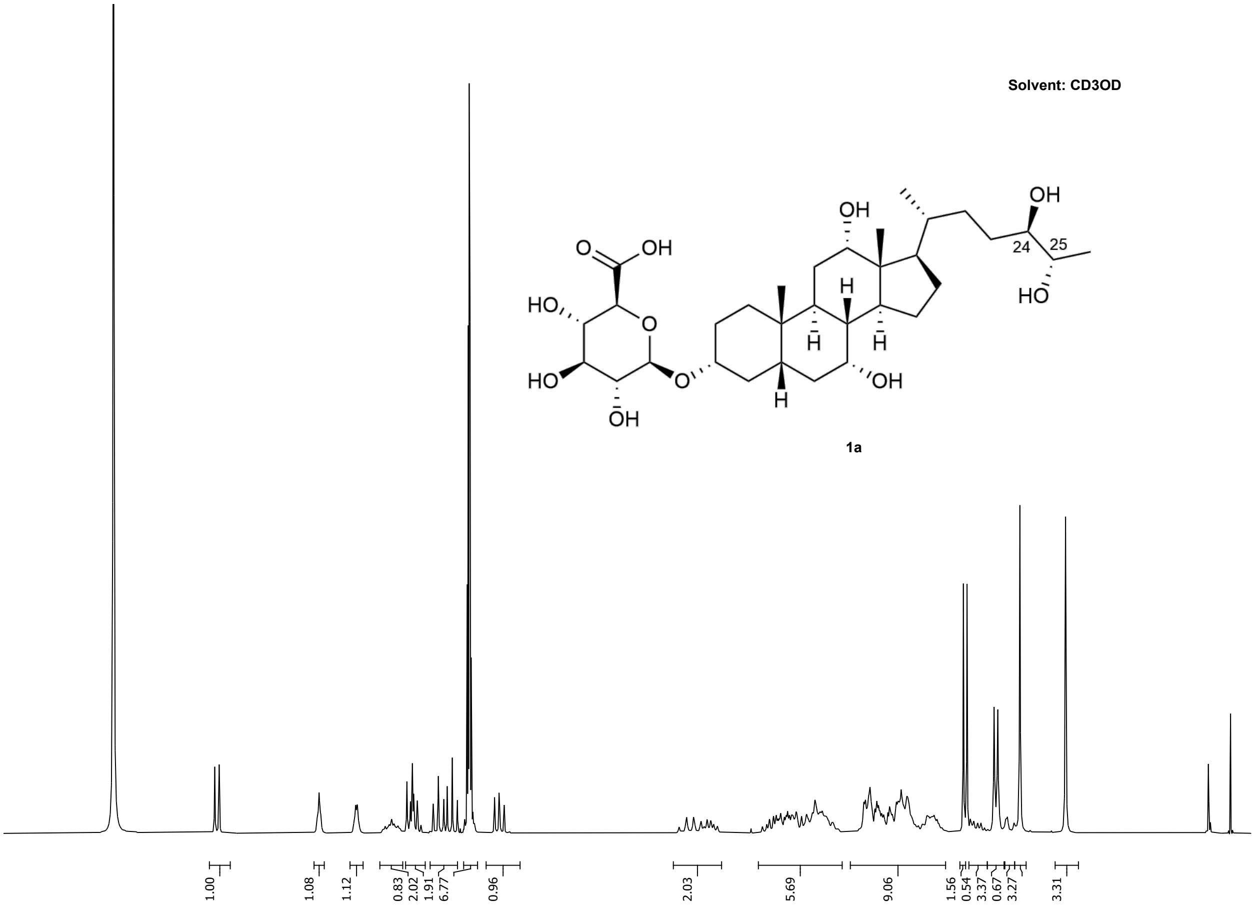

Solvent: CD3OD

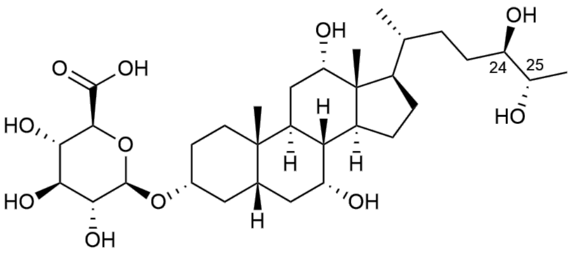

1a

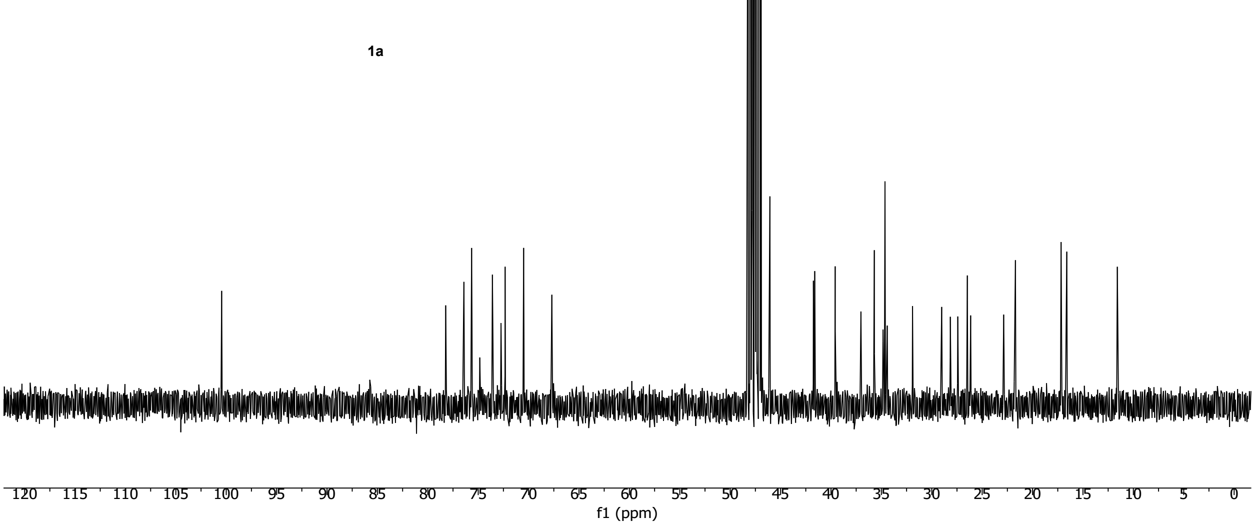

Compound 2a

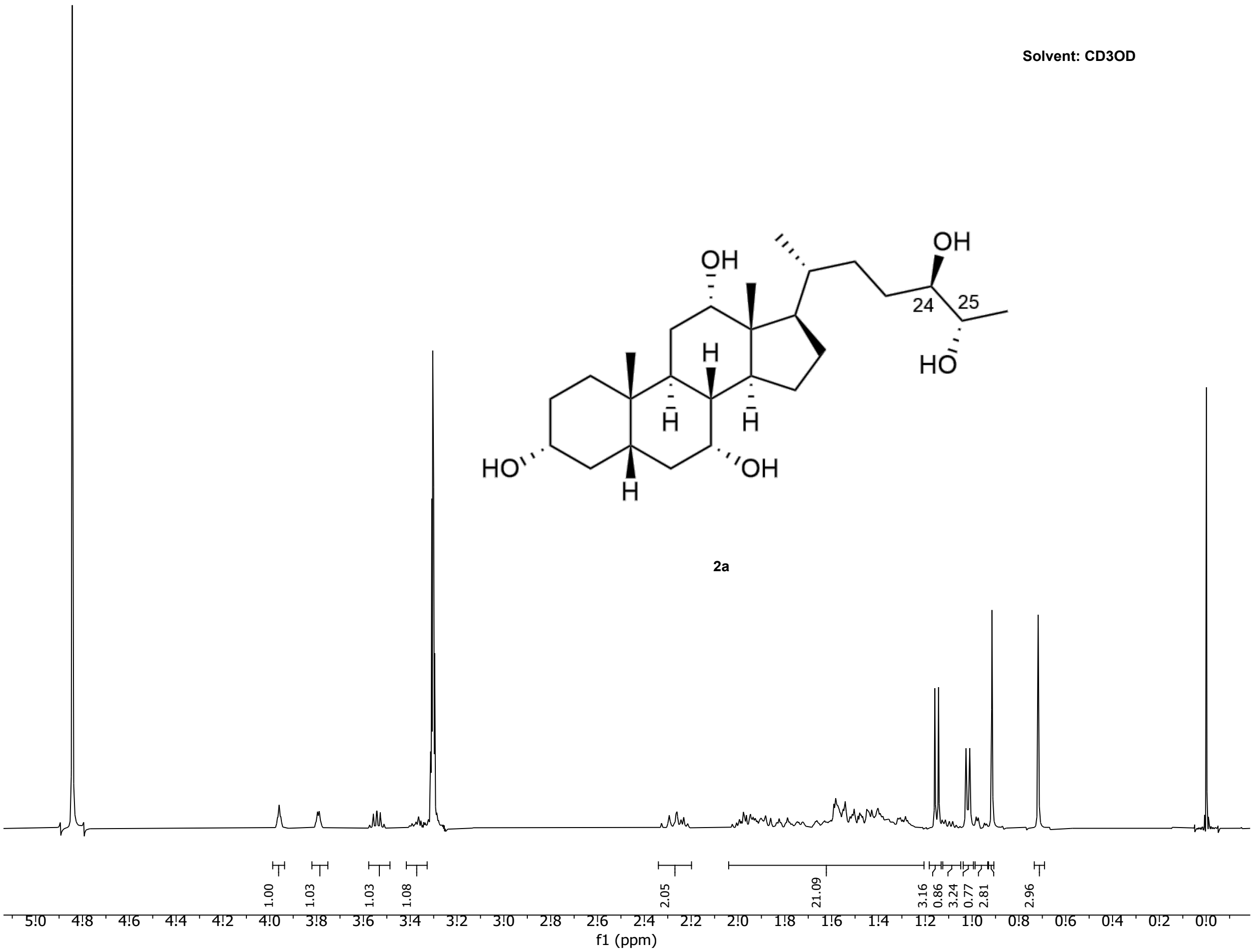

Solvent: CD3OD
OH
OH
24
25
HO
H
H
H
HO
OH
H
2a
1.00
1.03
1.03
1.08
2.05
21.09
3.16
0.86
3.24
0.77
2.81
2.96
5.0
4.8
4.6
4.4
4.2
4.0
3.8
3.6
3.4
3.2
3.0
2.8
2.6
2.4
2.2
2.0
1.8
1.6
1.4
1.2
1.0
0.8
0.6
0.4
0.2
0.0
f1 (ppm)

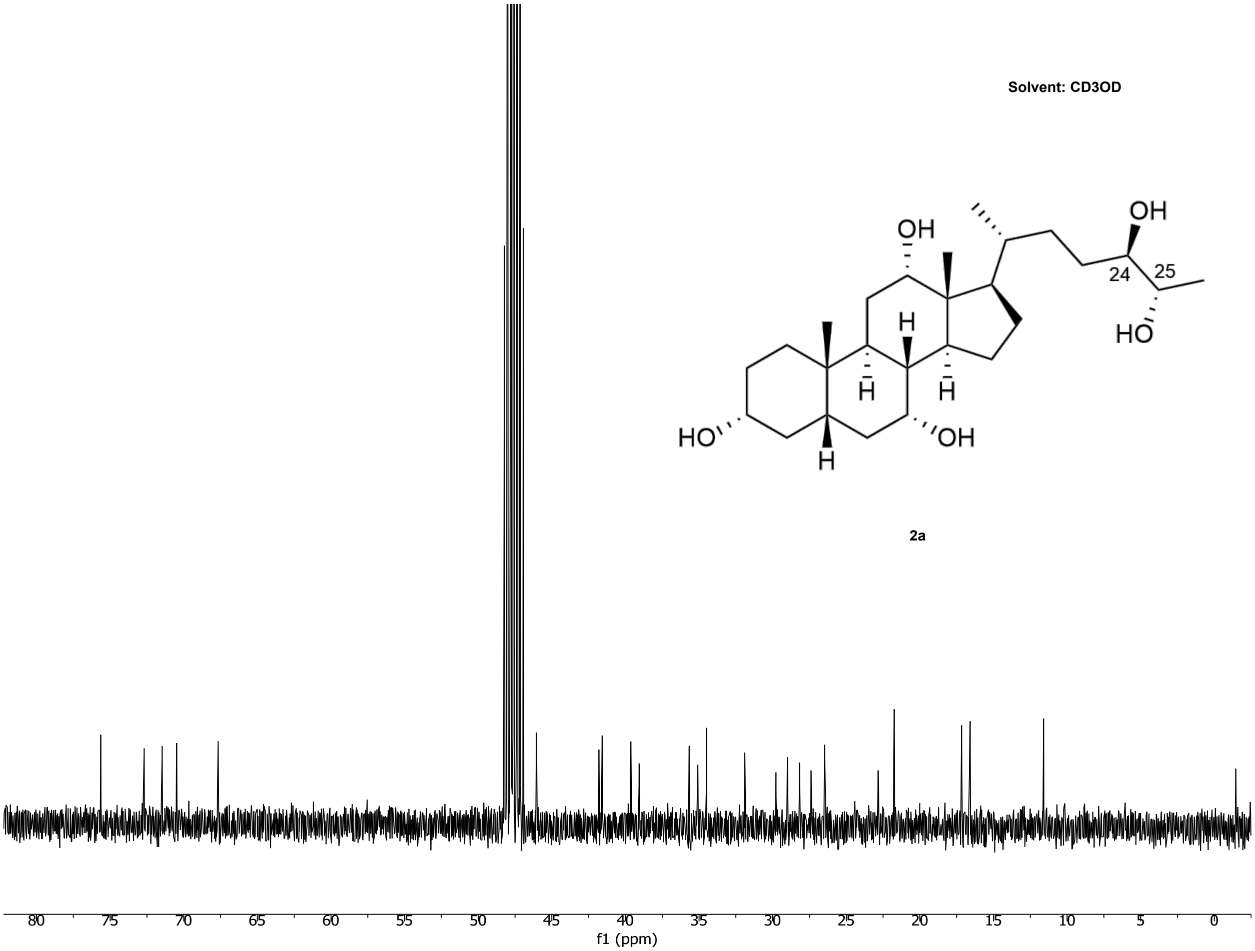

Solvent: CD3OD
OH
OH
24
25
HO
H
H
H
HO
OH
H
2a
80
75
70
65
60
55
50
45
40
35
30
25
20
15
10
5
0
f1 (ppm)

Compound 2b

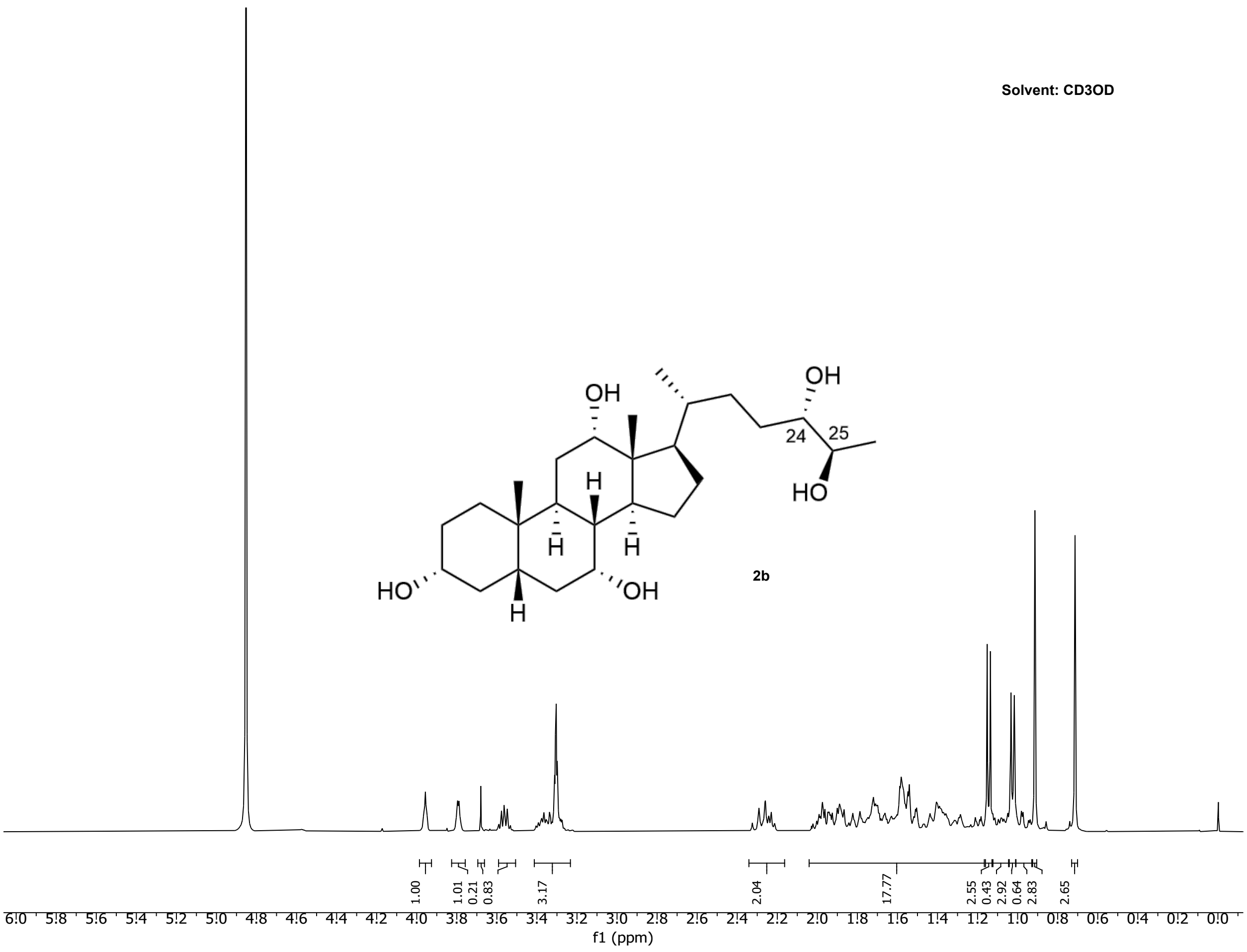
Solvent: CD3OD
OH
OH
24
25
HO
H
H
H
HO
H
OH
2b
1.00
1.01
0.21
0.83
3.17
2.04
17.77
2.55
0.43
2.92
0.64
2.83
2.65
6.0 5.8 5.6 5.4 5.2 5.0 4.8 4.6 4.4 4.2 4.0 3.8 3.6 3.4 3.2 3.0 2.8 2.6 2.4 2.2 2.0 1.8 1.6 1.4 1.2 1.0 0.8 0.6 0.4 0.2 0.0
f1 (ppm)

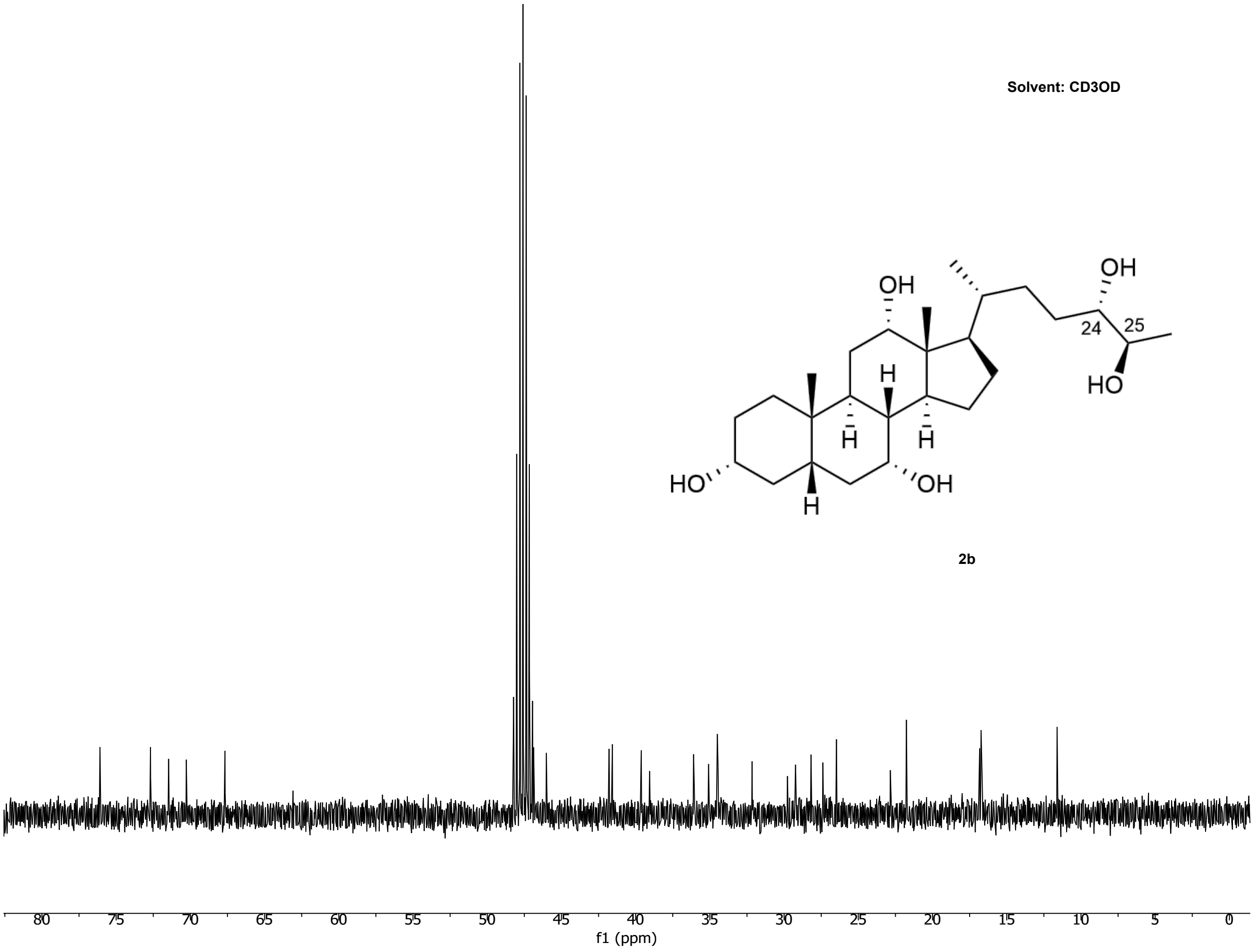
Solvent: CD3OD
OH
OH
24
25
HO
H
H
H
HO
OH
H
2b
80 75 70 65 60 55 50 45 40 35 30 25 20 15 10 5 0
f1 (ppm)

Compound 2c

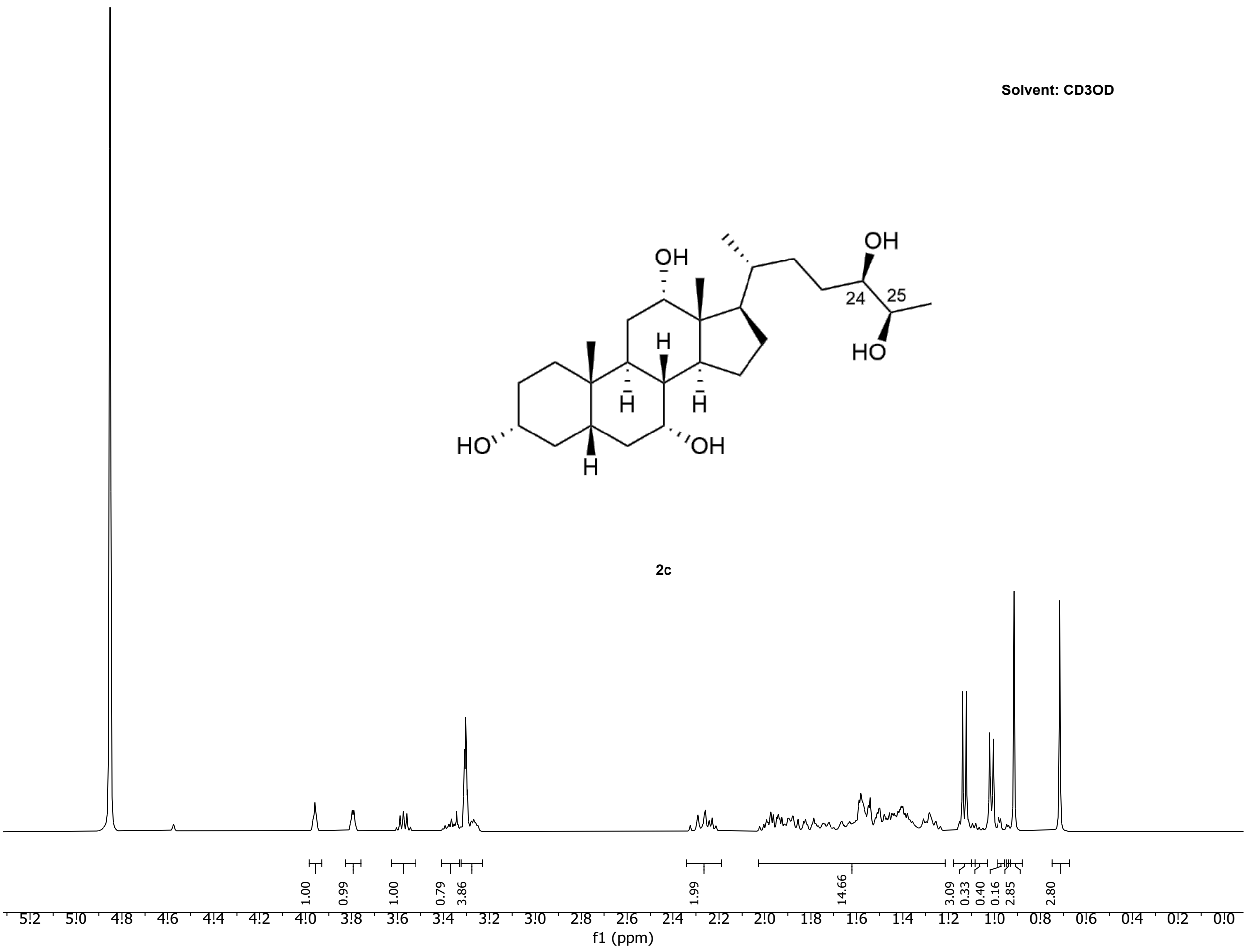

Solvent: CD3OD
OH
OH
24
25
HO
H
H
H
HO
H
OH
2c
1.00
0.99
1.00
0.79
3.86
1.99
14.66
3.09
0.33
0.40
0.16
2.85
2.80
5.2
5.0
4.8
4.6
4.4
4.2
4.0
3.8
3.6
3.4
3.2
3.0
2.8
2.6
2.4
2.2
2.0
1.8
1.6
1.4
1.2
1.0
0.8
0.6
0.4
0.2
0.0
f1 (ppm)

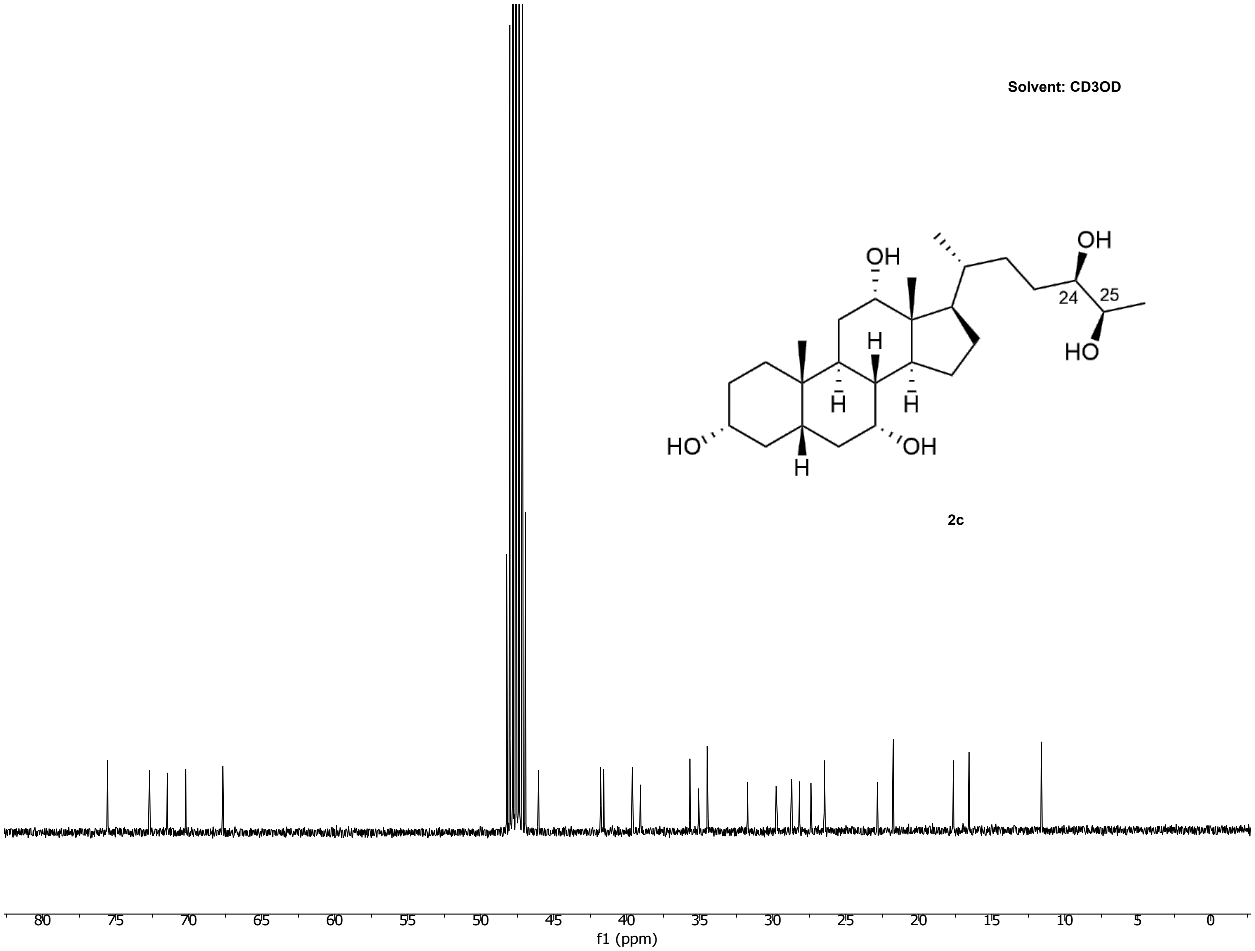
Solvent: CD3OD
OH
OH
HO
OH
HO
H
H
H
H
24
25
80
75
70
65
60
55
50
45
40
35
30
25
20
15
10
5
0
f1 (ppm)

2c

Compound 2d

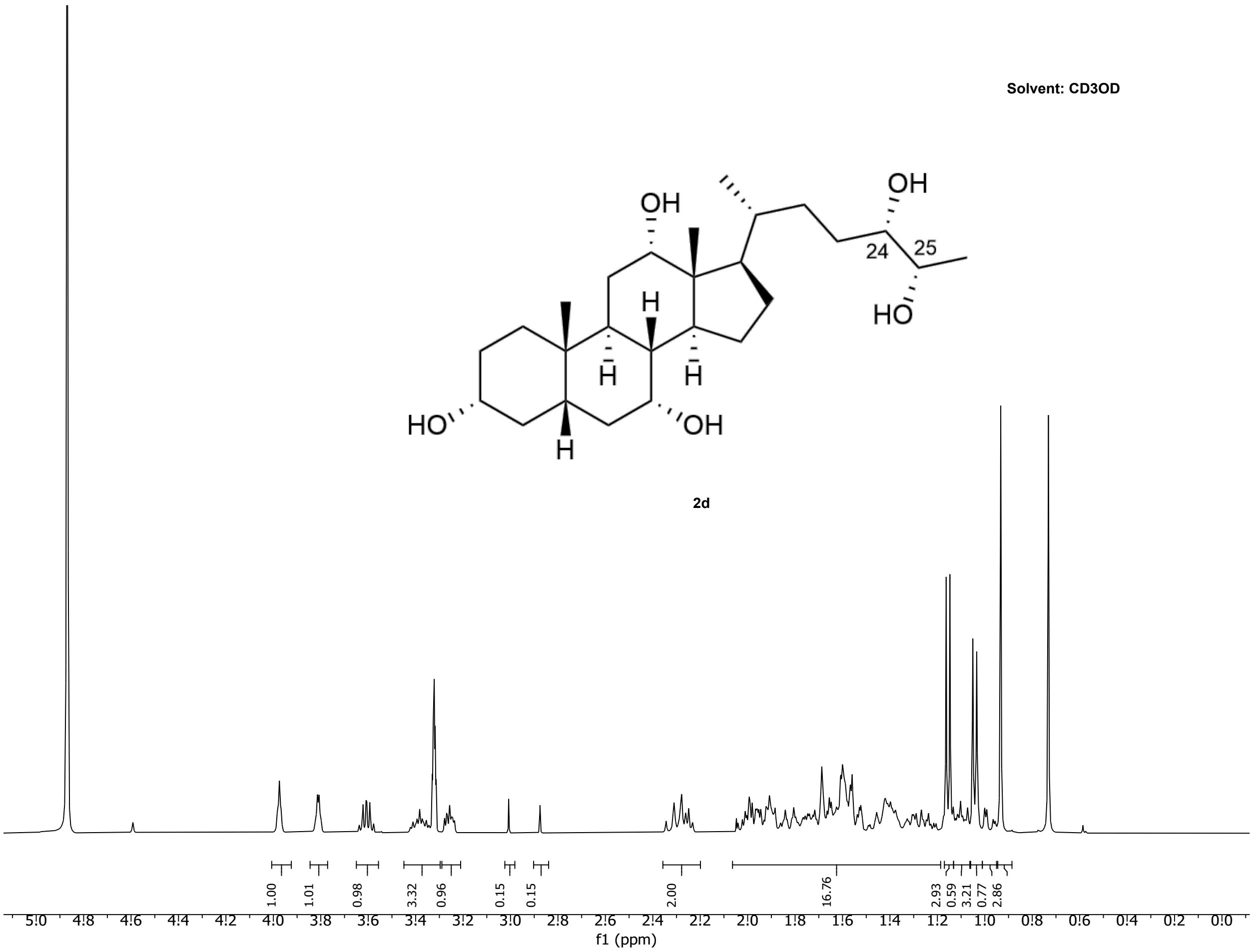
Solvent: CD3OD
OH
OH
24
25
HO
H
H
H
HO
OH
H
2d
1.00
1.01
0.98
3.32
0.96
0.15
0.15
2.00
16.76
2.93
0.59
3.21
0.77
2.86
5.0 4.8 4.6 4.4 4.2 4.0 3.8 3.6 3.4 3.2 3.0 2.8 2.6 2.4 2.2 2.0 1.8 1.6 1.4 1.2 1.0 0.8 0.6 0.4 0.2 0.0
f1 (ppm)

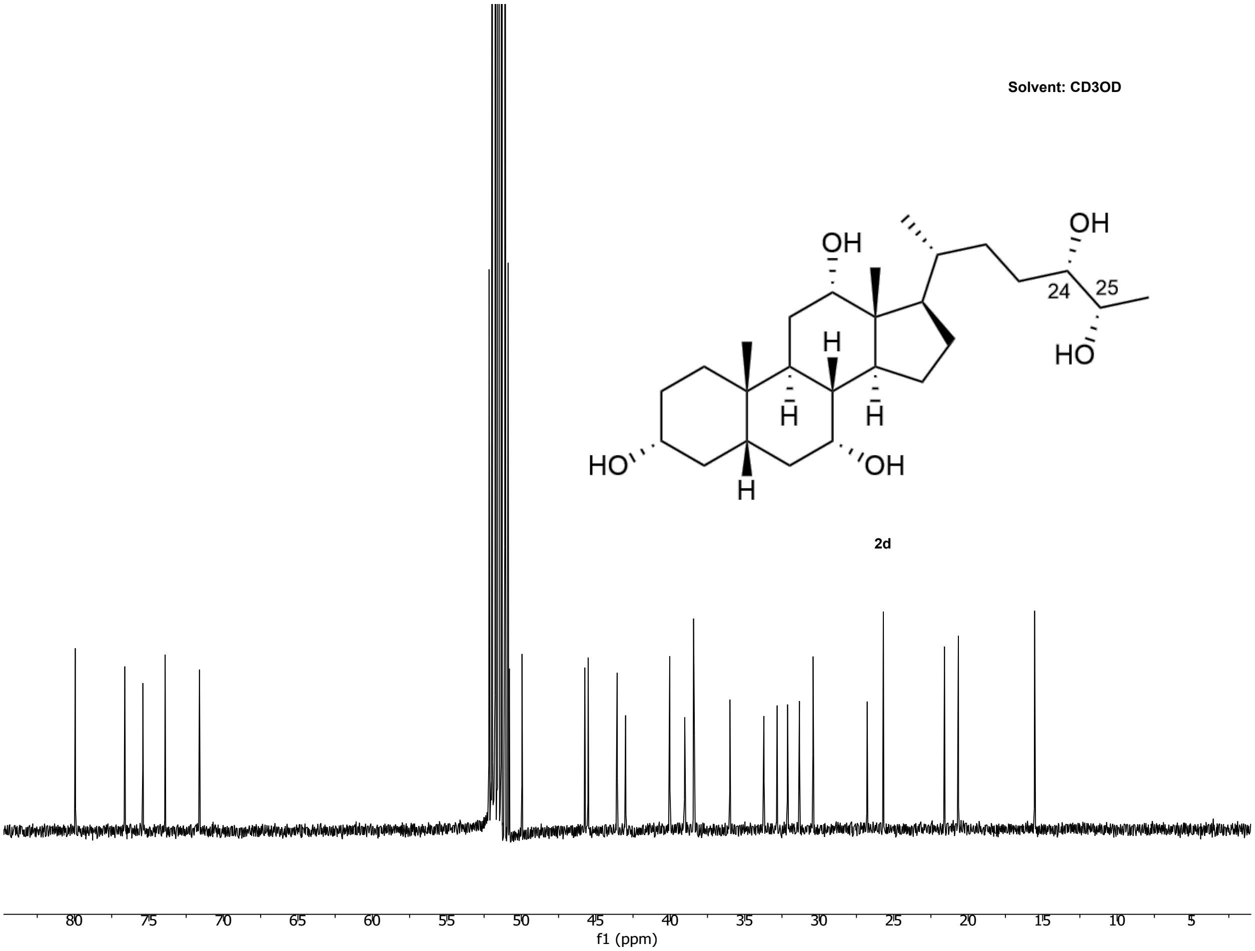

Solvent: CD3OD
OH
OH
24
25
HO
H
H
H
HO
OH
H
2d
80
75
70
65
60
55
50
45
40
35
30
25
20
15
10
5
f1 (ppm)

Compound 5a

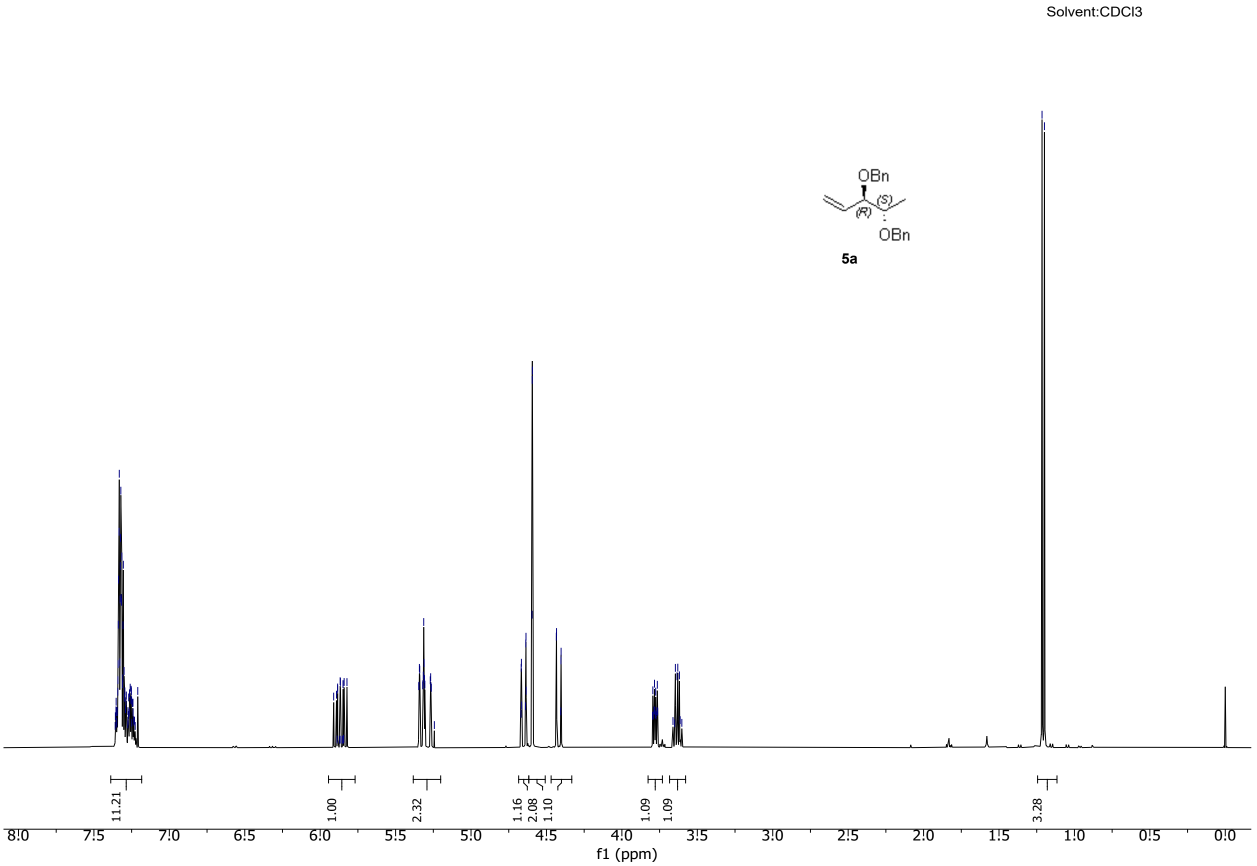

Solvent:CDCl3
OBn
(R)
(S)
OBn
5a
11.21
1.00
2.32
1.16
2.08
1.10
1.09
1.09
3.28
8.0
7.5
7.0
6.5
6.0
5.5
5.0
4.5
4.0
3.5
3.0
2.5
2.0
1.5
1.0
0.5
0.0
f1 (ppm)

Solvent: CDCl3

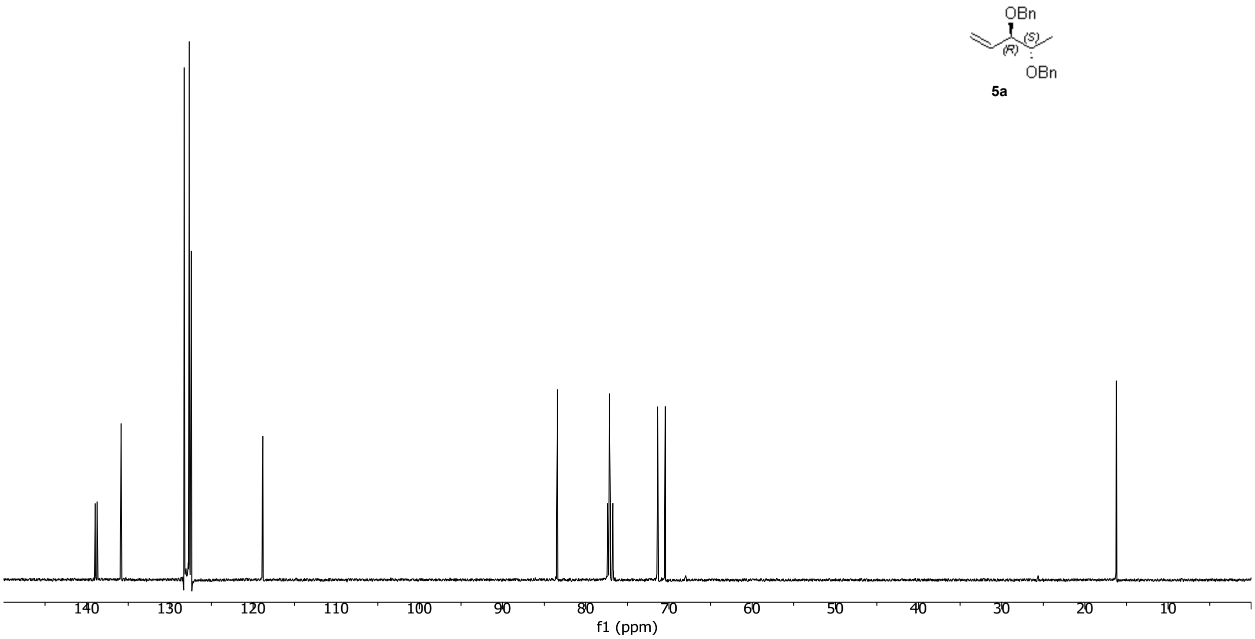

Compound 5b

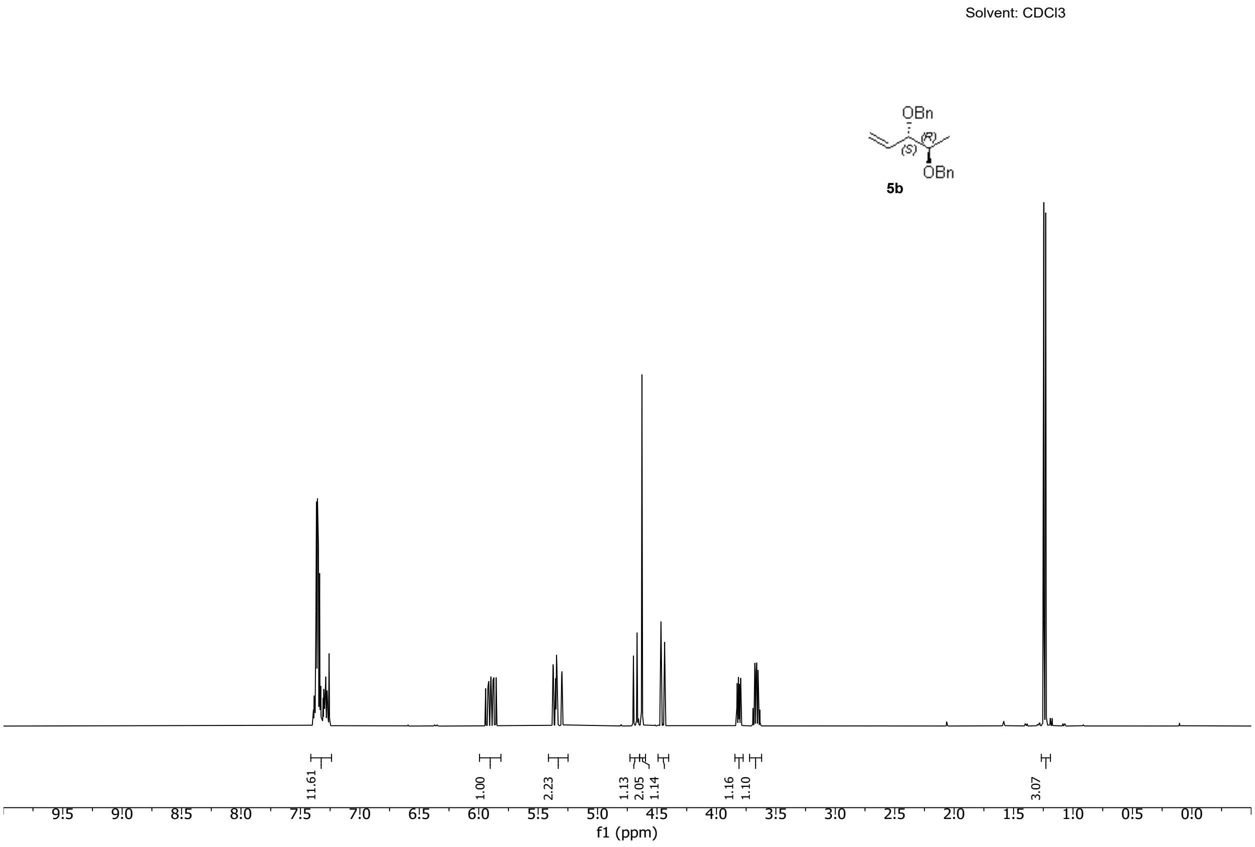
Solvent: CDCl3
OBn
(S)
(R)
OBn
5b
11.61
1.00
2.23
1.13
2.05
1.14
1.16
1.10
3.07
9.5
9.0
8.5
8.0
7.5
7.0
6.5
6.0
5.5
5.0
4.5
4.0
3.5
3.0
2.5
2.0
1.5
1.0
0.5
0.0
f1 (ppm)

Solvent: CDCl3

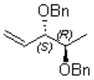

5b

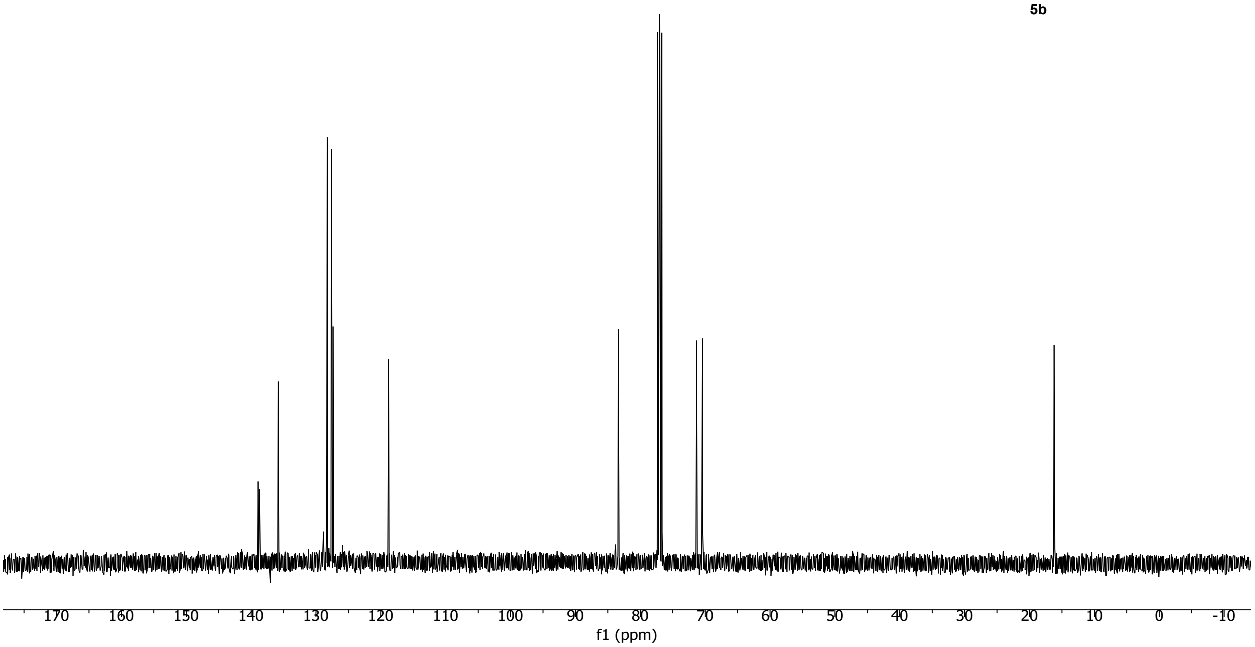

Compound 5c

Solvent: CDCl3

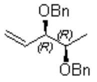

5c

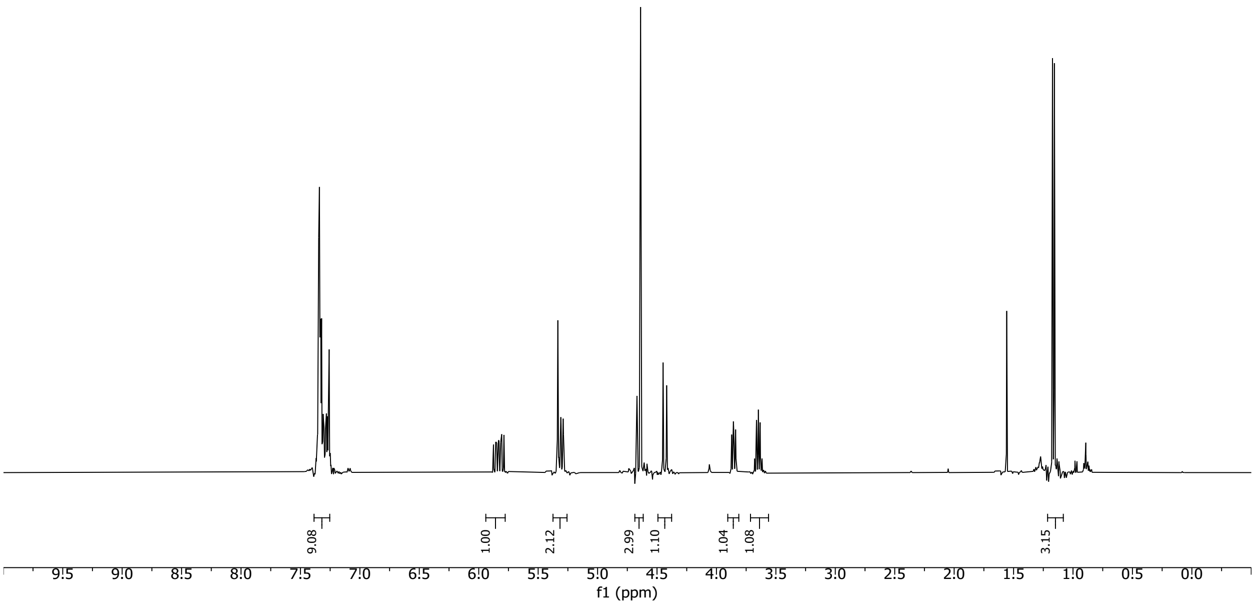

Solvent CDCl3

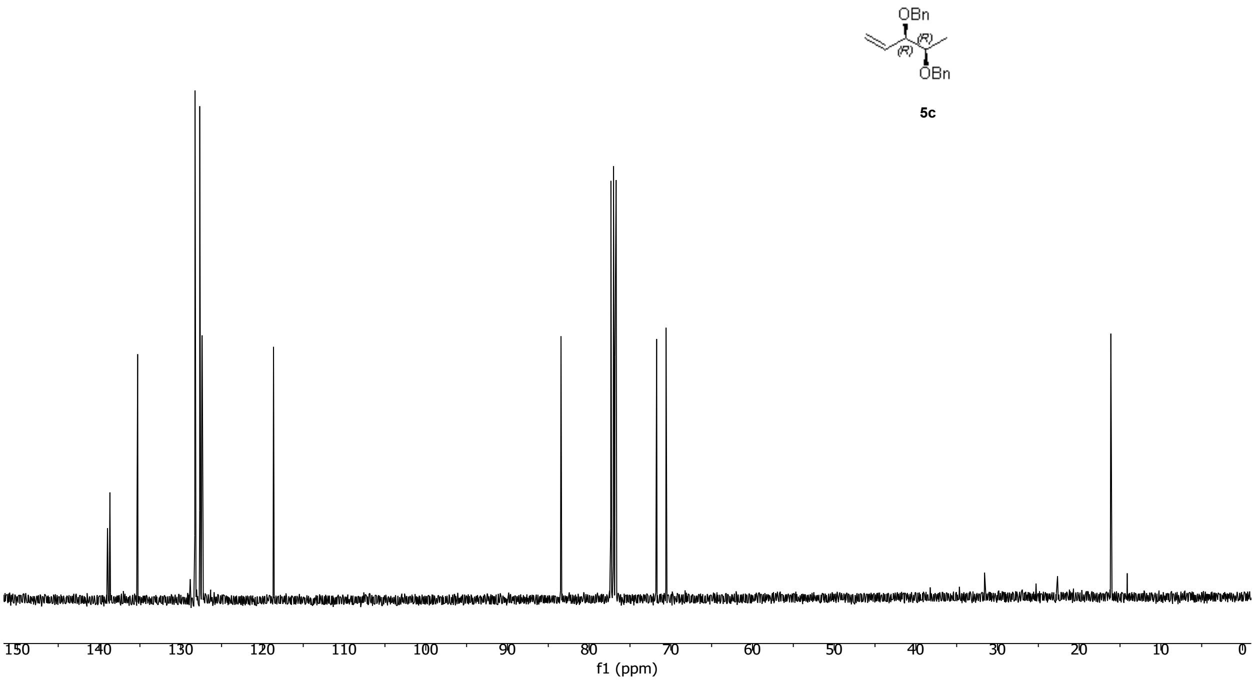

Compound 5d

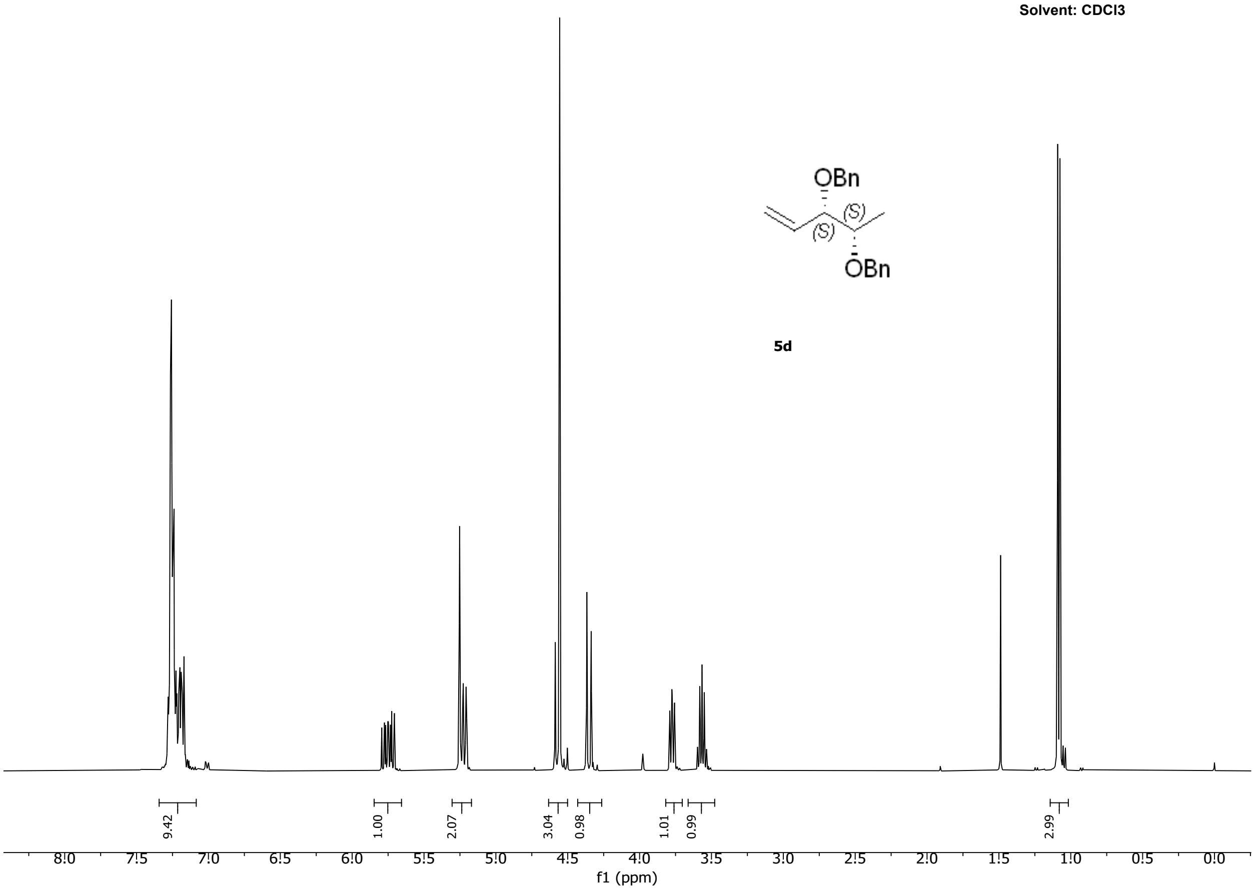
Solvent: CDCl3
OBn
(S)
(S)
OBn
5d
9.42
1.00
2.07
3.04
0.98
1.01
0.99
2.99
8.0
7.5
7.0
6.5
6.0
5.5
5.0
4.5
4.0
3.5
3.0
2.5
2.0
1.5
1.0
0.5
0.0
f1 (ppm)

Solvent: CDCl3

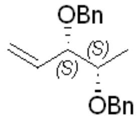

5d

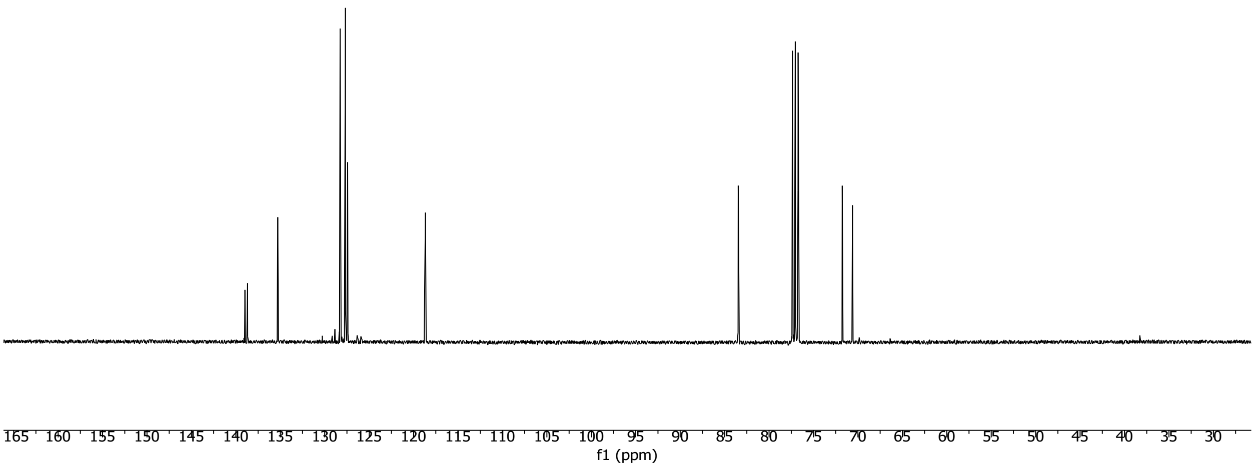

Compound 7a

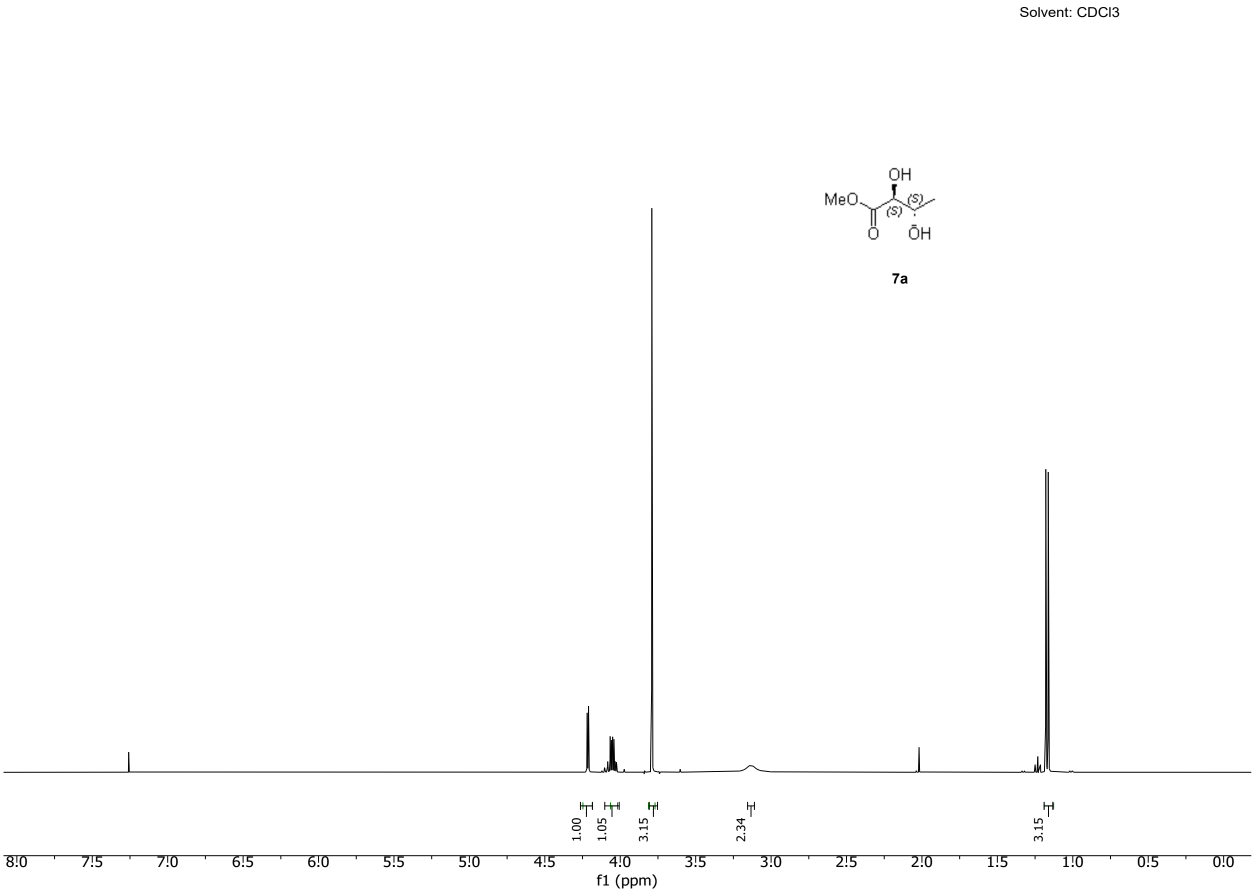
Solvent: CDCl3
MeO
OH
(S)
(S)
O
OH
7a
1.00
1.05
3.15
2.34
3.15
8.0
7.5
7.0
6.5
6.0
5.5
5.0
4.5
4.0
3.5
3.0
2.5
2.0
1.5
1.0
0.5
0.0
f1 (ppm)

Solvent: CDCl3

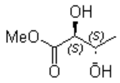

7a

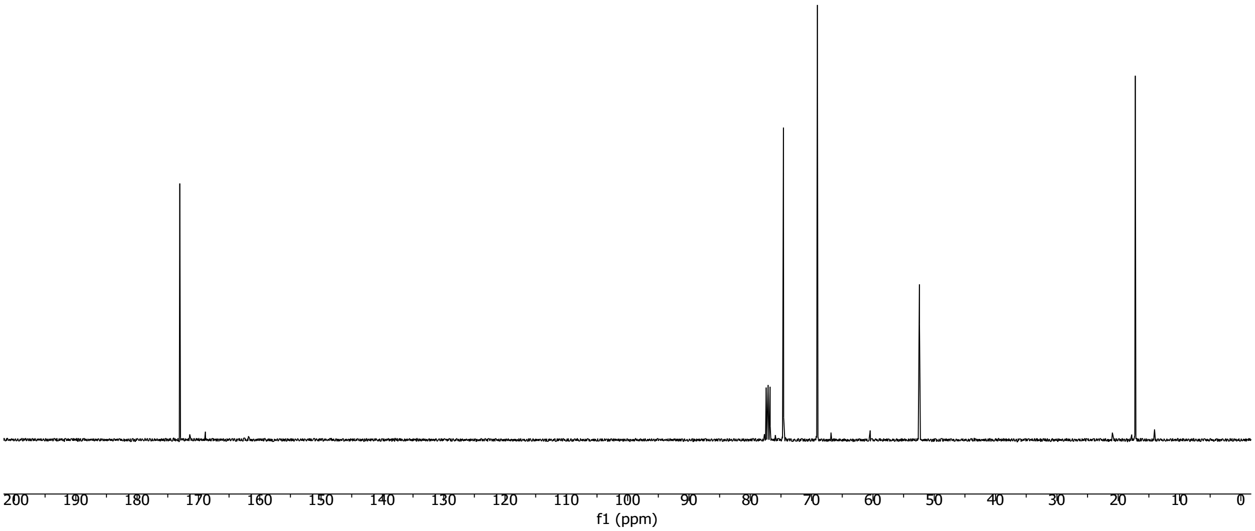

Compound 7b

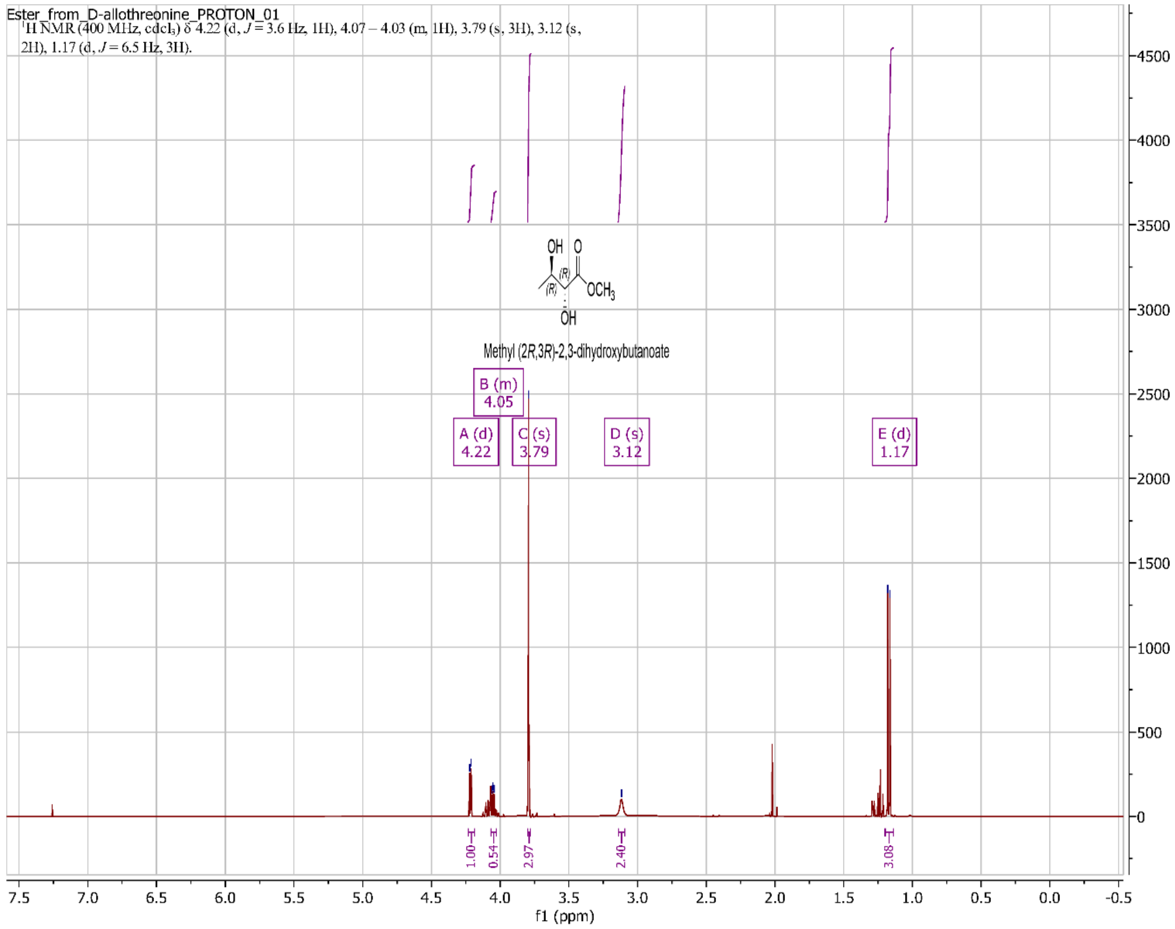

Ester_from_D-allothreonine_PROTON_01
1H NMR (400 MHz, cdcl3) δ 4.22 (d, J = 3.6 Hz, 1H), 4.07 – 4.03 (m, 1H), 3.79 (s, 3H), 3.12 (s, 2H), 1.17 (d, J = 6.5 Hz, 3H).
Methyl (2R,3R)-2,3-dihydroxybutanoate
B (m) 4.05
A (d) 4.22
C (s) 3.79
D (s) 3.12
E (d) 1.17
1.00
0.54
2.97
2.40
3.08
f1 (ppm)

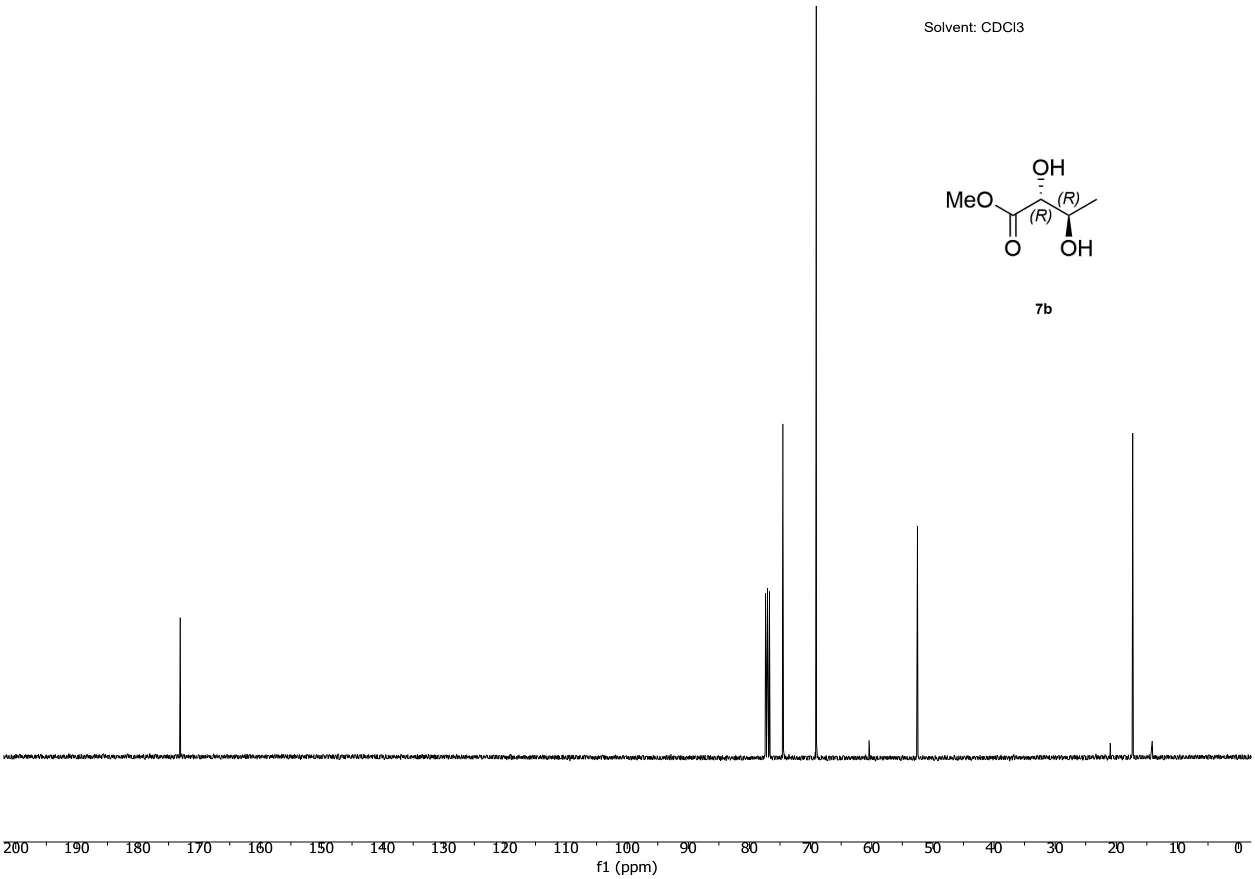
Solvent: CDCl3
OH
MeO
(R)
(R)
O
OH
200 190 180 170 160 150 140 130 120 110 100 90 80 70 60 50 40 30 20 10 0
f1 (ppm)

7b

Compound 7c

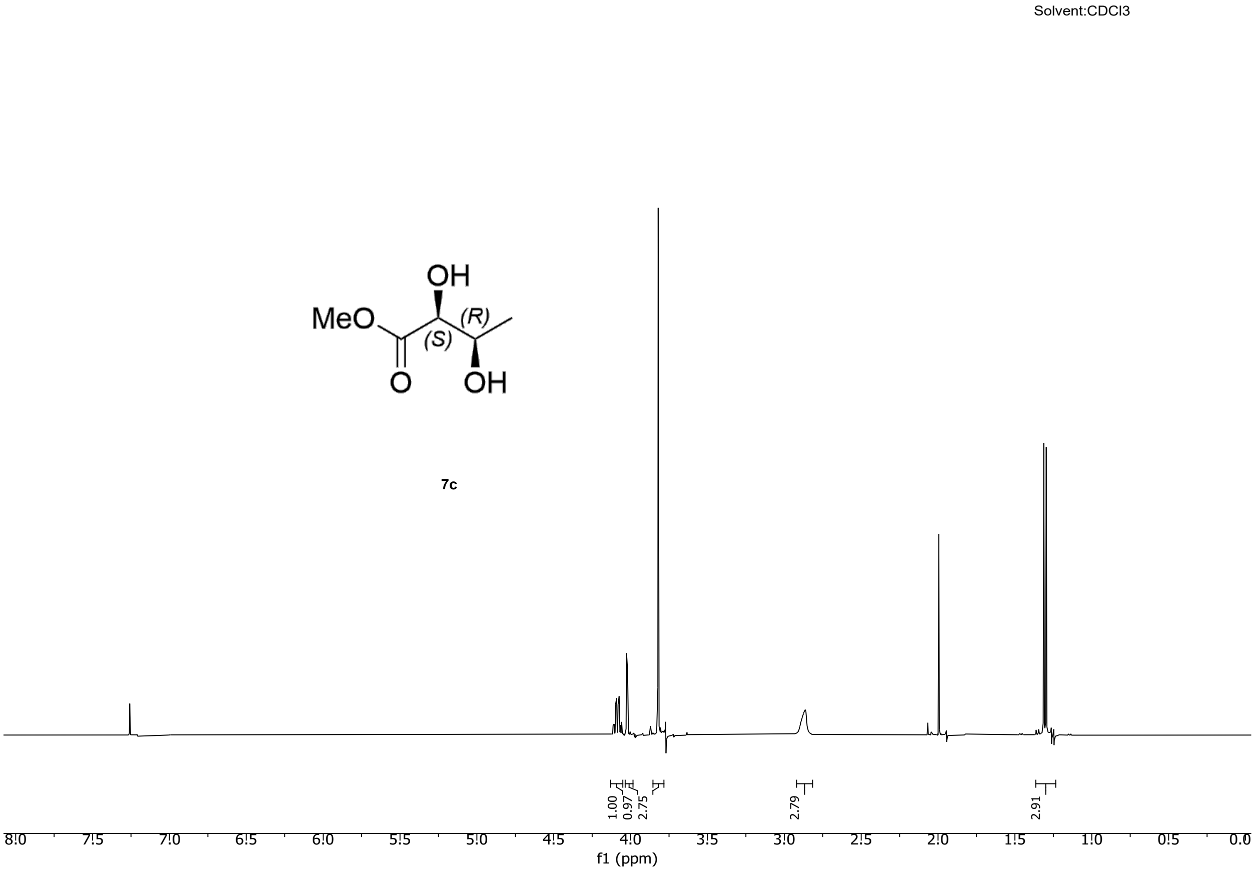

Solvent:CDCl3
MeO
OH
OH
O
(S)
(R)
7c
1.00
0.97
2.75
2.79
2.91
8.0
7.5
7.0
6.5
6.0
5.5
5.0
4.5
4.0
3.5
3.0
2.5
2.0
1.5
1.0
0.5
0.0
f1 (ppm)

Solvent:CDCl3

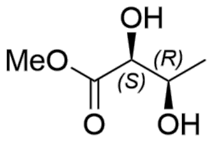

7c

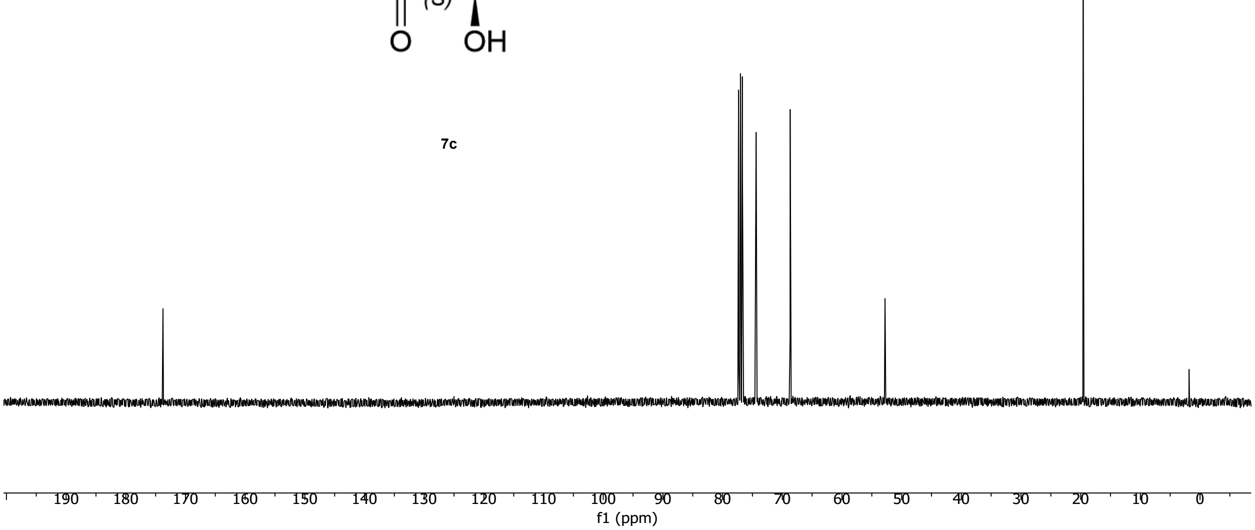

Compound 7d

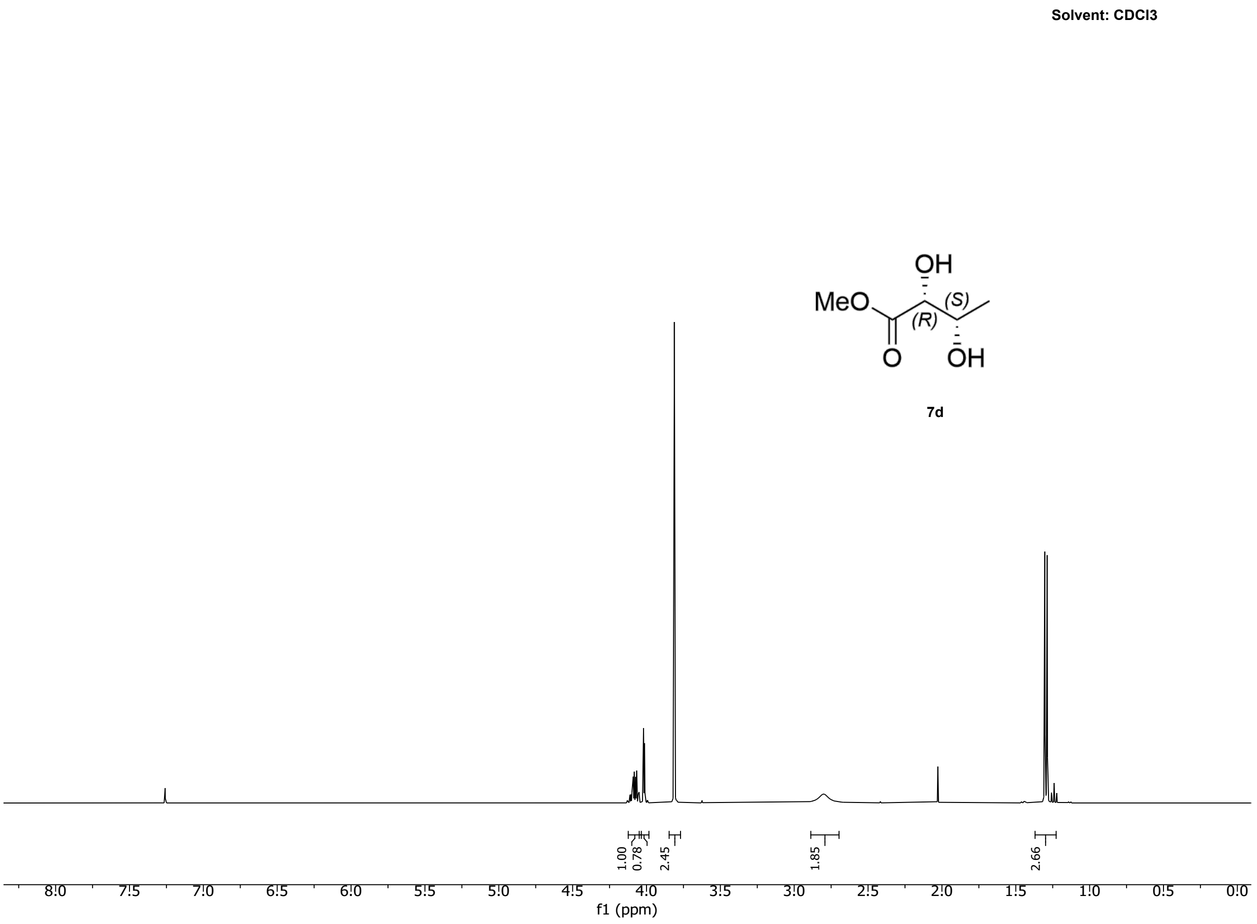

Solvent: CDCl3
MeO
OH
(R)
(S)
O
OH
7d
1.00
0.78
2.45
1.85
2.66
8.0
7.5
7.0
6.5
6.0
5.5
5.0
4.5
4.0
3.5
3.0
2.5
2.0
1.5
1.0
0.5
0.0
f1 (ppm)

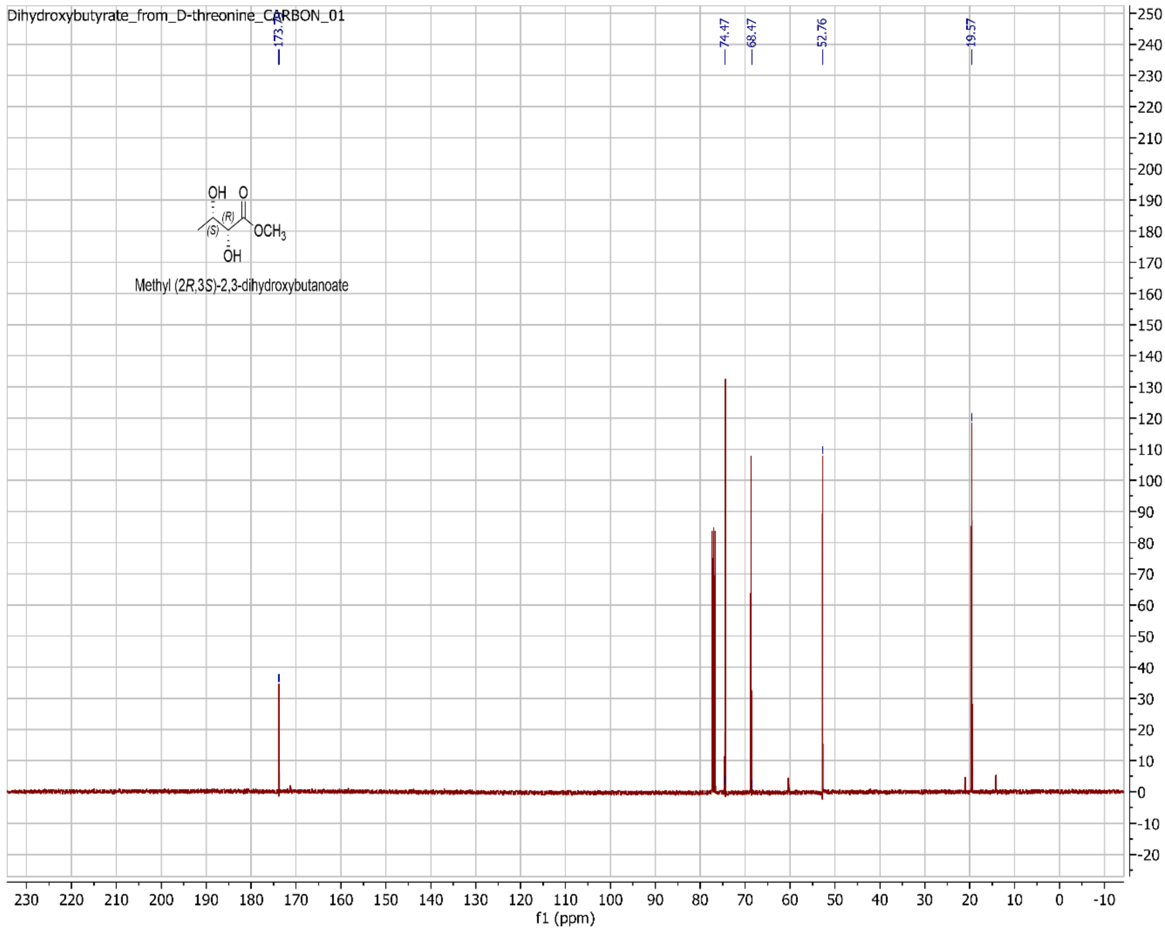

Dihydroxybutyrate_from_D-threonine_CARBON_01
173.7
74.47
68.47
52.76
19.57
OH
O
(R)
(S)
OCH3
OH
Methyl (2R,3S)-2,3-dihydroxybutanoate
f1 (ppm)

Compound 8a

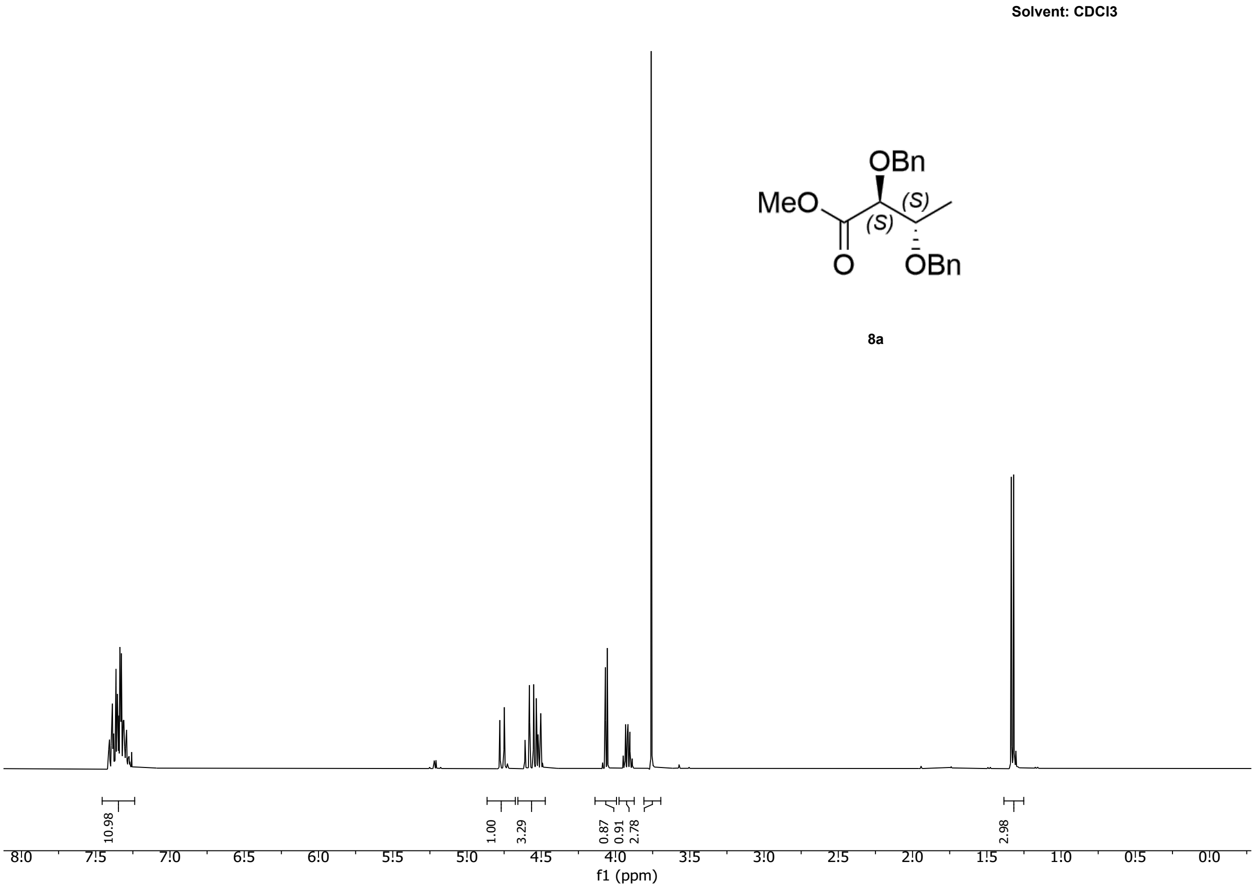

Solvent: CDCl3
OBn
MeO
(S)
(S)
O
OBn
8a
10.98
1.00
3.29
0.87
0.91
2.78
2.98
8.0
7.5
7.0
6.5
6.0
5.5
5.0
4.5
4.0
3.5
3.0
2.5
2.0
1.5
1.0
0.5
0.0
f1 (ppm)

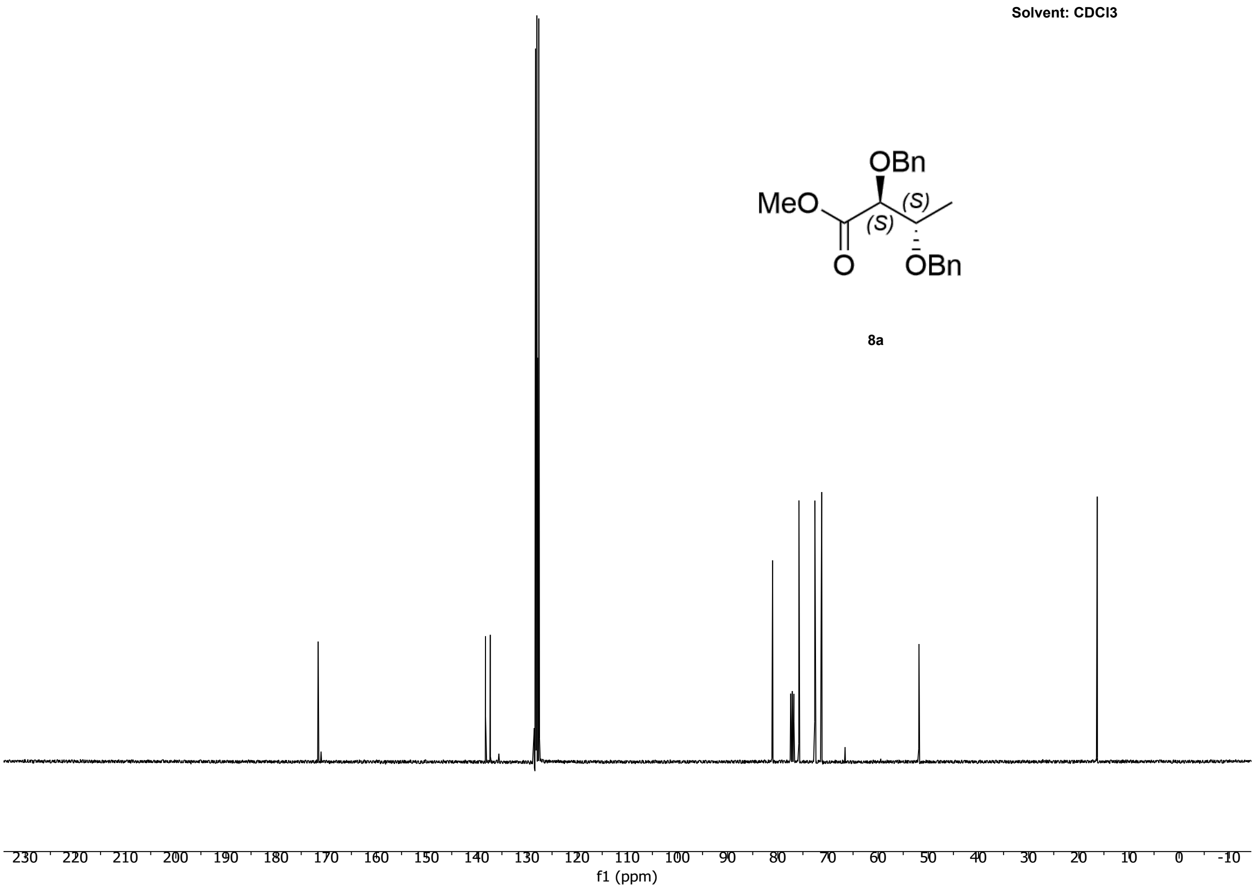
Solvent: CDCl3
OBn
MeO
(S)
(S)
O
OBn
8a
230 220 210 200 190 180 170 160 150 140 130 120 110 100 90 80 70 60 50 40 30 20 10 0 -10
f1 (ppm)

Compound 8b

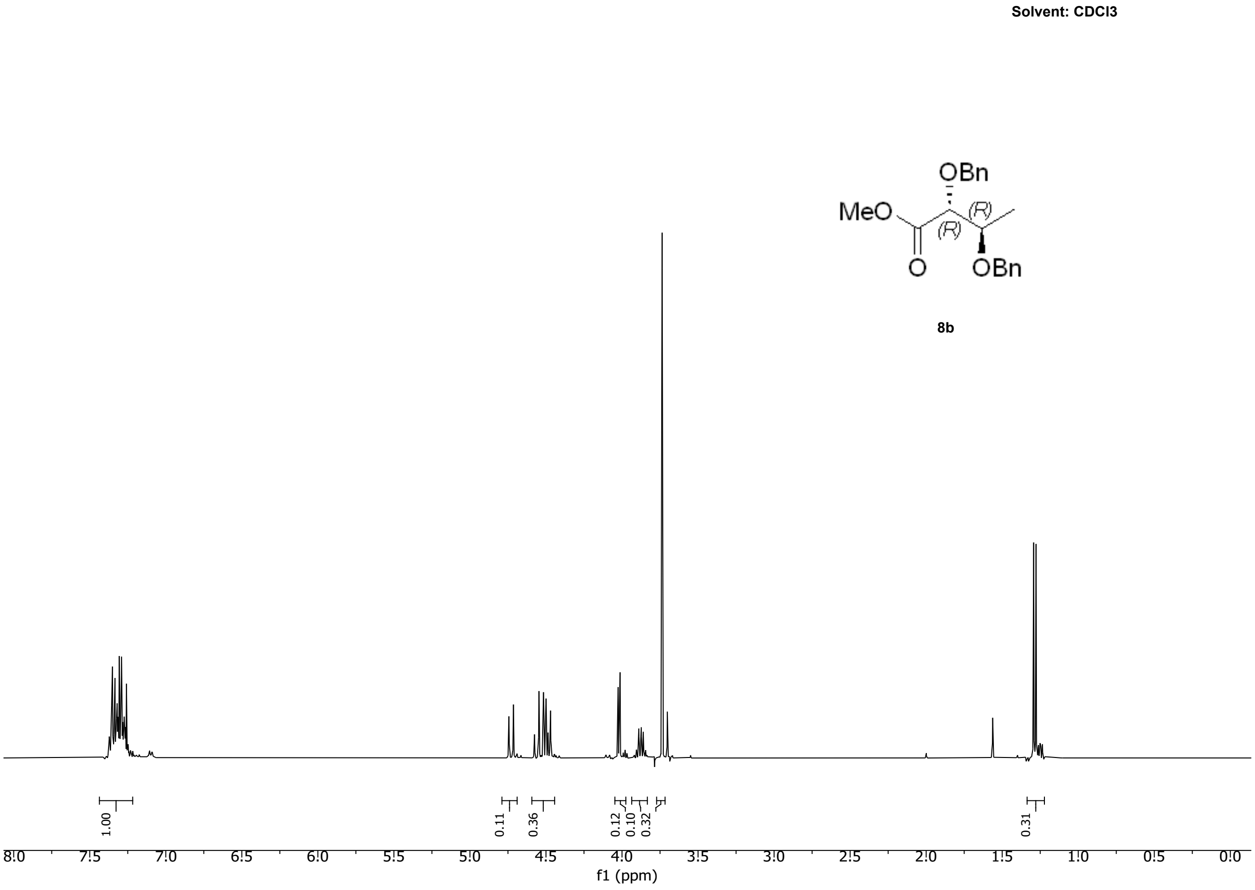
Solvent: CDCl3
MeO
OBn
(R)
(R)
O
OBn
8b
1.00
0.11
0.36
0.12
0.10
0.32
0.31
8.0
7.5
7.0
6.5
6.0
5.5
5.0
4.5
4.0
3.5
3.0
2.5
2.0
1.5
1.0
0.5
0.0
f1 (ppm)

Solvent: CDCl3

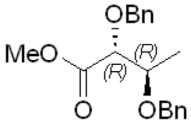

8b

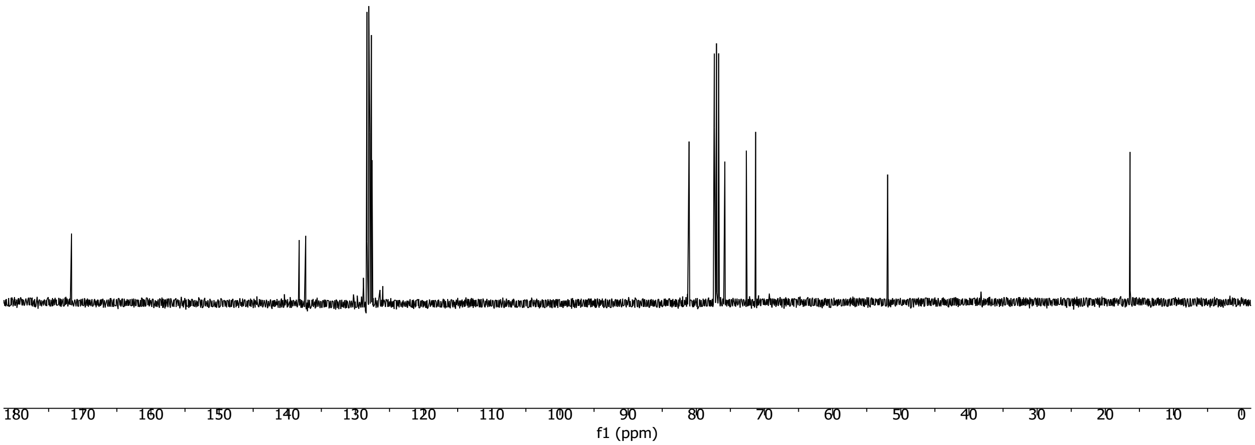

Compound 8c

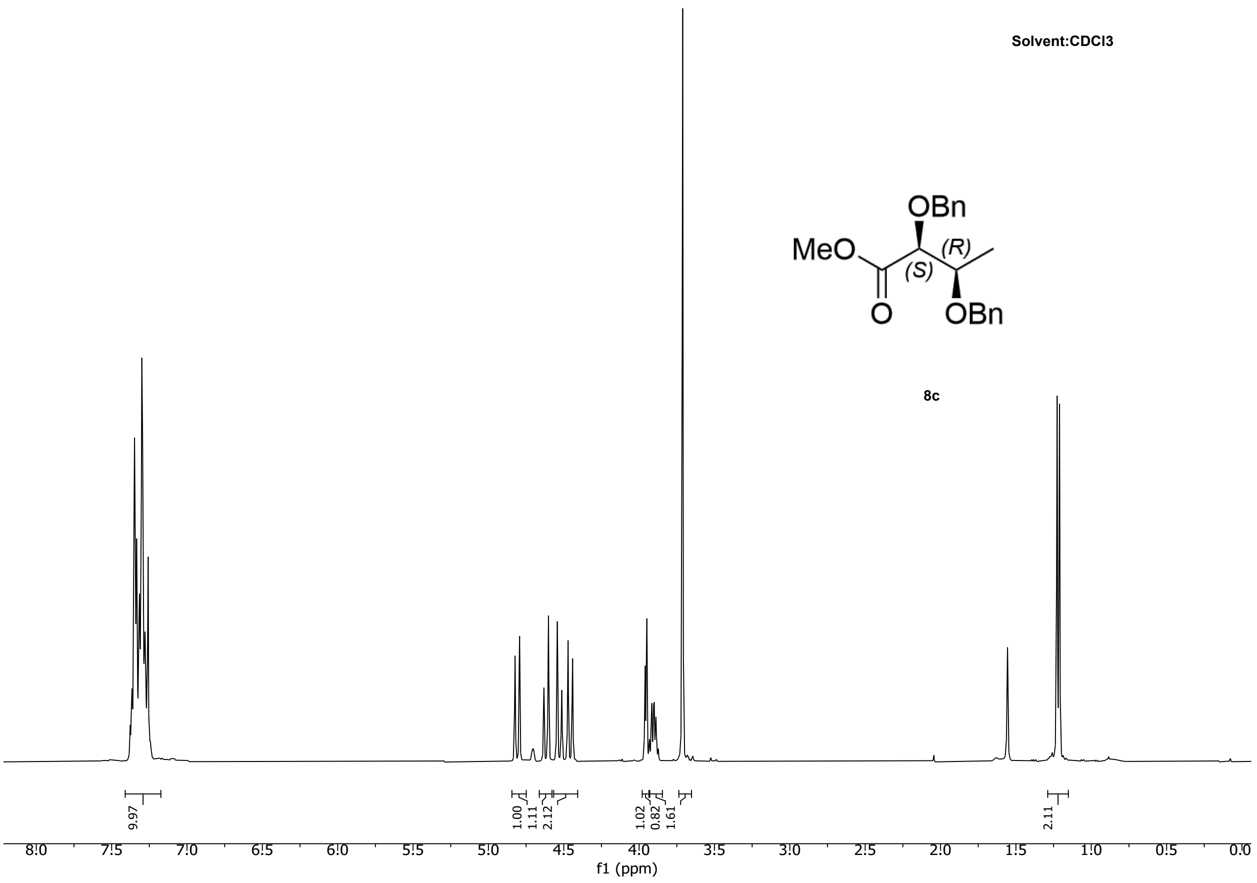

Solvent:CDCl3
MeO
OBn
(R)
(S)
O
OBn
8c
9.97
1.00
1.11
2.12
1.02
0.82
1.61
2.11
8.0
7.5
7.0
6.5
6.0
5.5
5.0
4.5
4.0
3.5
3.0
2.5
2.0
1.5
1.0
0.5
0.0
f1 (ppm)

Solvent:CDCl3

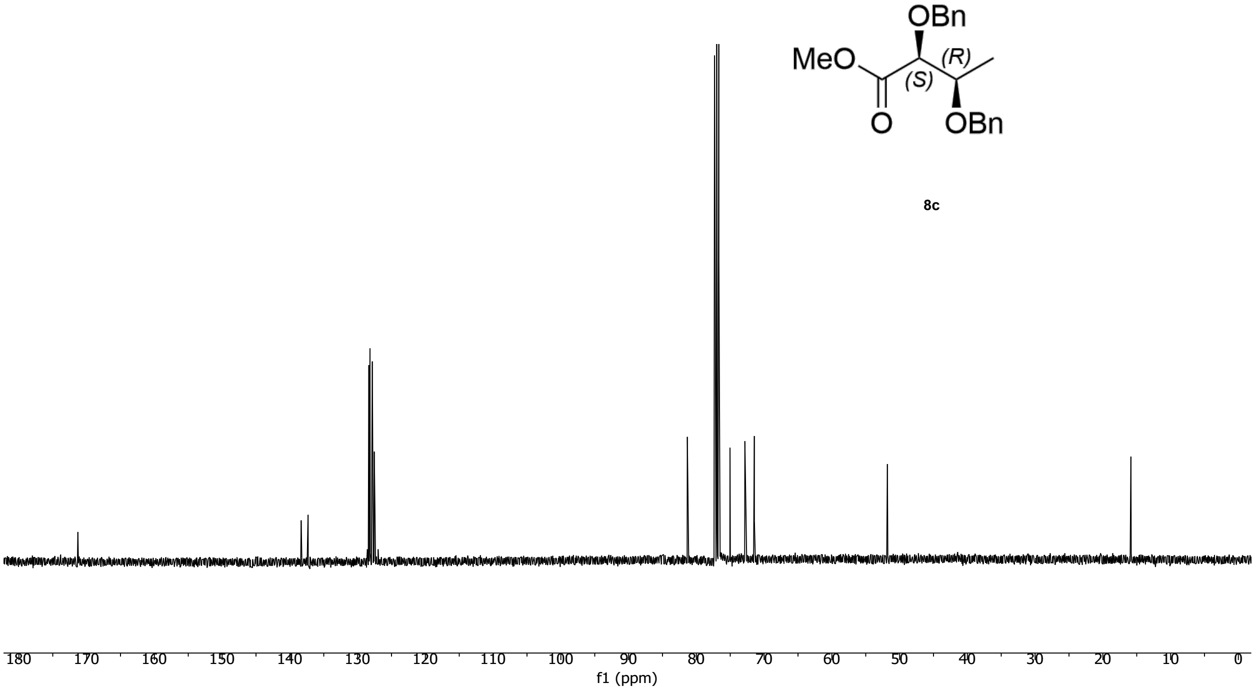

8c

Compound 8d

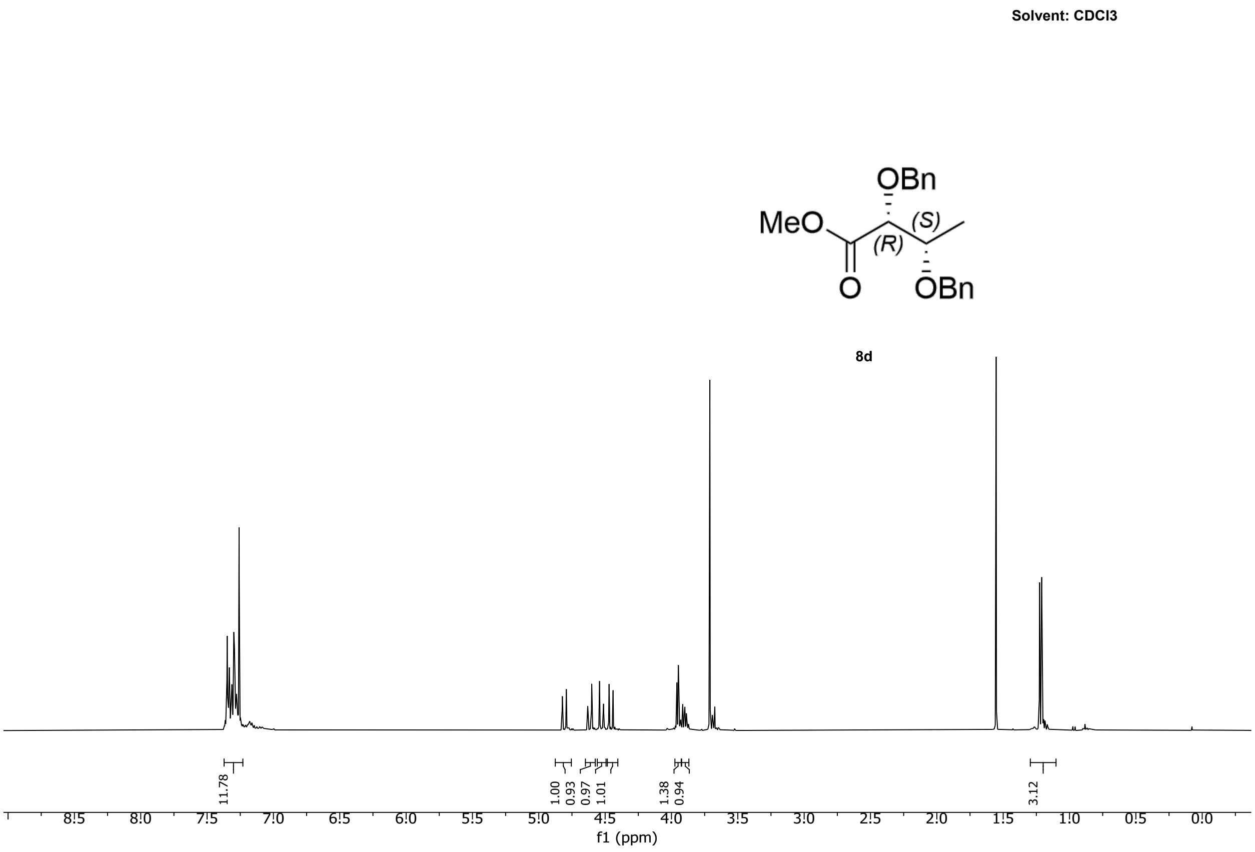
Solvent: CDCl3
OBn
MeO
(R)
(S)
O
OBn
8d
11.78
1.00
0.93
0.97
1.01
1.38
0.94
3.12
8.5
8.0
7.5
7.0
6.5
6.0
5.5
5.0
4.5
4.0
3.5
3.0
2.5
2.0
1.5
1.0
0.5
0.0
f1 (ppm)

Solvent: CDCl3

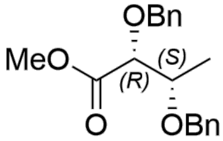

8d

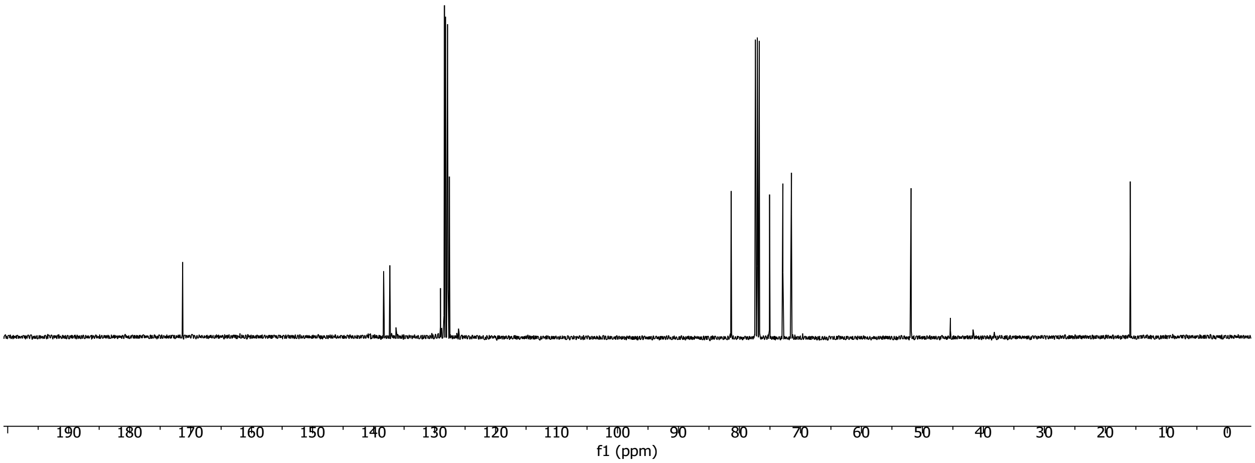

Compound 9a

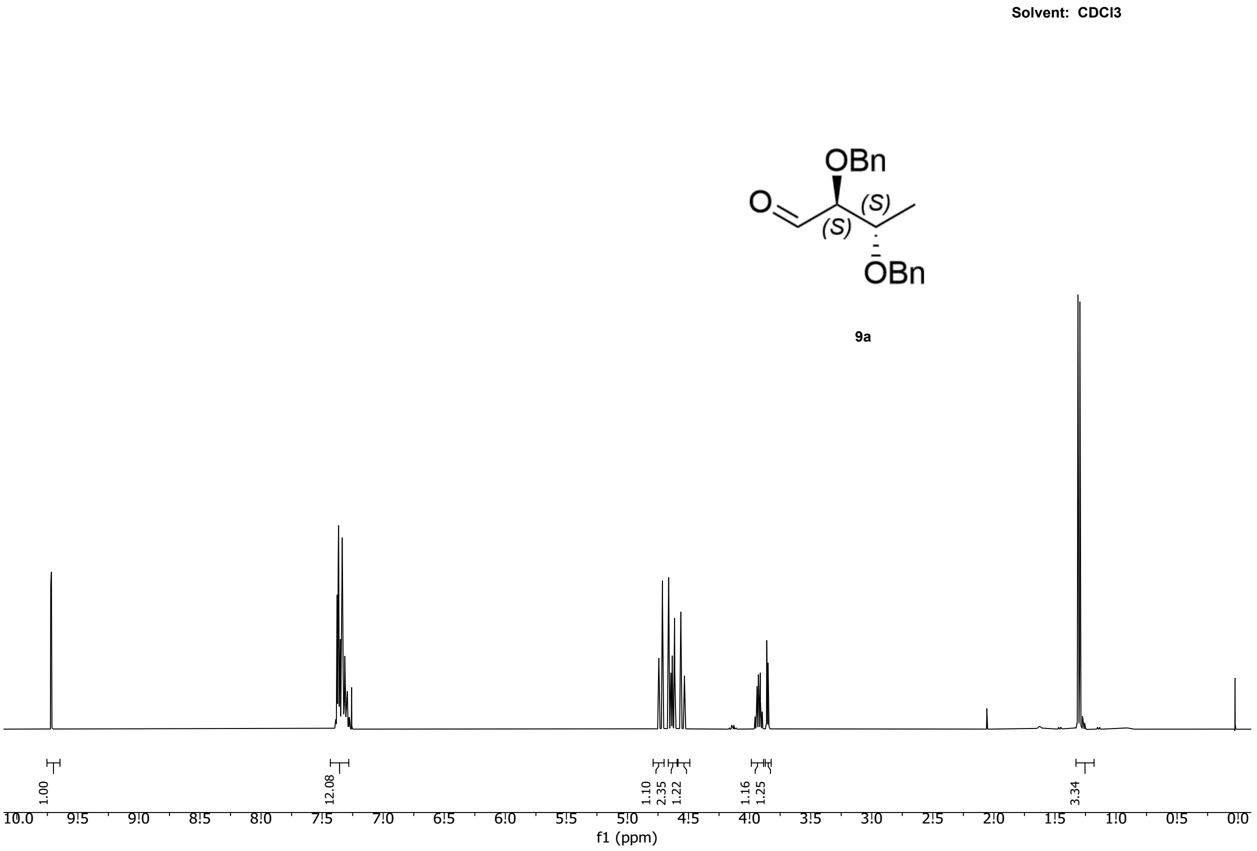
Solvent: CDCl3
OBn
O
(S)
(S)
OBn
9a
1.00
12.08
1.10
2.35
1.22
1.16
1.25
3.34
10.0 9.5 9.0 8.5 8.0 7.5 7.0 6.5 6.0 5.5 5.0 4.5 4.0 3.5 3.0 2.5 2.0 1.5 1.0 0.5 0.0
f1 (ppm)

Solvent: CDCl3

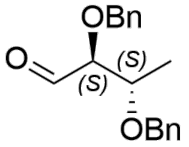

9a

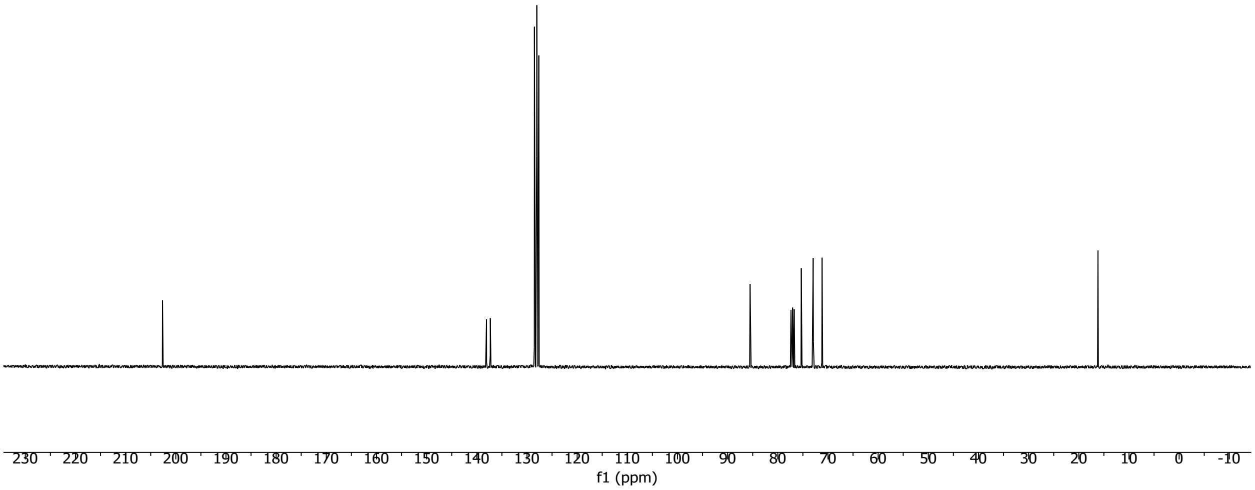

Compound 9b

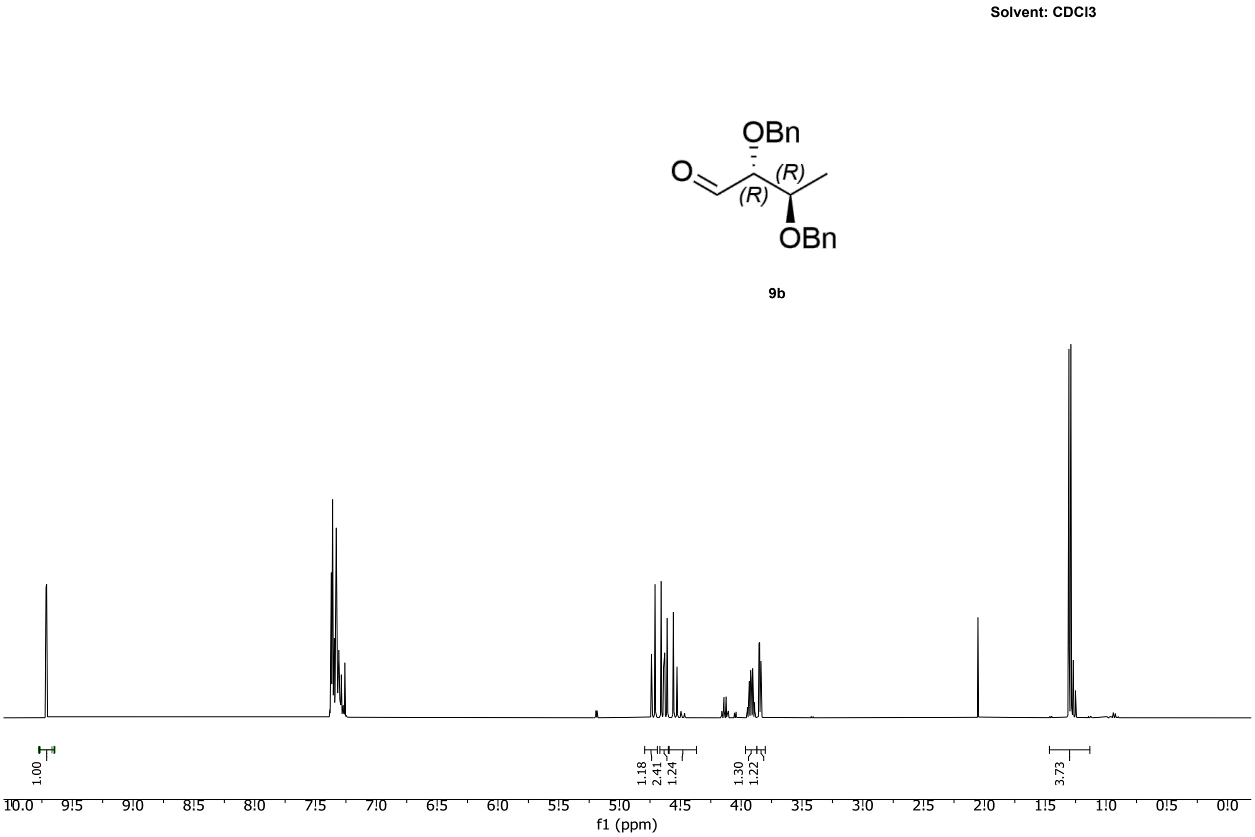
Solvent: CDCl3
OBn
O
(R)
(R)
OBn
9b
1.00
1.18
2.41
1.24
1.30
1.22
3.73
10.0
9.5
9.0
8.5
8.0
7.5
7.0
6.5
6.0
5.5
5.0
4.5
4.0
3.5
3.0
2.5
2.0
1.5
1.0
0.5
0.0
f1 (ppm)

Solvent: CDCl3

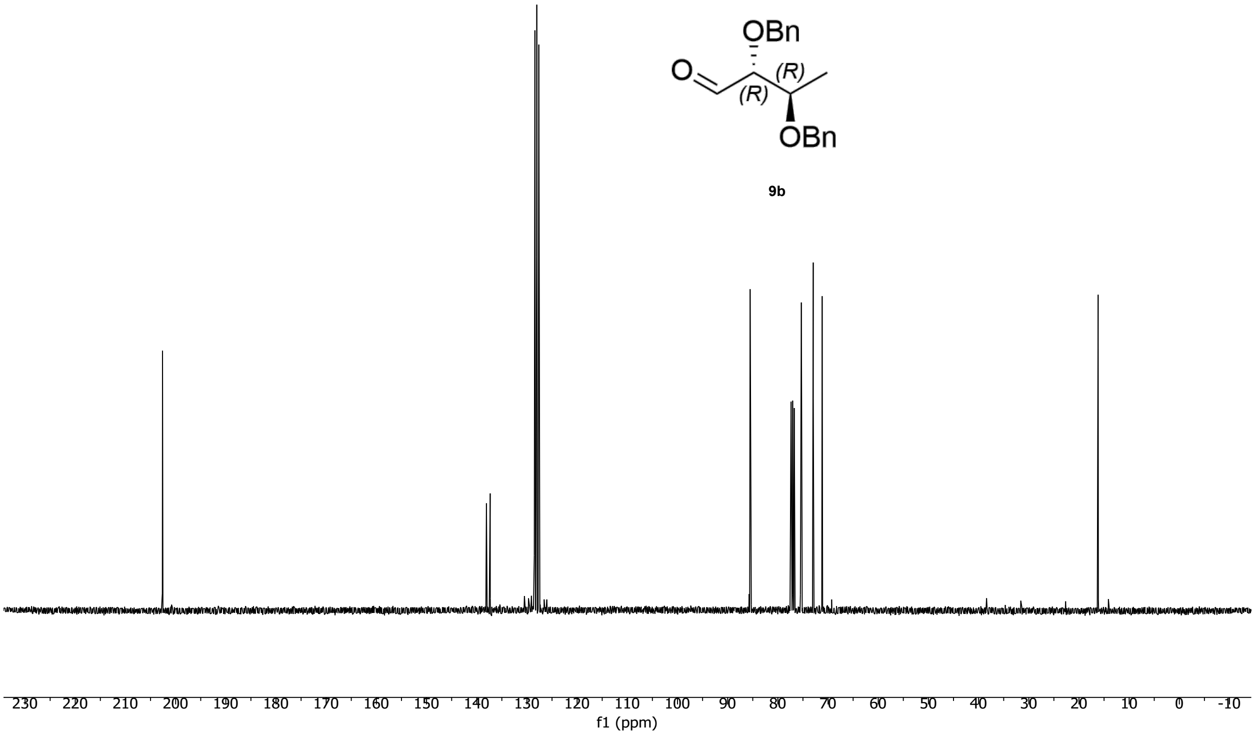

Compound 9c

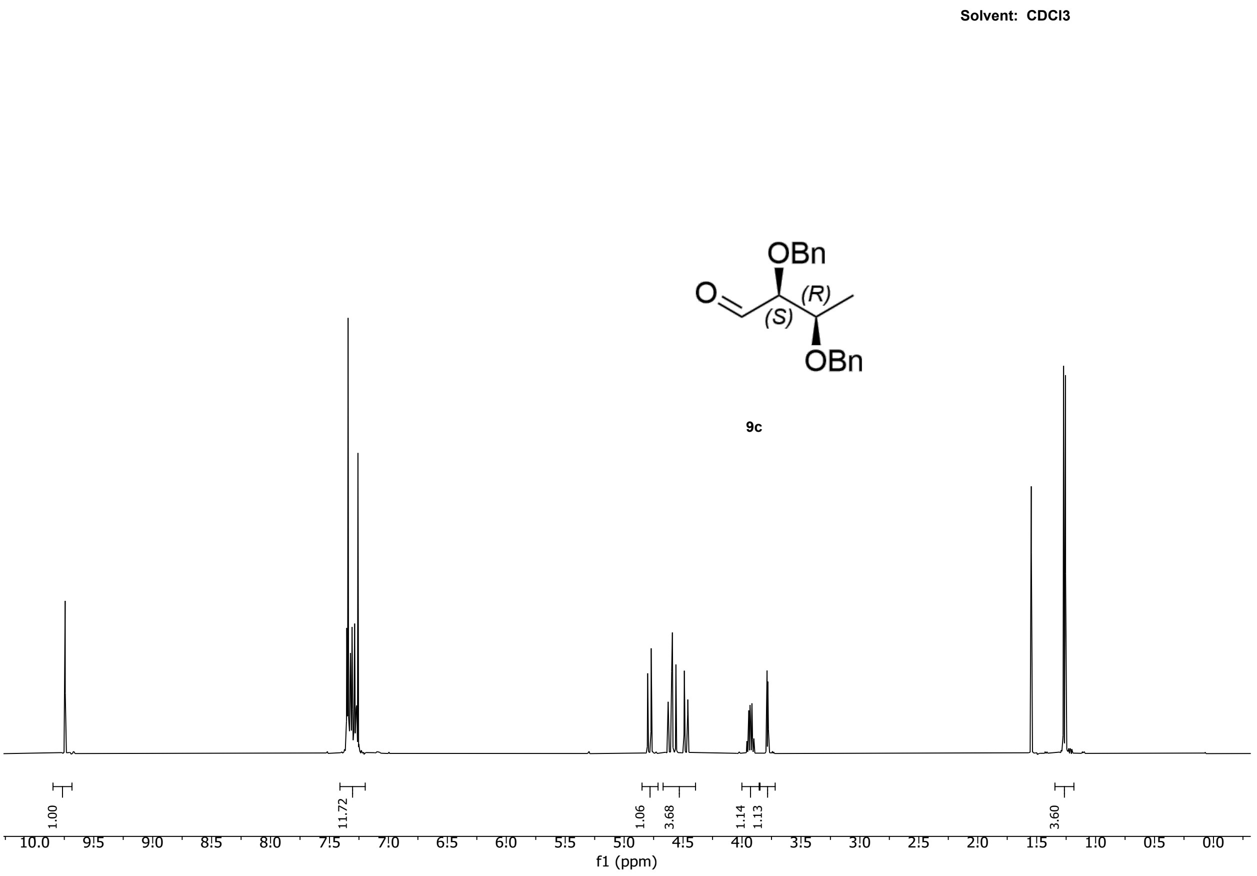

Solvent: CDCl3
OBn
O
(R)
(S)
OBn
9c
1.00
11.72
1.06
3.68
1.14
1.13
3.60
10.0 9.5 9.0 8.5 8.0 7.5 7.0 6.5 6.0 5.5 5.0 4.5 4.0 3.5 3.0 2.5 2.0 1.5 1.0 0.5 0.0
f1 (ppm)

Solvent: CDCl3

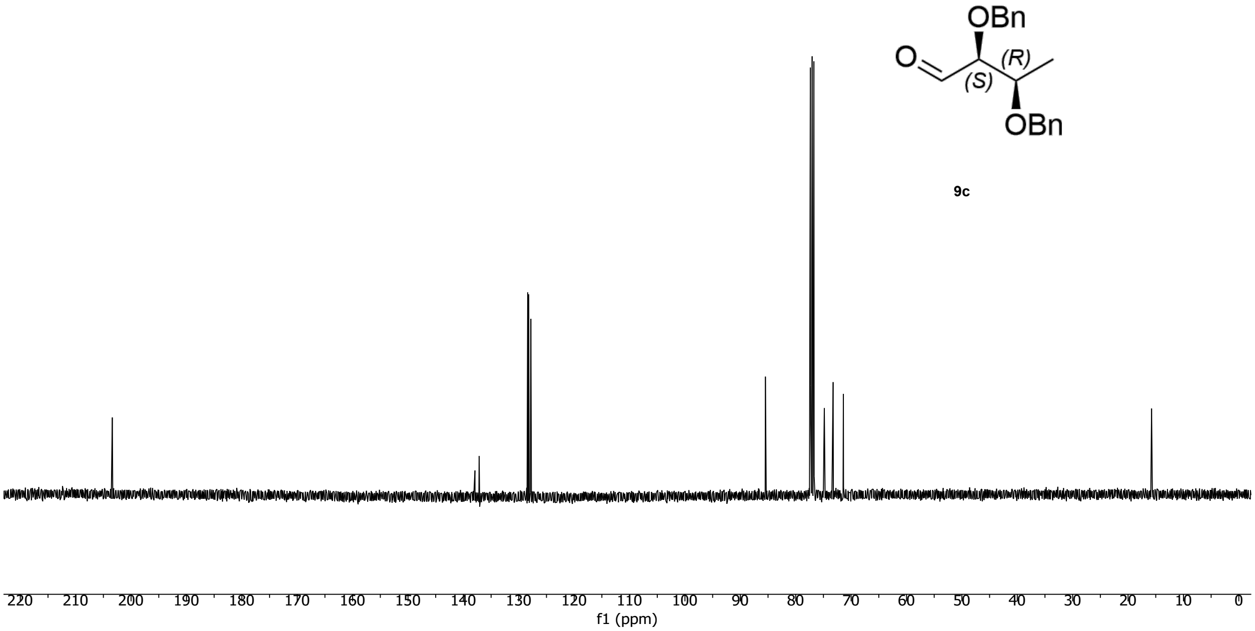

9c

Compound 9d

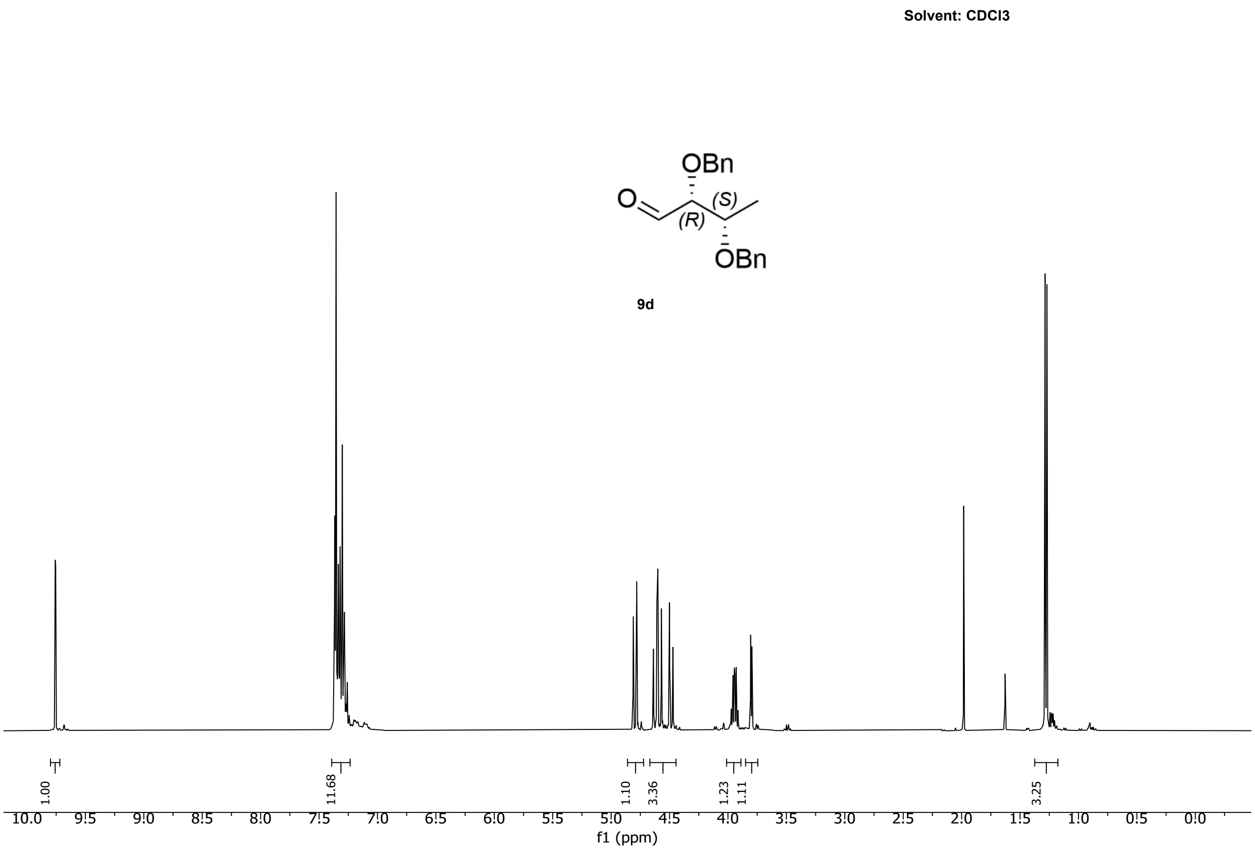

Solvent: CDCl3
OBn
O
(R)
(S)
OBn
9d
1.00
11.68
1.10
3.36
1.23
1.11
3.25
10.0 9.5 9.0 8.5 8.0 7.5 7.0 6.5 6.0 5.5 5.0 4.5 4.0 3.5 3.0 2.5 2.0 1.5 1.0 0.5 0.0
f1 (ppm)

Solvent: CDCl3

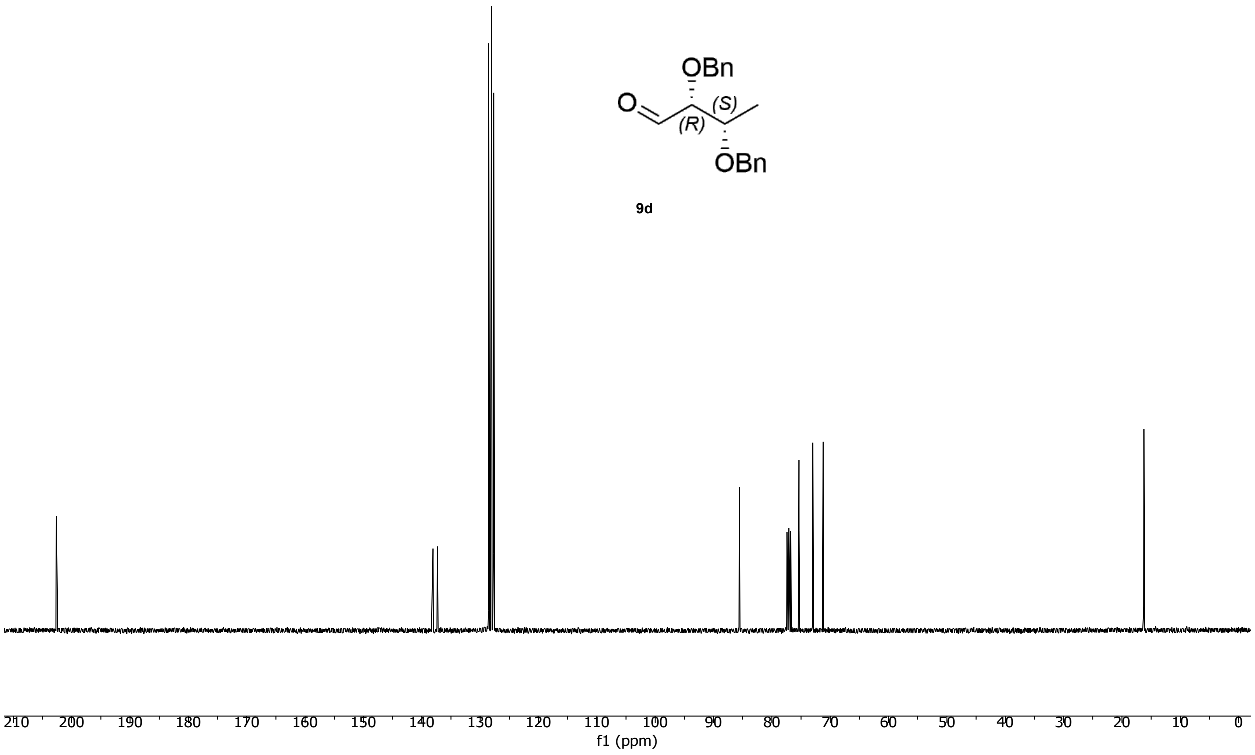

9d

Compound 11

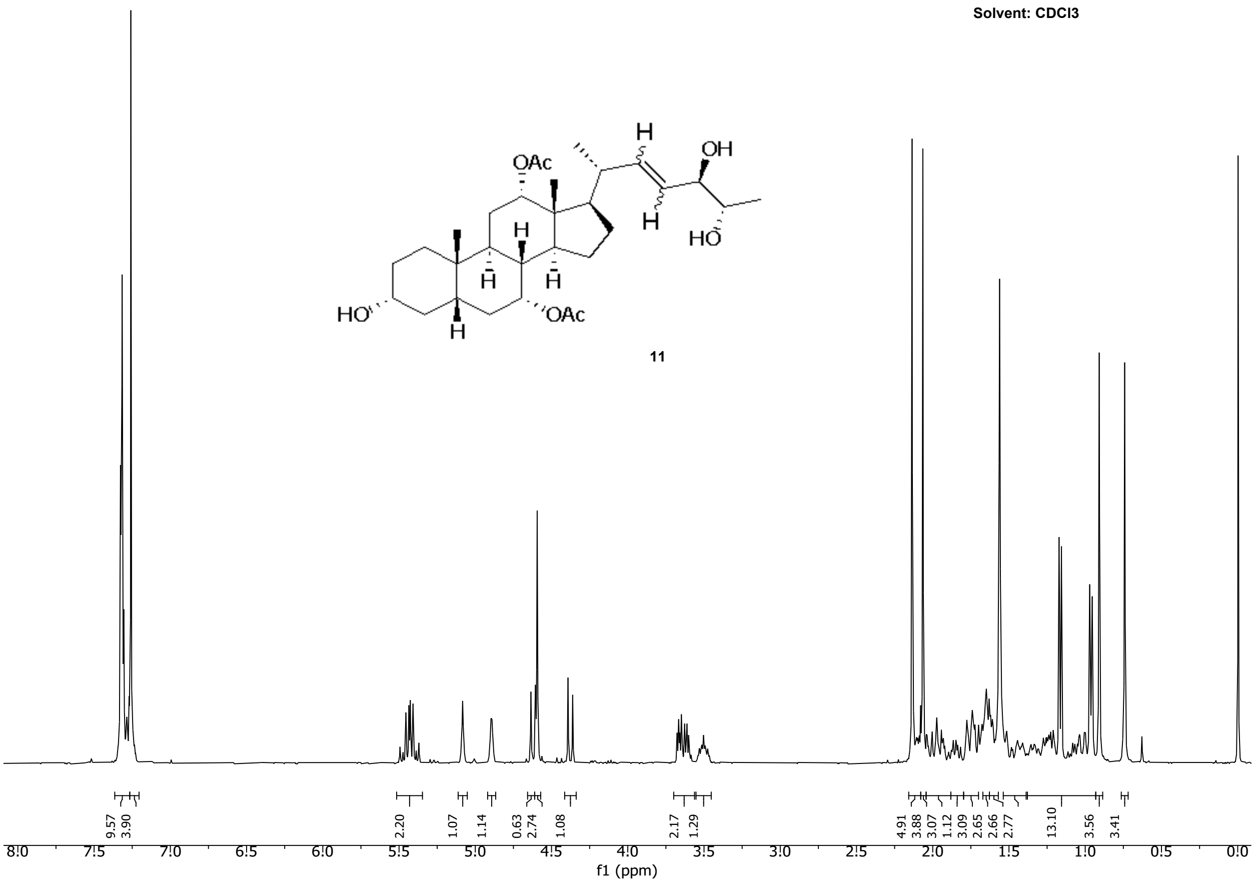
Solvent: CDCl3
OAc
OH
HO
H
OAc
11
9.57
3.90
2.20
1.07
1.14
0.63
2.74
1.08
2.17
1.29
4.91
3.88
3.07
1.12
3.09
2.65
2.66
2.77
13.10
3.56
3.41
8.0
7.5
7.0
6.5
6.0
5.5
5.0
4.5
4.0
3.5
3.0
2.5
2.0
1.5
1.0
0.5
0.0
f1 (ppm)

Solvent: CDCl3

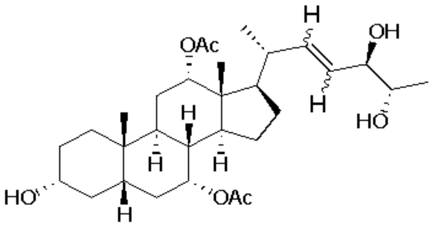

11

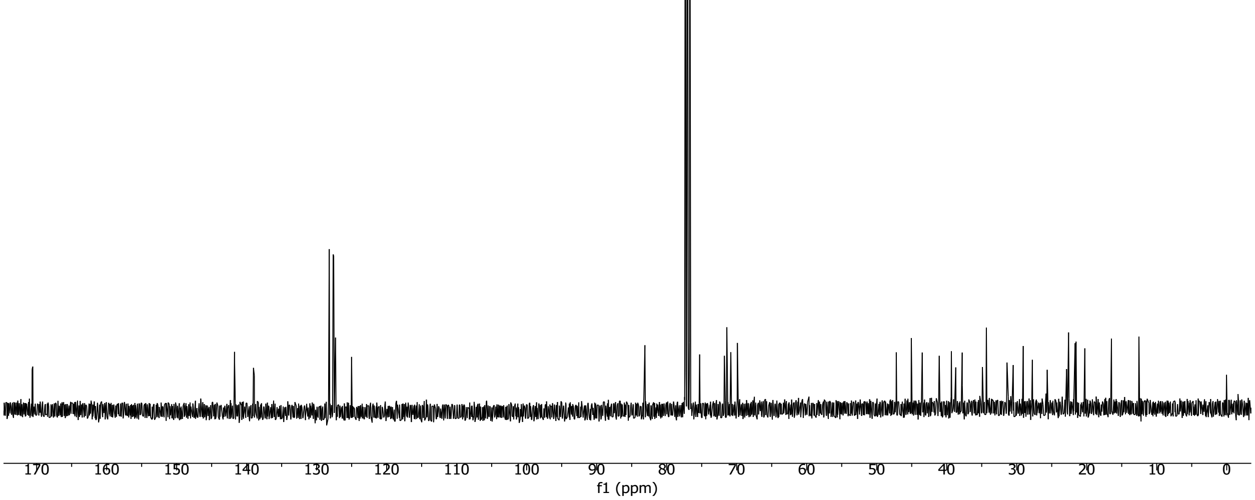

Compound 13

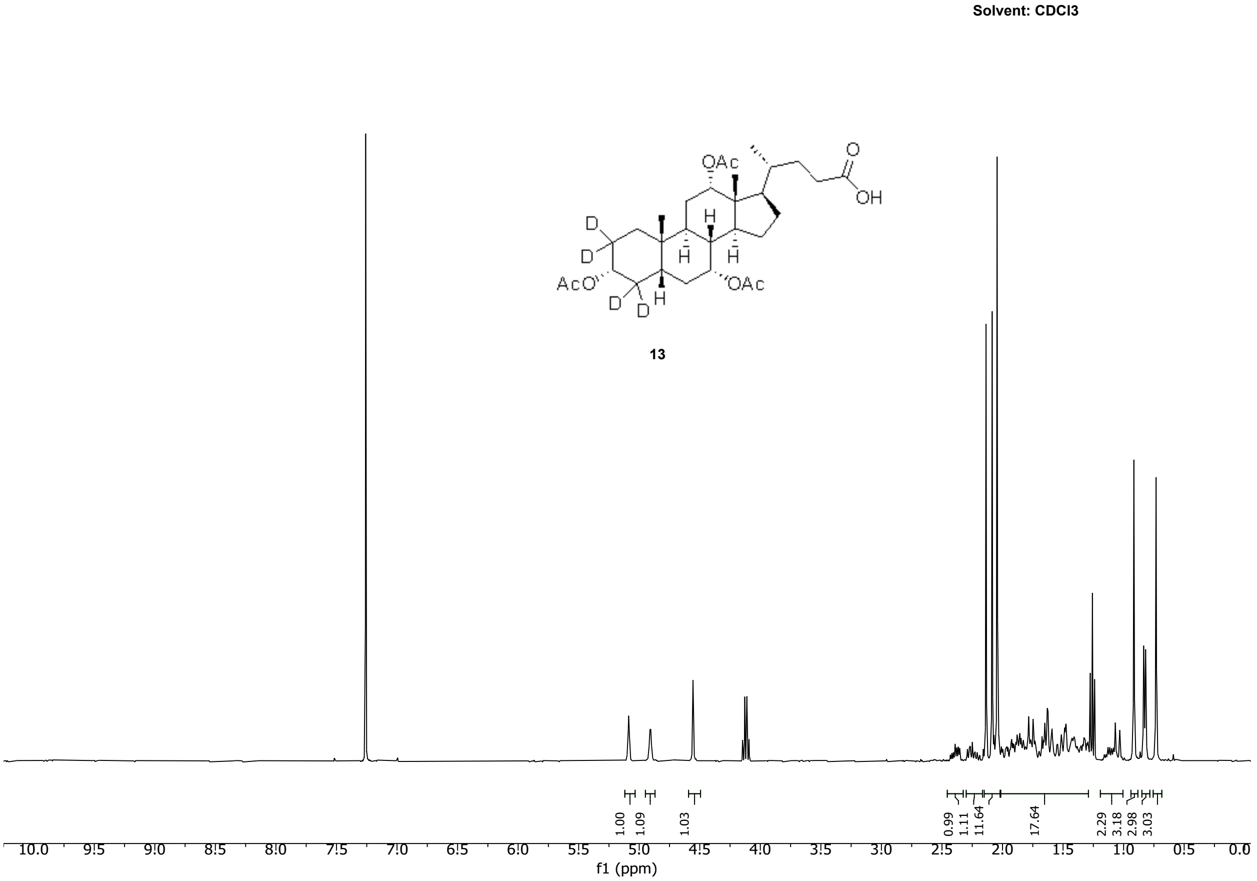

Solvent: CDCl3
OAc
O
OH
D
D
AcO
D
D
H
H
H
H
OAc
13
1.00
1.09
1.03
0.99
1.11
11.64
17.64
2.29
3.18
2.98
3.03
10.0
9.5
9.0
8.5
8.0
7.5
7.0
6.5
6.0
5.5
5.0
4.5
4.0
3.5
3.0
2.5
2.0
1.5
1.0
0.5
0.0
f1 (ppm)

Compound 14

Solvent: CDCl3

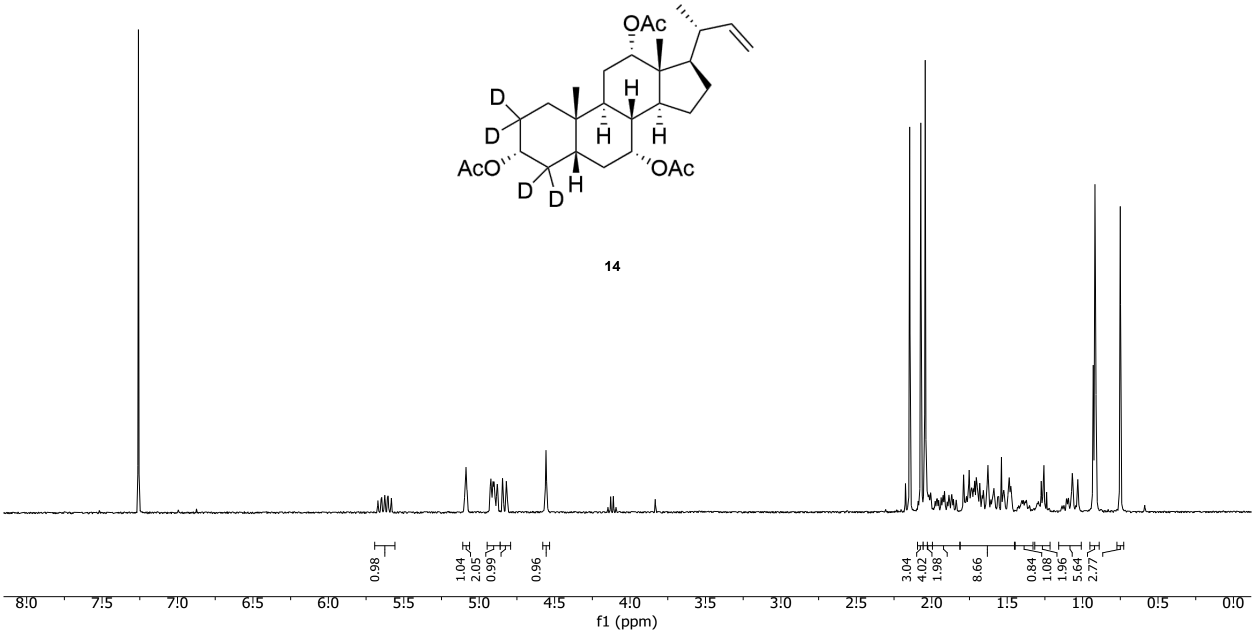

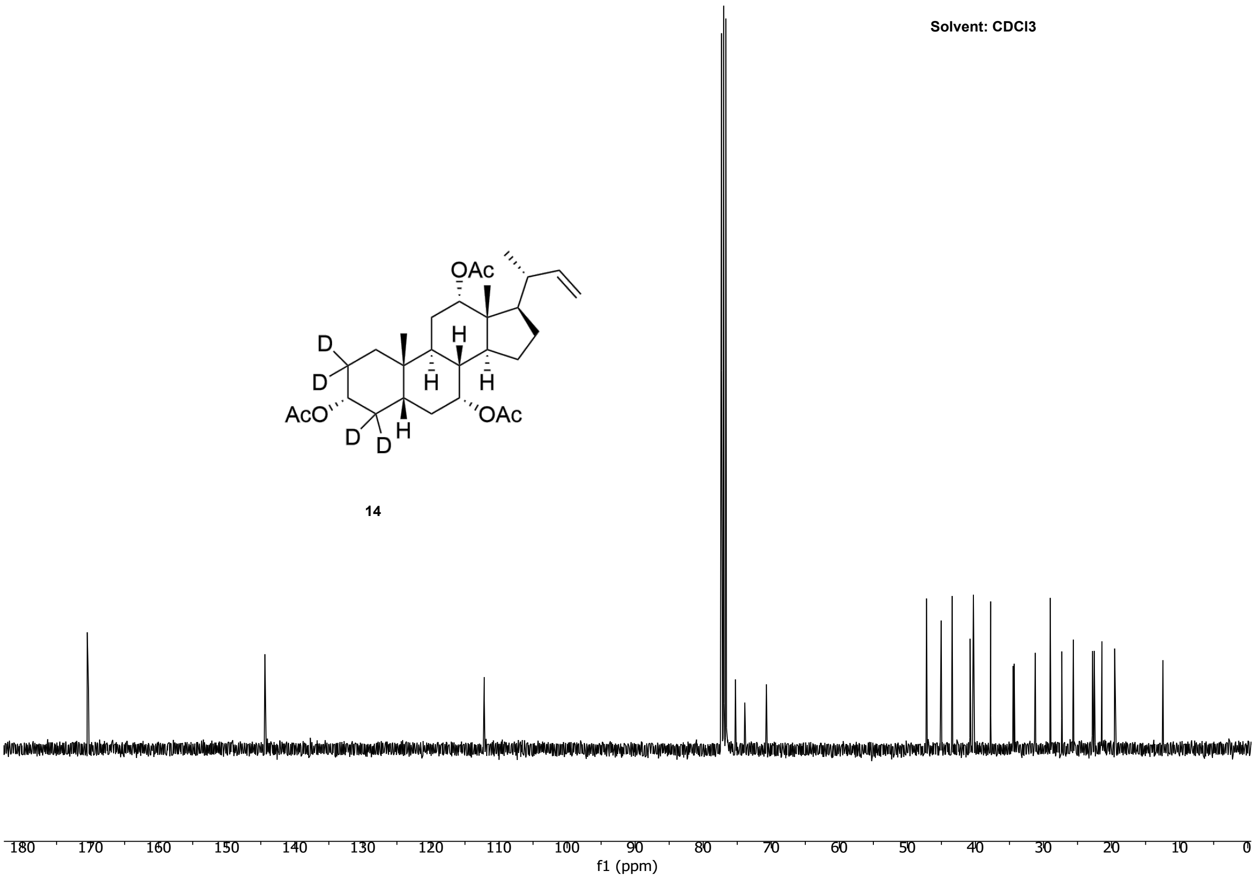

Solvent: CDCl3
D
D
AcO
D
D
H
H
H
H
OAc
OAc
14
180
170
160
150
140
130
120
110
100
90
80
70
60
50
40
30
20
10
0
f1 (ppm)

Compound 16

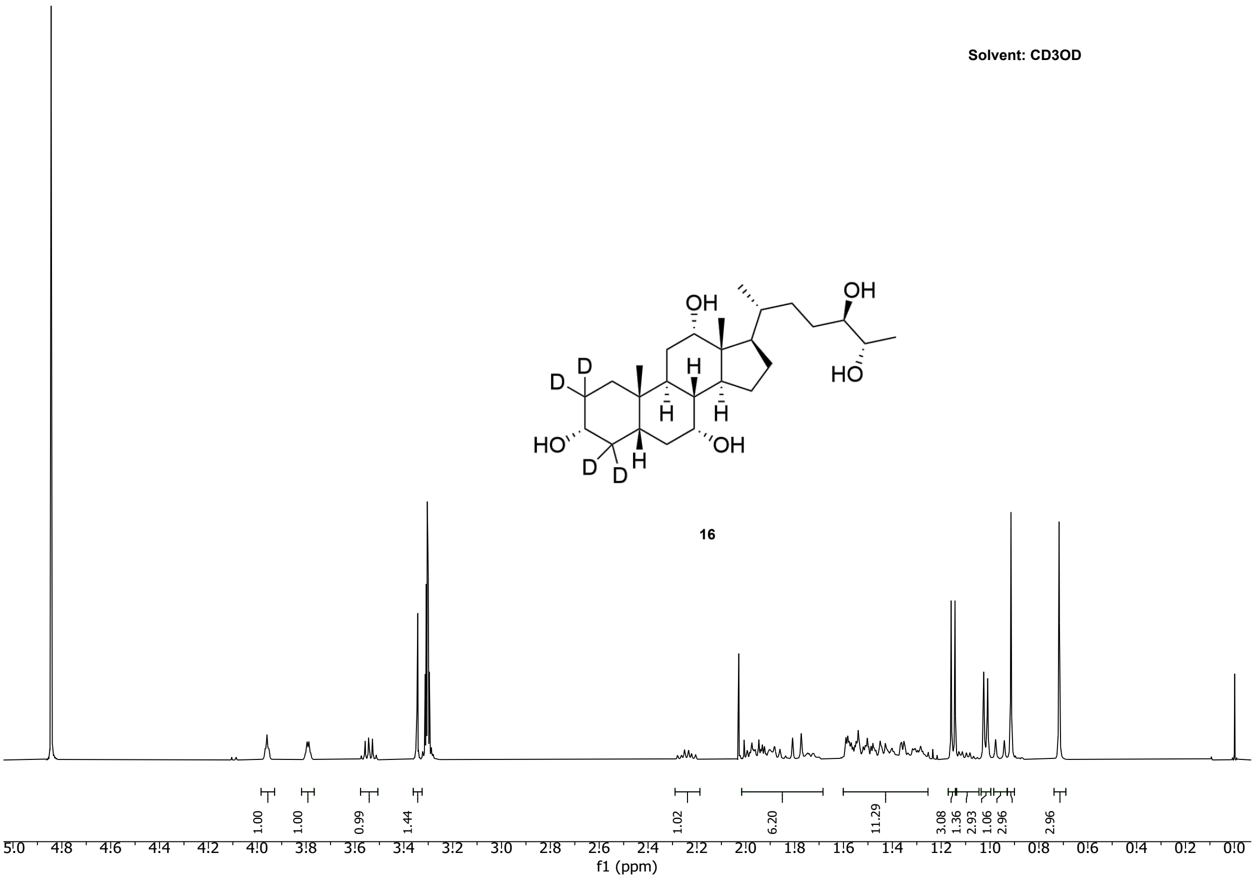
Solvent: CD3OD
16
1.00
1.00
0.99
1.44
1.02
6.20
11.29
3.08
1.36
2.93
1.06
2.96
2.96
5.0 4.8 4.6 4.4 4.2 4.0 3.8 3.6 3.4 3.2 3.0 2.8 2.6 2.4 2.2 2.0 1.8 1.6 1.4 1.2 1.0 0.8 0.6 0.4 0.2 0.0
f1 (ppm)

Solvent: CD3OD

16

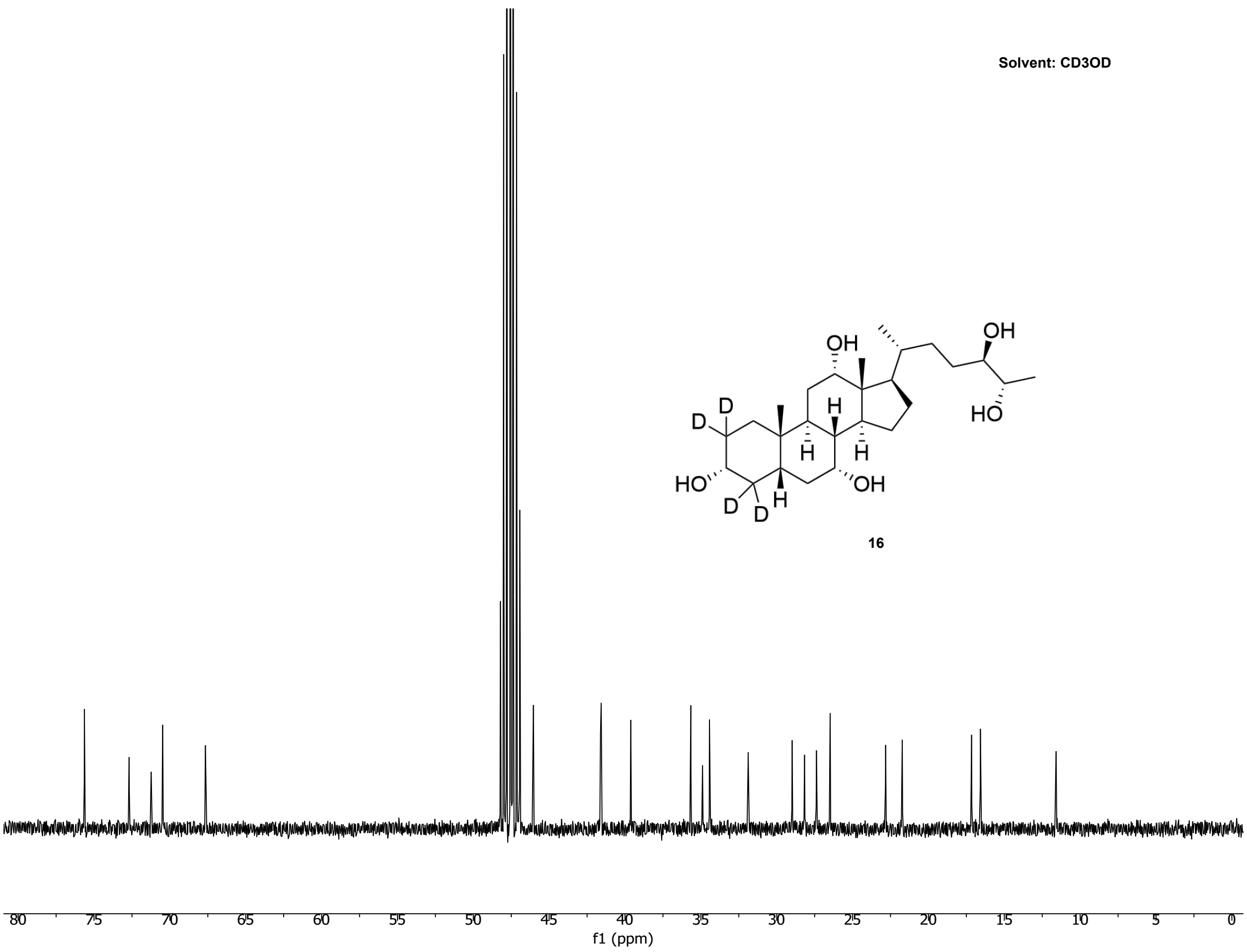

Supplement: Supplementary file 1 [file molecules-29-02781-s001.zip › molecules-3022576-supplementary.pdf]
